# Dynamic Mosaicity Modulates Ion Transport in Stimuli‐Responsive Liquid Crystal Electrolytes

**DOI:** 10.1002/advs.202510610

**Published:** 2025-08-11

**Authors:** Hélène Pung, Celso Yassuo Okada‐Junior, Mirella Simões Santos, Marta Mirolo, Isabelle Morfin, Gilbert Chahine, Jannick Duchet‐Rumeau, Johan Jacquemin, Agilio Padua, Sébastien Livi, Patrice Rannou, Manuel Maréchal

**Affiliations:** ^1^ Univ. Grenoble Alpes, CNRS, CEA, Grenoble‐INP, IRIG SyMMES Grenoble 38000 France; ^2^ Univ. Claude Bernard Lyon 1, INSA Lyon, Univ. Jean Monnet, CNRS UMR 5223 Ingénierie des Matériaux Polymères Lyon Cédex 69621 France; ^3^ Laboratoire de Chimie de l'ENS Lyon CNRS and Université de Lyon 46 allée d'Italie Lyon 69364 France; ^4^ European Synchrotron Radiation Facility 71 Avenue des Martyrs Grenoble 38000 France; ^5^ Univ Grenoble Alpes, CNRS LiPhy Grenoble 38000 France; ^6^ Univ. Grenoble Alpes, CNRS, Grenoble‐INP SIMAP Grenoble 38000 France; ^7^ Laboratory of Physical‐Chemistry of Materials and Electrolytes for Energy (PCM2E) University of Tours Tours 37200 France; ^8^ Univ. Grenoble Alpes, Université Savoie Mont‐Blanc, CNRS, Grenoble‐INP LEPMI Grenoble 38000 France; ^9^ Present address: MSN Department VI Polytechnic University Lot 660, Hay Moulay Rachid, Mohammed Ben Guerir 43150 Morocco

**Keywords:** anisotropic ionic transport, dynamic mosaicity, *in situ/* *operando* correlations, nanoscale confinement, supramolecular ordering, thermotropic ionic liquid crystals

## Abstract

Structural mosaicity and defects are ubiquitous across materials and critically influence functional properties, from semiconductors to biological membranes. In soft matter electrolytes, these features remain difficult to probe and exploit due to complex synthesis and limited long‐range structural order. A dimensionally tunable model system based on thermotropic ionic liquid crystals (TILCs) is introduced to investigate the interplay between structural mosaicity and ion transport. In two‐dimensional (2D) anion‐conducting smectic TILCs, ion transport exhibits a pronounced anisotropy—up to four orders of magnitude at 70 °C—attributed to ion confinement within 0.7–1.2 nm‐thick lamellar sublayers. In situ and *operando* synchrotron X‐ray scattering combined with electrochemical analyses reveal direct experimental evidence of a strong correlation between long‐range supramolecular organization, quantified via dynamic mosaicity, and mesoscopic ion transport. Application of a 1 Tesla magnetic field enhances domain size by 1.5× and boosts conductivity threefold, demonstrating stimuli‐responsive, mosaicity‐controlled ionic transport. These findings establish a generalizable structure–function framework for confined ion conduction in soft materials and bridge concepts from thermotropic liquid crystals to lyotropic phases, biological assemblies, and organic semiconductors. Positioning dynamic mosaicity as a key design parameter, this work lays the foundation for rational development of adaptive, self‐organized electrolyte systems for energy storage and conversion, ionotronics, and bioinspired ionic devices.

## Graphical abstract

Thermotropic ionic liquid crystals provide a tunable platform for dimensionally‐controlled soft electrolytes, where anisotropy, nanoconfinement, and dynamic mosaicity govern ion transport behavior. In situ and *operando* analyses reveal a direct correlation between dynamic mosaicity and mesoscopic conductivity. Magnetic field alignment enhances domain size and amplifies directional transport, establishing mosaicity as a design lever in stimuli‐responsive electrolytes.

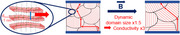

## 1 Introduction

Understanding the fundamental mechanisms governing ion transport in dimensionally‐controlled soft matter electrolytes is critical for the design of functional materials for applications in electrochemical energy storage,^[^ ^1^, ^2^ ^]^ including next‐generation batteries^[^ ^3^ ^]^ and conversion devices,^[^ ^4^ ^]^ biology,^[^ ^5^, ^6^, ^7^ ^]^ nanoelectronics,^[^ ^8^ ^]^ nanoionics,^[^ ^9^ ^]^ and nanofluidics.^[^ ^10^, ^11^ ^]^ However, unravelling the role of structural mosaicity and dynamic disorder in nanoscale ion transport remains a challenge due to the inherent complexity of probing and isolating these finely tuned structural features in self‐assembled soft materials.

Stimuli‐responsive model systems with controllable structural order are essential to access robust structure–transport correlations under *operando* conditions. Among them, thermotropic ionic liquid crystals (TILCs) offer a rare combination of dimensional control, dynamic molecular ordering, and structural tunability, making them an ideal platform to study ion transport in soft functional materials.

### 1.1 Thermotropic Ionic Liquids Crystals (TILCs)

Thermotropic Liquid Crystals (TLCs)^[^ ^12^, ^13^, ^14^ ^]^ are dynamically self‐assembled materials^[^ ^15^, ^16^, ^17^ ^]^ with intrinsic stimuli‐responsiveness. They elegantly combine fluidity of their mesophases with *tunable‐by‐design* ^[^ ^18^ ^]^ controlled supramolecular organizations in which precise (quasi‐)1D vs. 2D vs. 3D ordering can be ultimately achieved (See Figure S1, Supporting Information). From their discovery in 1888^[^ ^19^ ^]^ by the Austrian Botanist F. Reinitzer (Cholesterol/Cholesteryl benzoate), through a ca. 40 years long intense scientific debate about their existence, with controversies till the 1930's, they gradually developed over the 20^th^ century into a lively and truly multidisciplinary (chemistry and physics) research fields.^[^ ^13^ ^]^ Their successful transfer into the LCD industry^[^ ^20^, ^21^ ^]^ in the early 1970s accompanied the transformative microelectronics revolution. TLCs thus represent a fascinating class of materials, both from the fundamental and applied research points of view.^[^ ^22^ ^]^ While the LCD‐oriented research is on the wane,^[^ ^13^ ^]^ basic‐oriented research on functional TLCs^[^ ^12^, ^14^ ^]^ is flourishing. This trend towards functionality is illustrated by the cutting‐edge research developed around thermotropic liquid crystalline organic semiconductors (See reviews^[^ ^23^, ^24^, ^25^ ^]^) and TILCs (See reviews^[^ ^26^, ^27^, ^28^ ^]^). Among these functional mesomorphic materials, TILCs represent the fusion of Ionic Liquids (ILs)^[^ ^29^, ^30^ ^]^ and TLCs, combining the advantages of these two complementary fields. The first ever reported TILC was likely discussed by W. Heintz in 1855,^[^ ^30^, ^31^ ^]^ who noticed melting and clearing transitions upon heating of magnesium myristate, i.e., 34 years before F. Reinitzer seminal work on liquid crystals.^[^ ^13^, ^19^ ^]^

Several pioneering contributions have since marked the TILCs’ development. Notably, the first report of pyridinium‐based TILCs published by Knight and Shaw in 1938,^[^ ^32^ ^]^ as well as the very important contributions from A. Skoulios in the 1960–1970s on “metals salts”.^[^ ^33^, ^34^, ^35^ ^]^ The Strasbourg school of TILCs remains very active, particularly in the study of imidazolium‐^[^ ^36^ ^]^ and bisimidazolium‐^[^ ^37^ ^]^ based TILCs. Contemporary research on TILCs gained momentum following the seminal report by D.W. Bruce and K.R. Seddon on mesomorphic ionic liquids.^[^ ^38^ ^]^ Recent developments on TILCs have been widely reported in several reviews published over the last few decades by leading researchers, including K. Binnemans,^[^ ^26^ ^]^ S. Laschat,^[^ ^27^ ^]^ P.H.J. Kouwer,^[^ ^39^ ^]^ and T. Kato,^[^ ^14^, ^40^ ^]^ who have made significant contributions to the fields of ILs, TLCs, and TILCs.

Due to their particular combination of properties, TILCs^[^ ^12^, ^14^, ^25^, ^26^, ^27^, ^28^, ^39^, ^40^ ^]^ have recently been the subject of intense research as stimuli‐responsive functional self‐assembled materials for ion‐selective membranes, as well as soft matter‐based electrolytes.^[^ ^41^, ^42^ ^]^ Over the past two decades, T. Kato and co‐workers have developed a full and comprehensive range of TILCs whose specific quasi‐1D,^[^ ^43^, ^44^, ^45^, ^46^ ^]^ 2D,^[^ ^47^, ^48^, ^49^, ^50^ ^]^ or 3D^[^ ^51^, ^52^, ^53^ ^]^ mesophases, enabling the first in‐depth studies of structure/ion transfer interplay. Remarkably, this innovative group has recently extended its achievements to the design of soft organic electrolytes for batteries, through a mixing approach, by blending lithium salts with a carbonate‐based LC matrix.^[^ ^54^ ^]^ Controlled switching of ionic conductivities^[^ ^45^ ^]^ in a TILC consisting of wedge‐shaped LC ammonium salts has been produced through a thermoreversible phase transition between its rectangular columnar (Col_rec_) and hexagonal columnar (Col_hex_) mesophases. Within the inspiring vein triggered by the ground‐breaking work of V. Percec and co‐workers^[^ ^55^ ^]^ on tapered‐shaped LC self‐assemblies and dendron/dendritic‐based LCs, including TILCs,^[^ ^55^ ^]^ M. Möller, U. Beginn, and D. Ivanov have contributed to the development of a fascinating family of wedge‐shaped sulfonic acid‐based TILCs for dimensionality‐controlled proton and ion transfer in soft‐matter‐based self‐assemblies.^[^ ^56^, ^57^, ^58^, ^59^ ^]^ These authors have also recently highlighted the equally important influence of the chemical structure of TILCs and counterions on the supramolecular organization and ion transfer capacity.^[^ ^60^ ^]^

To summarize, numerous ‐ and truly inspiring ‐ achievements in TILC's design‐to‐function have been described. However, with a few notable exceptions^[^ ^61^ ^]^ in the literature, TILCs^[^ ^13^, ^14^, ^40^, ^62^ ^]^ developed to date still spontaneously self‐assemble into controlled but polydomain‐like supramolecular organizations in which mosaicity impacts ion transport ability.^[^ ^63^, ^64^ ^]^ Most of the ionic conductivities measured so far are only effective ones, i.e., averaged over randomly oriented domains connected via dynamic homophase grain boundaries (GBs). The intrinsic (i.e., disorder/heterogeneity‐free) properties of these ionically conducting materials, in terms of ionic selectivity and transport, are therefore not fully exploited and remain therefore basically unknown. Nevertheless, the mosaicity observed in our system can be conceptually linked to grain boundary effects commonly discussed in conductors, particularly in organic semiconductors, where structural defects modulate electronic transport properties. In semiconducting thermotropic liquid crystals, both discotic^[^ ^65^ ^]^ and calamitic^[^ ^66^, ^67^ ^]^ types, mesophase organization and defect structures have been shown to critically influence (hole and electron) charge carrier mobility. These effects are further contextualized in broader studies on charge transport in self‐assembled soft materials.^[^ ^68^ ^]^ While our study focuses on ionic rather than electronic conductivity, similar principles apply, particularly regarding the role of anisotropy and confinement in tuning directional transport. However, the presence of mosaicity significantly hinders our ability to assess the intrinsic impact of nanoscale soft confinement on ionic behavior and prevents effective benchmarking of TILC performance in the context of molecular engineering. Realizing long‐range ordering of grain boundary, free TILCs at the micron scale, commensurate with typical electrode separations in energy conversion and storage devices—thus presents a substantial opportunity to deepen fundamental understanding and drive breakthrough innovations.

### 1.2 Long‐Range Organization

Mastering long‐range orientation is of paramount importance to obtain a *proof‐of‐concept* (PoC) demonstration of nanoconfined ionic transport,^[^ ^8^ ^]^ going beyond the current *state‐of‐the‐art* performances of electrolytes. To achieve this ambition requires capitalizing on a range of intrinsic and extrinsic strategies.

As TILCs represent a functional (*i.e.*, ion conducting) sub‐class of TLCs, it is of crucial importance to precisely control their anchorage onto electrodes to enable homogeneous or homeotropic alignments of their columnar,^[^ ^43^, ^44^, ^45^, ^46^ ^]^ smectic^[^ ^47^, ^48^, ^49^, ^50^ ^]^, and cubic bicontinuous mesophases.^[^ ^51^, ^52^, ^53^ ^]^ This can be easily achieved by spontaneous alignment of TILCs onto well‐chosen electrode materials, *i.e.*, without using self‐assembling monolayer (SAM) functionalization strategies, or alternatively, when required in absence of spontaneous alignment, by various SAM modifications.^[^ ^69^ ^]^

Beyond the prerequisite of a perfectly controlled anchoring of TILCs onto electrodes, three main extrinsic strategies can be further implemented to propagate supramolecular self‐assemblies of prescribed mesomorphic morphologies up to filling the micron‐scale gap separating electrodes, *i.e.*, to create GB‐free TILC monodomains:

Strategy 1: Mechanical shearing is widely considered to be one of the most straightforward means to induce long‐range ordering in TLCs and TILCs. Indeed, it has proven to be a feasible and versatile approach for extending locally self‐assembled structures up to macroscopically aligned states, particularly in the case of TLCs^[^ ^70^, ^71^, ^72^ ^]^ and, at least once, for a TILC.^[^ ^44^ ^]^ Nevertheless, the experimental implementation of this method presents significant challenges, especially if coupled with Electrochemical Impedance Spectroscopy (EIS). In fact, technical difficulties, such as the desynchronization of the characteristic times, arise when attempting to achieve truly simultaneous in situ and *operando* access to mesoscopic and molecular/nanoscale information within the multiscale structure of TILCs.
Strategy 2: Applying an AC‐electric field offers a convenient and efficient alternative. The geometric dimensions of commercially available interdigitated electrodes or LC cells (gaps of ca. 2 to 50 µm) allow for straightforward implementation of AC‐electric field up to 5–10 kV·cm^−1^ (*i.e.*, 5–10 V·µm^−1^) using arbitrary wave generators. As reported in the literature, TILCs provide an intriguing “test bed”^[^ ^73^ ^]^ for addressing the roles of dimensionality and mosaicity. The application of AC‐electric could enable fine‐tuning of the confinement persistence^[^ ^74^ ^]^ through the long‐range orientation of mesophases.^[^ ^75^ ^]^ Nevertheless, it is worth mentioning here that, to date, only one group^[^ ^61^ ^]^ has reported the beneficial effects of applying an AC‐electric field onto TLCs doped with a lithium salt to tune ionic conductivity due to potential undisclosed technical difficulties that we have also encountered.
Strategy 3: Magnetic fields in the range 1–10 T are usually required, leading to accessibility and configuration challenges. The use of magnetic fields greater than 1 T complicates the implementation of this strategy.^[^ ^76^ ^]^ Additionally, applying an external magnetic field necessitates the use of non‐magnetic electrodes as a prerequisite. Nevertheless, some remarkable studies, notably by Prof. C.O. Osuji and co‐workers,^[^ ^77^, ^78^ ^]^ have provided definitive PoC demonstrations of long‐range oriented TILCs^[^ ^79^, ^80^ ^]^ using magnetic fields. This approach was finally successfully applied to obtain longer‐range ordered TILCs under magnetic field stimulus, and enabled simultaneous in situ and *operando* studies of their structure and transport properties.

Herein, we experimentally implement this latter approach to directly correlate variations in dynamic mosaicity with ion transport properties. We first describe the design, synthesis, and chemical characterization of imidazolium‐based anionic TILCs, followed by their supramolecular organization into a smectic mesophase, and finally their thermal behavior and ion transport properties.

## 2 Results and Discussion

### 2.1 Building TILCs by Design: Modular Synthesis and Chemical Tuning

**Figure 1:**
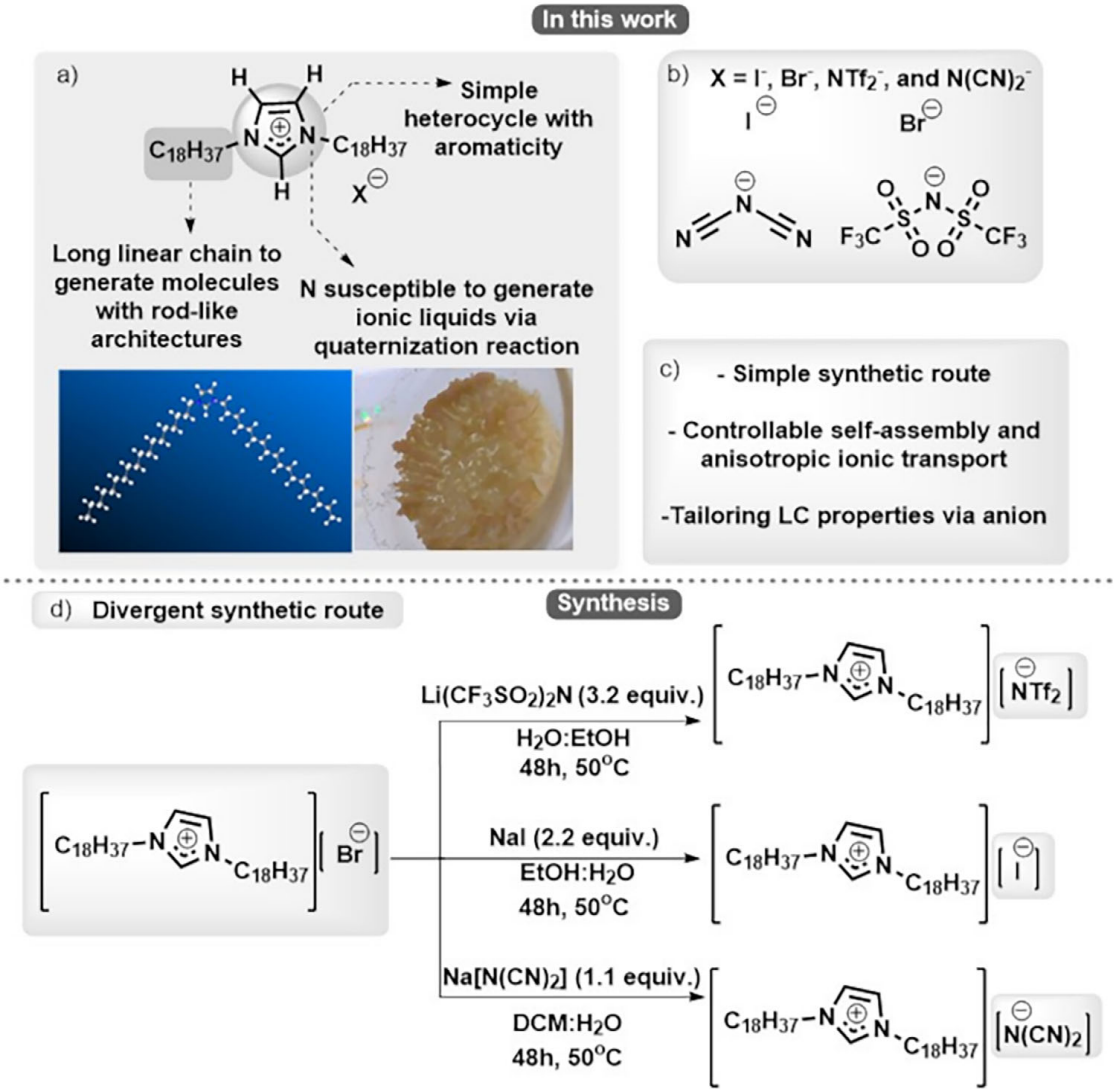
Molecular design and synthesis strategy for imidazolium‐based TILCs: **a)** Key structural elements of the imidazolium core promote rod‐like self‐assembly and ensure ionic functionality. **b)** Anions investigated in this study: Br^−^, I^−^, [NTf_2_]^−^, and [N(CN)_2_]^−^. **c)** Synthetic advantages of the modular platform: Broad anion compatibility and tunable mesophase behavior. **d)** Synthetic routes based on anion metathesis from a common imidazolium bromide precursor.

A few reviews have described and analyzed the rich mesomorphism of imidazolium‐based TILCs^[^ ^62^, ^81^ ^]^ into hexagonal columnar,^[^ ^82^, ^83^ ^]^ smectic,^[^ ^82^ ^]^ and cubic bicontinuous^[^ ^82^ ^]^ mesophases. L. Douce and co‐workers^[^ ^81^ ^]^ as well as C. Tschierske^[^ ^12^ ^]^ have established comprehensive molecular engineering design rules for insulating subparts (asymmetric vs. symmetric substitution with n‐alkyl chains and pro‐mesogenic 3,4 vs. 3,5 vs. 3,4,5 alkoxy‐substituted polycatenar peripheral groups), while other experts have emphasized the equally critical role of mesomorphism variations produced by cations (e.g., hydrophobic vs. hydrophilic nature, size, and shape) nature related to the ionically conducting sub‐parts of TILCs.^[^ ^14^, ^25^, ^40^ ^]^ Taking cross‐fertilizing's inspiration from this literature (see Figures S2 and S3, Supporting Information) to design and synthesize *tunable‐by‐design* imidazolium‐based (see Figure S4, Supporting Information) ionically conducting materials,^[^ ^14^, ^81^, ^84^ ^]^ synthetic variations along these two lines (see **Figure** **1**) were considered, while relying first **(i)** on easily accessible brominated synthons to generate [C_18_C_18_Im]^+^/Br^−^ and second **(ii)** on anion metathesis reactions to access [C_18_C_18_Im]^+^/I^−^, [C_18_C_18_Im]^+^/[N(CN)_2_]^−^ and [C_18_C_18_Im]^+^/[NTf_2_]^−^ analogues (see also the chemical rationale in the Part S2.2, Supporting Information). The synthesis strategy was developed following a retrosynthesis process (See Figures S5 and S6, Supporting Information). We started our synthesis by reacting I‐C_18_H_37_ and imidazole under basic conditions (NaH, 3 equiv.) at 30 ^°^C in MeCN to obtain C_18_‐Im (see Table S2, entry 9, and Figure S7, Supporting Information). Subsequently, the imidazole quaternization reaction provided a yield of 87% when carried out at 100 °C for 16 h in MeCN (see Table S3, entry 4, and Figure S8, Supporting Information).

Nuclear magnetic resonance (NMR)‐based techniques such as COSY, HSQC, and HMBC were used to fully elucidate the structure of the resulting [C_18_C_18_Im]^+^/Br^−^ salt. COSY showed that H_1_ and H_2_ do not couple with the aliphatic hydrogens. In addition, COSY revealed the formation of a spin system between H_3_ and H_4_, proving the presence of the long linear chain (see Table S5, Supporting Information). HMBC exhibited a strong correlation between the most de‐shielded carbon of the imidazole (located at *δ =*136.1 ppm) with the H_3_ and H_2_ hydrogens, confirming that the long linear chain was connected with the imidazole heterocycle (see Table S6, Supporting Information). Finally, the infrared (IR) spectrum showed the presence of a band between 725‐720 cm^−1^, indicative of methylene rocking vibration, typical of long linear chains (See Figures S17–S21, Supporting Information).

After optimizing the nucleophilic substitution reaction and ionic liquid synthesis, we observed that the greatest challenge of this synthetic route consisted in the ion exchange reactions to obtain [C_18_C_18_Im]^+^/[N(CN)_2_]^−^ and [C_18_C_18_Im]^+^/[NTf_2_]^−^ since ion‐exchange resins were not employed in this work. This factor led to greater difficulties in purifying the targeted ionic liquids (See Part S3.3, Supporting Information). The purification process of the anionic metathesis reaction is more efficient when using [C_18_C_18_Im]^+^/Br^−^ as a starting material instead of [C_18_C_18_Im]^+^/I^−^ (See General Procedure A of Supporting Information).

All obtained imidazolium salts were subjected to chemical characterizations using ^1^H, ^13^C, and ^19^F NMR spectroscopies. Due to the higher acidity of hydrogen in C_1_‐H_1_ of the imidazole ring, significant changes in the chemical shift were observed for [C_18_C_18_Im]^+^/Br^−^ and [C_18_C_18_Im]^+^//I^−^ salts (Δ*δ* = 0.25 ppm, where *δ*(C_1_‐H_1_) is 10.65 ppm for [Br]^−^ and 10.4 ppm for [I]^−^ as counterion, see Figure S9, Supporting Information). This effect was also observed in the ^13^C spectra (Δ*δ* = 1.15 ppm, where *δ*(C_1_‐H_1_) is 137.27 ppm for [Br]^−^ and 136.12 ppm for [I]^−^ (see Figure S10, Supporting Information). One explanation is the greater electronegativity of Br^−^ compared to I^−^. The smaller atomic radius of bromine causes: **(i)** a more significant effect in removing the electronic density of hydrogen C_1_‐H_1_; **(ii)** deshielding of the C_1_‐H_1_ hydrogen, and **(iii)** an increase in the chemical shift.

The chemical shift values for the [NTf_2_]^−^ and [N(CN)_2_]^−^ imidazole salts for the *δ*(C_1_‐H_1_) hydrogen were lower than for the halogen salt, an expected fact due to the resonance phenomenon.^[^ ^85^ ^]^ The quartet generated by the coupling between C‐F (*J_C‐F_* = 320 Hz) and the presence of the ^19^F signal at ‐78.95 ppm confirmed that an ion exchange reaction for [NTf_2_]^−^ took place, while the peculiar ^13^C NMR signal at 119.99 ppm proved that the [N(CN)_2_]^−^ anion was also successfully obtained, furthermore, [NTf_2_]^−^ and [N(CN)_2_]^−^ were detected in the negative mode of the HRMS analysis (see Figures S13–S16b, Supporting Information).

Due to the limitation of NMR in detecting inorganic impurities, HRMS analyses were performed to track and detect inorganic species as impurities. In all cases, the positive mode showed only the cation with a ratio (*m/z* = 573.6071) being the majority chemical species in the samples (see Figures S11,S12, and S16a, Supporting Information).

### 2.2 Revealing Mesophases: Texture, Thermodynamics, and Coarse‐Grained Insights

**Figure 2:**
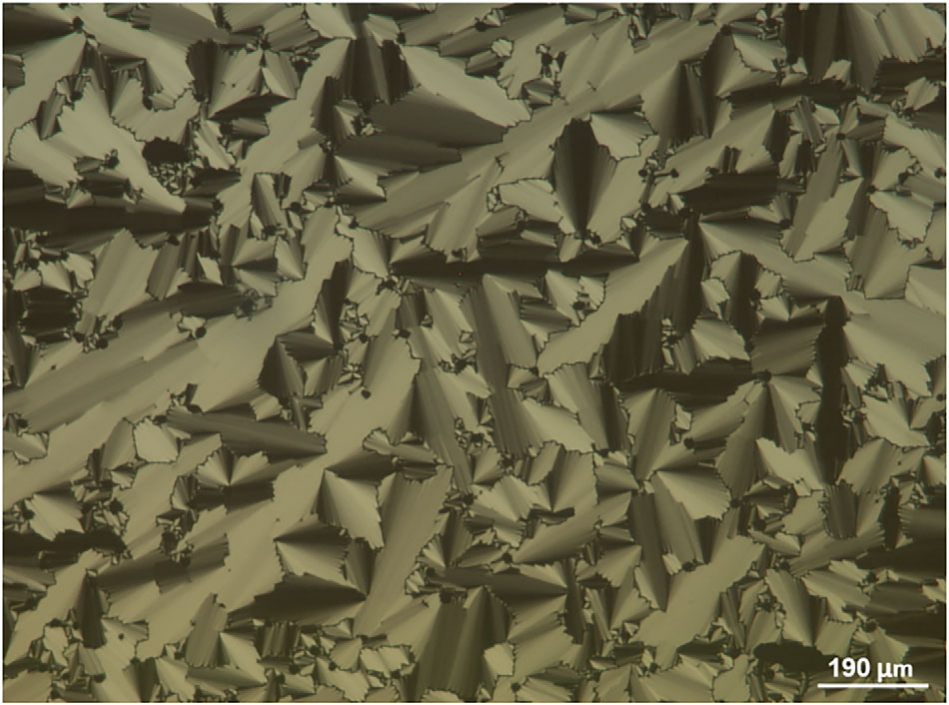
Polarized optical microscopy (POM) image of [C_18_C_18_Im]^+^/[N(CN)_2_]^−^ at 100 °C upon first cooling from the isotropic phase, showing the focal conic fan texture under homogeneous alignment. Scale bar: 190 µm. Similar textures were consistently observed across different samples and temperatures.

After ensuring their thermal stability by TGA (See Part S7, Supporting Information), the thermal and self‐assembling properties of the TILCs were also investigated by differential scanning calorimetry (DSC) and polarizing optical microscopy (POM), respectively. The POM images obtained at 100 °C during the first cooling scan of the TILCs from their isotropic state are shown in **Figures** **2** and S24 (Supporting Information). They reveal the appearance of representative birefringent textures with focal conic fans. This texture is characteristic of the presence of a lamellar organization, as observed for a smectic A mesophase (SmA). Indeed, the layers of a SmA mesophase bend or curl, forming fan/conical structures.

During the first cooling scan, the TILC with the largest anion (*e.g.*, [NTf_2_]^−^) exhibited a birefringent mosaic texture (See Figure S24, Supporting Information) at 57.4 °C characterized by a granular appearance under POM imaging, with a multitude of birefringent domains of different sizes and shapes separated by sharp boundaries. Although a mosaic texture is more likely related to a lamellar mesophase with long‐range intra‐lamellar correlation (*e.g.*, smectic B crystal or smectic E), it is difficult to draw a definitive conclusion on the nature of this mesophase from the observed texture. Due to this singular mesomorphism, this TILC is not associated with the comparison of the structural and transport behaviors.

**Figure 3:**
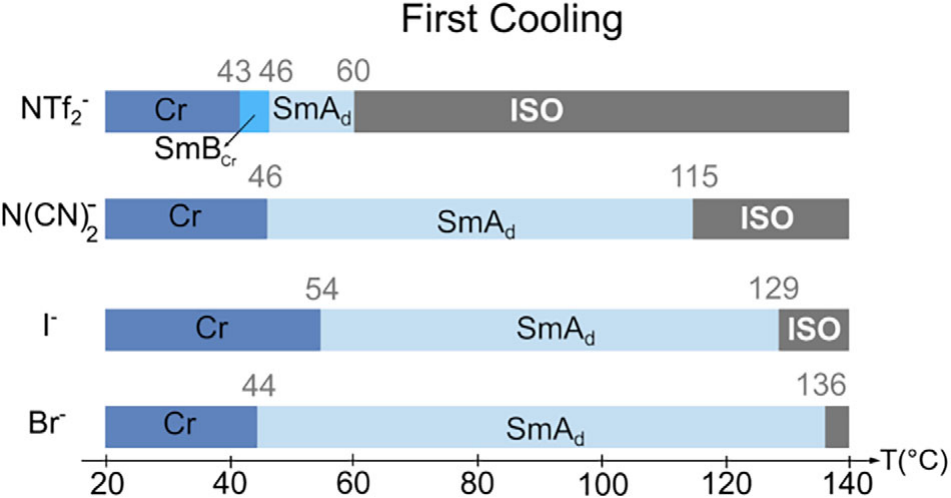
Phase transition behaviors of TILCs during the first cooling scan. Cr = crystalline phase, SmA_d_ = interdigitated smectic A, SmB_Cr_ = smectic B crystal, ISO = isotropic phase. The thermal behavior shown is representative of multiple measurements performed on independently prepared samples, using different instruments and protocols.

Temperature‐dependent POM detailed studies also allowed us to identify the thermal transitions corresponding to the ordering (following the melting point) and disordering (clearing point) of mesophases. The transition temperatures, enthalpies (See thermograms of Figure S23, Supporting Information) and phase transition assignments are summarized in the synoptic **Figure** **3**. The mesomorphic temperature ranges, in between the clearing and crystallization temperatures, decrease with the anion size, allowing the related TILCs to be studied in relevant temperature ranges. The halide‐based TILCs, [C_18_C_18_Im]^+^/Br^−^ and [C_18_C_18_Im]^+^/I^−^, present the highest clearing temperatures at 129 and 136 °C, respectively.

**Figure 4:**
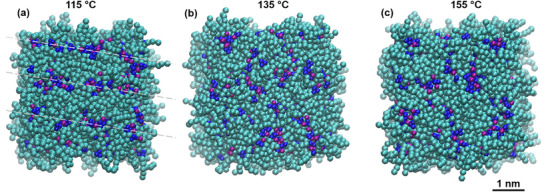
Snapshots from coarse‐grained simulations (1920 ion pairs and 26,880 particles) of [C_18_C_18_Im]^+^/Br^−^. Images correspond to: **a)** SmA_d_ mesophase at 115 °C, **b)** transition region at 135 °C, and **c)** isotropic phase at 155 °C. Bromide ions (purple) and imidazolium head groups (blue) form polar domains. SmA_d_ lamellar ordering is visible at 115 °C (*the oblique light grey dotted lines are only guidelines for eyes*), weakens at 135 °C, and disappears at 155 °C with no long‐range positional order. These snapshots are representative of equilibrated configurations from independent simulation runs under the same thermodynamic conditions.

Coarse‐grained simulations support these mesomorphic descriptions. While all‐atom simulations—where every atom is explicitly represented—are valuable for capturing fine‐scale interactions, they remain computationally prohibitive for exploring the time and length scales relevant to mesophase behavior. In contrast, coarse‐grained (CG) models accelerate simulations by 10^2^–10^3^ times by reducing the number of degrees of freedom. In the Martini 3 CG force field, each interaction site represents several atoms but retains chemical specificity through parameterization.^[^ ^86^, ^87^ ^]^ Using structural information from all‐atom simulations of [C_18_C_18_Im]^+^‐based ionic liquids, we defined the corresponding Martini bead types for related TILCs. Simulation protocols are detailed in Sections 13 and 14 of the Supporting Information. Corroborating experimental thermotropic transitions, the CG simulations yield molecular‐level snapshots of the structural evolution from smectic to isotropic phases (**Figure** **4**).

### 2.3 Structural Anisotropy Driving Directional Ion Transport

**Figure 5:**
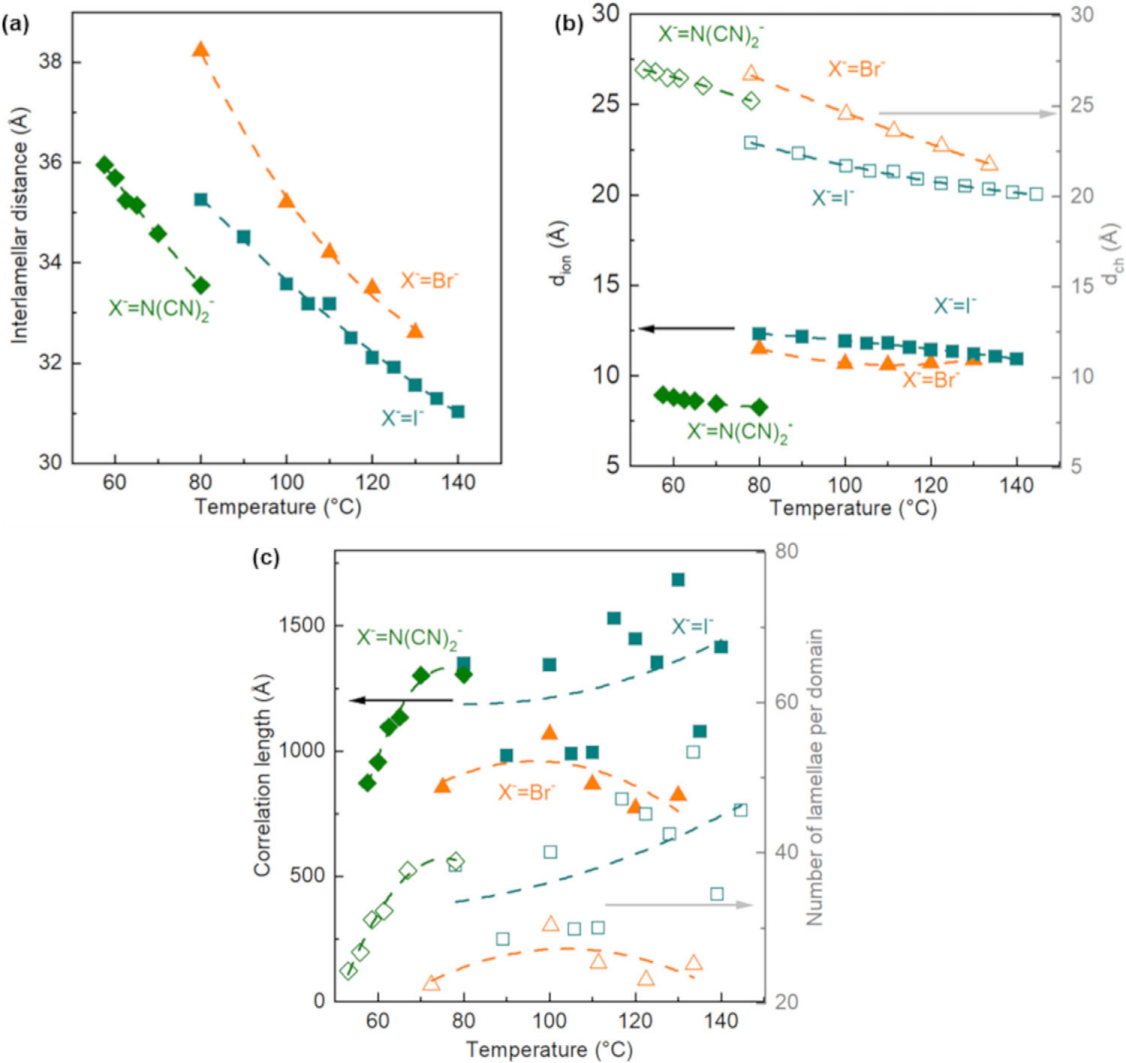
Evolution of **a)** interlamellar distances (*d*), **b)** thicknesses of ionic domains (*d_ion_*) and aliphatic (*d_ch_* = *d_001_*‐*d_ion_*) sublayers, and **c)** correlation lengths (*ξ*) and number of lamellae per domain *(ξ/d*) as a function of temperature during the first cooling for C_18_C_18_Im^+^/Br^−^, [C_18_C_18_Im]^+^/I^−^, and [C_18_C_18_Im]^+^/[N(CN)_2_]^−^. Dashed lines are included as visual guides. Each data point corresponds to a measurement performed on independently prepared samples under identical conditions. Data are shown as measured; no statistical tests were applied. The trends are representative of multiple consistent data series.

Describing and understanding the supramolecular structure of these mesophases, using temperature‐dependent small‐/wide‐angle X‐ray scattering (SWAXS), is crucial for probing and rationalizing the defect‐driven ion transport. All compounds show similar SWAXS 2D and 1D patterns (see Figures S25–S28, Supporting Information) with a first order Bragg reflection between 1.5 and 2 nm^−1^ corresponding to their (001) reflection with a few higher order Bragg reflections suggesting a lamellar organization with well‐correlated layers separated by sharp interfaces.^[^ ^88^, ^89^, ^90^ ^]^ The reflections were fitted with Gaussian functions to enable detailed structural analyses. Accordingly, the interlamellar distances (*d*) of the lamellar organizations of these mesophases, calculated from Bragg's law, as well as the correlation lengths (*ξ*) (see Sub‐section S10, Supporting Information), giving the average size of the dynamic domains, and the related numbers of lamellae per domain (*ξ/d*) are respectively shown in the **Figure** **5**, and respectively listed in the Tables S8–S10 (Supporting Information). Variations in the electronic densities along the director, the vector locally orthogonal to the lamellae plane, are also well‐described by atomistic simulations (see Figure S37, Supporting Information).

These interlamellar distances decrease with increasing anion size (see **Figure** 5a). Furthermore, contrary to what is generally observed in systems without ionic interactions (such as TLCs), the interlamellar distances decrease with increasing temperature (see Figure 5a). This phenomenon results from the balance between van der Waals forces (electrostatic interactions) and ionic/coulombic interactions since alternating ionophilic and ionophobic sub‐layers, based on molten *n*‐alkyl chains (see Sub‐section S10, Supporting Information), form the lamellae. At higher temperatures, short‐range interactions such as van der Waals interactions are weakened by thermal agitation, allowing longer‐range ionic interactions to develop, bringing mesogens closer together. However, as the temperature decreases, the overall thermal agitation of the system decreases, favoring the densification of short‐range interactions, frustrating ionic interactions, and gradually moving the mesogens further apart. In this way, the observed variations in interlamellar distances are governed by a delicate balance between short‐range (van der Waals) and long‐range (ionic) interactions, modulated by thermal fluctuations induced by changes in temperature.

Consequently, the decrease in the interlamellar distances with temperature, in the range between 3.1 and 3.8 nm (see Figure 5a), is mainly due to the increase in n‐alkyl chain interdigitation (see Sub‐section S11, Supporting Information). As shown in Figure 5b, the decrease in thickness (*d_ion_*, see calculation in Sub‐section S13, Supporting Information) of the ionophilic sub‐layers represents ca. 30% of this total decrease, while the reduction in thickness (*d_ch_*) of the ionophobic sub‐layers based on n‐alkyl chains represents 70% in the range between 2.2 and 2.7 nm thick. These thicknesses, which can be modulated by anion nature and temperature, allow model tunable nanoconfinement within ionophilic sub‐layers. These thin, structured 2D‐slabs provide nanoconfinement along two dimensions in the plane transverse to the local director. Using bromide/iodide and dicyanamide ions, 2D nanoconfined ion layers of ca. 1.2 and 0.7 nm are formed, respectively.

The correlation lengths (see Figure 5c), estimated from peak widths, increase upon heating and slowly decrease near the clearing temperatures. This observation, which could be considered a breach of classical/equilibrium thermodynamics, seems to be consistent with non‐equilibrium thermodynamics. Indeed, as already mentioned for a homeotropic smectic‐ordered electrolytic complex,^[^ ^91^ ^]^ the packing frustrations of the lamellar order can be mitigated **(i)** by the formation of polydomains and/or **(ii)**, as in the present case, also by increased long‐range order via facilitated fluidity and the ability of molecular rearrangement upon heating. It is worth noting here the large average size of the dynamic domains showing correlation lengths up to 150 nm, *i.e.*, 70 lamellae.

This long‐range structural order is locally irreversible from a statistical mechanics perspective, as returning to a given configuration at a fixed temperature requires passing through intermediate states, such as the isotropic phase during clearing. This irreversibility contributes to entropy generation. Structural parameters (interlamellar distance, correlation length), morphological features (mesoscopic domain organization), and transport data all suggest that entropy variations, which are — influenced by both anion type and temperature — central to the emergence of the observed mesoscopic phenomena.

The interplay between structural organization and entropy dissipation underlies the system's unique transport behavior. In this context, dynamic mosaicity and its associated homophasic boundaries play a critical role in enabling directionally confined ion transport,^[^ ^92^ ^]^ both in general and within this model family in particular. This connection is explicitly addressed in the present work to provide a rational framework for interpreting ion mobility.

Although the model TILCs investigated in this study are thermotropic in nature, they share mechanistic similarities with lyotropic ionic liquid crystals and biological membrane systems, where anisotropic ion transport occurs along organized polar interfaces. The primary differences lie in the molecular origin of ordering and the nature of the external stimuli that drive phase transitions. Nevertheless, the fundamental principles of confinement‐driven ionic conductivity and interfacial alignment are common to all three systems

**Figure 6:**
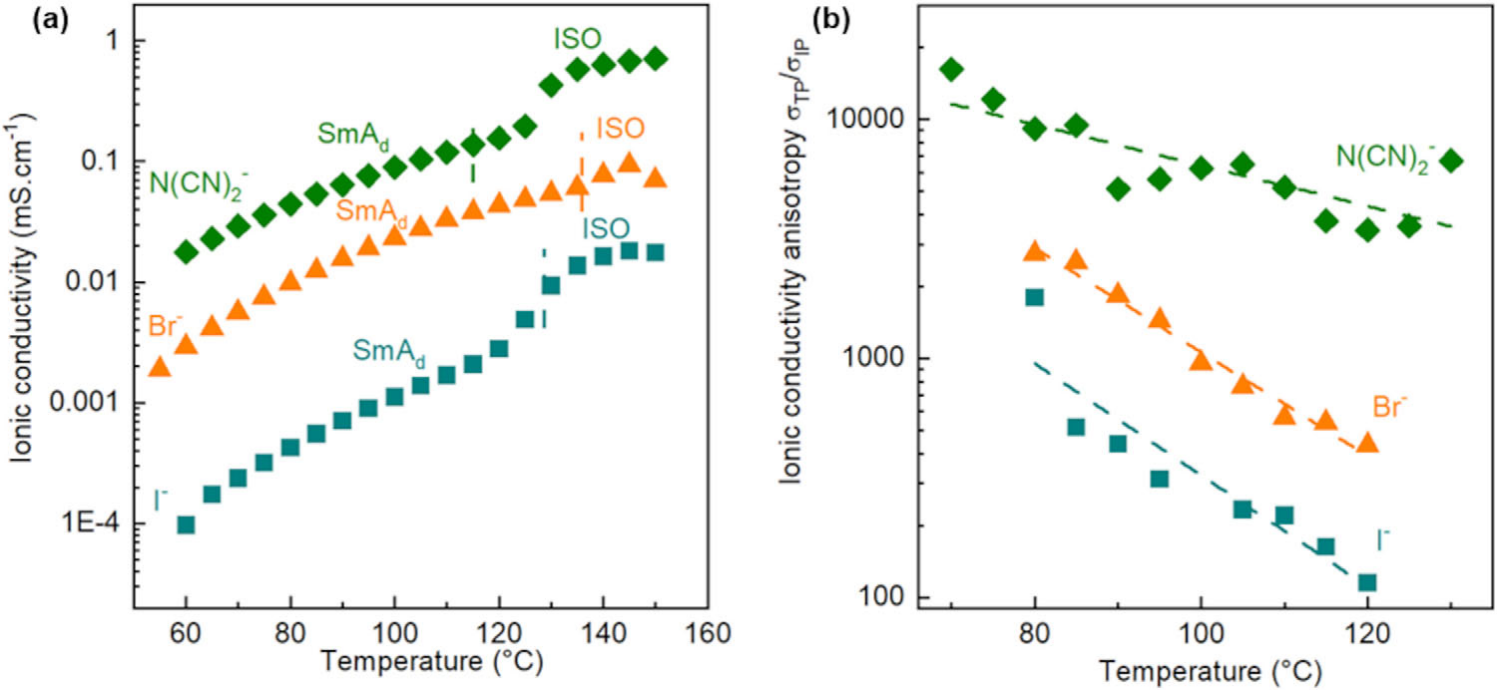
**a)** Through‐plane (TP) ionic conductivity and **b)** anisotropy ratio (*σ_TP_/σ_IP_*) as a function of temperature during the first cooling cycle for [C_18_C_18_Im]^+^/Br^−^, [C_18_C_18_Im]^+^/I^−^, and [C_18_C_18_Im]^+^/[N(CN)_2_]^−^. Dashed lines are provided as visual guides. Experimental uncertainties are estimated at ≈±20% based on reproducibility across independently prepared samples and measurement series. No statistical hypothesis testing was applied.

The ionic transport properties were studied both within and through the plane (in‐plane IP and through‐plane TP configurations) of their lamellar structures by electrochemical impedance spectroscopy (see Sub‐section S15, Supporting Information). In the considered mesomorphic temperature range, the TP‐ionic conductivity (*σ*_*TP*_) of [C_18_C_18_Im]^+^/[N(CN)_2_]^−^ (0.15 mS·cm^−1^) is about one and two orders of magnitude higher when compared to the ones of [C_18_C_18_Im]^+^/Br^−^ and [C_18_C_18_Im]^+^/I^−^, just before their clearing temperatures (see **Figure** **6**a). The results highlight not only the role of the ionic radius, but also that of the shapes of the counter‐ion on the nanoconfinement of charge carriers (see Sub‐section S12, Supporting Information) and their ionic transport. Notably, the non‐spherical and V‐shaped structure imposes significant constraints on the degrees of freedom of movement of dicyanamide anions, which counterintuitively allows them to diffuse more rapidly than the Br^−^ and I^−^ anions. This situation can be rationalized by a more directed movement of the dicyanamide anions—*i.e.*, a greater ionic conductivity anisotropy *σ_TP_/σ_IP_* (see Figure 6b)—as they are forced to follow the least energetic pathway between two ion‐blocking electrodes. Thus, this mean‐free path underlies the correlations between the ion transport and the surrounding structure and can be defined in terms of the energetic efficiency of the carrier diffusion, which is related to the rate of entropy dissipation associated with structuration as temperature increases.

**Figure 7:**
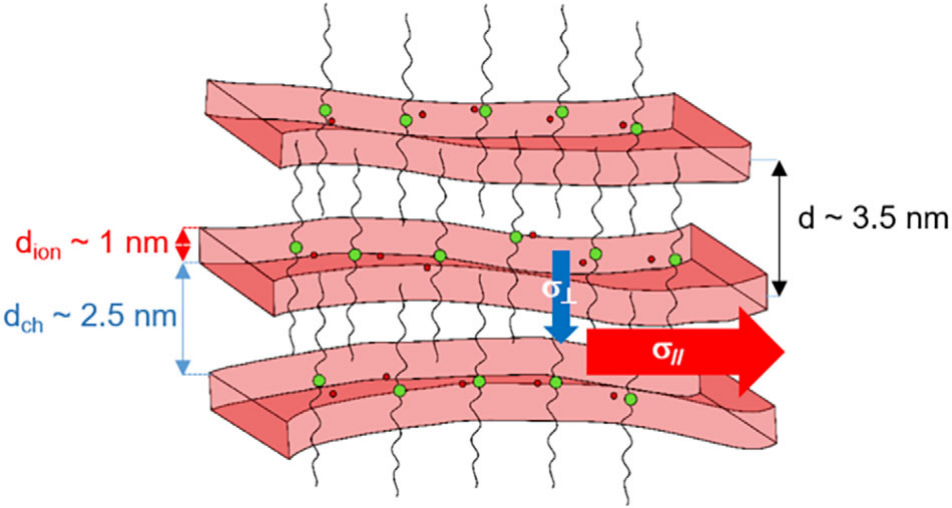
Synoptic illustration of efficient quasi‐2D anion transport within lamellar TILCs, highlighting the combined effects of structural anisotropy and nanoconfinement.

This mesoscopic description refers to the first elementary step of the dissociation (ionicity^[^ ^93^ ^]^)/local diffusion mechanism enhanced by the nanoscale confinement,^[^ ^94^ ^]^ essentially in the ionophilic sub‐layers, which occurs concomitantly with the in‐ and through‐plane vehicular diffusion of ion pairs.^[^ ^95^ ^]^ Both sub‐mechanisms result in an anionic transference number of ≈0.8, consistent with the directed nanoconfinement observed in coarse‐grained simulations of anionic and cationic self‐diffusion (see Sub‐section S14, Supporting Information). This unprecedented anisotropy, ranging from 10^2^ up to more than 10^4^ for [C_18_C_18_Im]^+^/[N(CN)_2_]^−^ at 70 °C, is then the result of these two main driving basic mechanisms, **(** **i)** intercationic distances favorable to an efficient dissociation and local diffusion essentially in the ionophilic sub‐domains, and **(ii)** a vehicular transport of the ion pairs essentially along the layers (See Figure S48, Supporting Information). Both basic mechanisms lead to a quasi‐2D anionic transport. Note that this anisotropy of transport is not optimal or ultimate since the homogeneous alignment onto the metallic electrodes (gold and platinum for TP and IP measurements, respectively) has not been fully optimized. This interpretation of anion transport mechanisms within the ionic/ionophilic sublayers is schematically illustrated in **Figure** **7**.

### 2.4 Mosaicity‐Governed Directional Ion Transport Probed by Magnetic Alignment

Even if the mosaicity slightly increases and the nanoconfinement becomes more critical with temperature, their effect on the increase in ionic conductivity is difficult to disentangle from the Brownian contribution, especially from a quantitative point of view. The energetic efficiency of the diffusion can be described by the mesoscopic tortuosity (*T*), which is the dimensionality (*D*) composed with the mosaicity (*M*) (*T* = *D* *M*). For a given dimensionality, 2D in the present case, this non‐commutative function becomes mainly dependent on the mosaicity. Only a direct correlation can disentangle its impact on the ionic conductivity, which is the weighted average, with the related carriers, mainly anions, volume concentrations of the main mobilities inside the domains, and in the homophasic defects.

**Figure 8:**
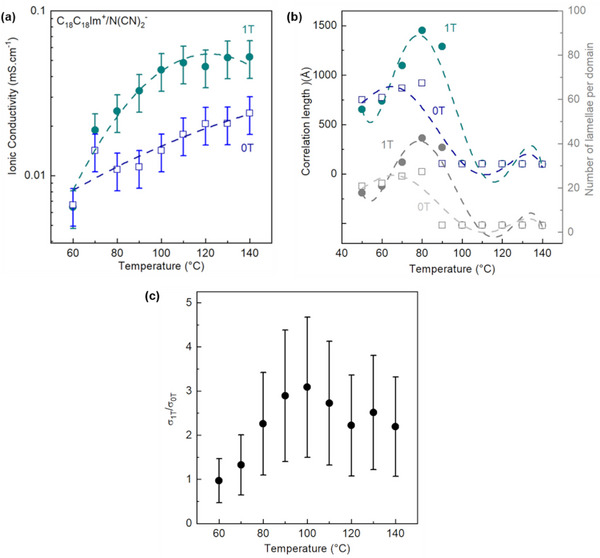
**a)** Through‐plane (TP) ionic conductivity (*σ_TP_*), **b)** correlation lengths (*ξ*) and average number of lamellae per domain (*ξ/d*) derived from azimuthally integrated 2D SWAXS patterns, and **c)** conductivity ratio (*σ_1T_/σ_0T_*) as a function of temperature of [C_18_C_18_Im]^+^/[N(CN)_2_]^−^ during the first (*B* = 1 T) and second (*B* = 0 T) cooling cycles. Dashed lines correspond to second‐order polynomial fits, shown as visual guides. Each data point corresponds to measurements from independently prepared samples. Experimental uncertainties are estimated at ±20%, based on reproducibility across multiple series. No statistical hypothesis testing was applied.

For this purpose, a dedicated setup was designed and built to carry out in situ and *operando* SWAXS experiments (see Sub‐section S10, Supporting Information) in order to monitor the variation of both the structure and ionic conductivity with temperature while allowing the application of a magnetic field of up to 1 Tesla (T). This represents a unique approach to directly correlate the effects of the long‐range organization, as probed by variations in mosaicity, with ionic conductivity. As expected, the results show a direct influence of the application of the magnetic field on ionic transport (**Figure** **8**a). The maximum increase in domain size and number of lamellae (Figure 8b) within the plane of the liquid crystal cell by ca. 150% at 80 °C is associated with a maximum increase in conductivity values of ca. 300% (from 15 to 45 µS·cm^−1^) at 100 °C (Figure 8c) induced by the application of a 1 T magnetic field stimulus. Unlike inorganic ceramics, where the temperature of maximum grain size increases coincides with that of maximum conductivity increase, the TILC system exhibits a temperature offset. This phenomenon reflects the complexity of the previously described ionic transport processes in TILC materials. It is caused by the presence of two different characteristic timescales associated with the domain reorganization and charge carrier diffusion. As a result, the Ostwald ripening appears to be accelerated and enhanced. This thermally activated growth of larger ionic domains, occurring at the expense of smaller ones, is linked to entropy dissipation as discussed in the antepenultimate paragraph. The application of a 1 T magnetic field induces larger mesomorphic dynamic domain sizes (and number of lamellae per domain) associated with an increase in long‐range order, a decrease in disorder and, most importantly, a reduction in the number of homophasic interfaces (which can penalize ion transport) leading to a natural (expected) increase in ionic conductivity values. To the best of our knowledge, this is the first direct experimental evidence of the interplay between defect responses in a model liquid crystalline electrolyte and their manipulation by an external magnetic field.

## 3 Conclusion

In summary, this work identifies dynamic mosaicity as a central structural parameter governing anisotropic ion transport in self‐assembled soft materials. We establish thermotropic ionic liquid crystals (TILCs) as a dimensionally controlled and tunable platform to probe and engineer confined ion transport. By integrating *operando* structural and electrochemical analyses, we provide direct experimental evidence of a coupling between supramolecular ordering and ion conductivity, modulated by mosaicity. Magnetic field‐induced alignment of smectic domains increases domain size and enhances ionic conductivity threefold, positioning mosaicity as a functional design lever within structure–property relationships.

Beyond a specific material system, these findings offer a generalizable framework for understanding and controlling ionic mobility through supramolecular order. The TILC platform enables quantitative access to confined transport regimes and connects stimuli‐responsive molecular assemblies with programmable electrolyte functionality. Altogether, this work lays the foundation for the rational design of adaptive soft‐matter systems with targeted ion transport, opening new avenues in energy storage and conversion, ionotronics, and bioinspired ionic devices.

## Conflict of Interest

The authors declare no conflict of interest.

## Author Contributions

H.P. and C.Y.O.‐J. contributed equally to this work. H. P. contributed to the investigation, methodology, resources, software, validation, visualization, and the original draft writing. C.Y.O.‐J. was involved in investigation, methodology, resources, validation, visualization, original draft writing, as well as reviewing and editing. M.S.S. contributed through formal analysis, investigation, methodology, resources, validation, and writing – review and editing. M.Mi. supported the work through formal analysis, methodology, software, supervision, validation, and writing – review and editing. I. M. was responsible for formal analysis, investigation, methodology, resources, software, supervision, and writing – review and editing. G.C. contributed with formal analysis, investigation, methodology, resources, and software. J.D.‐R. was involved in funding acquisition and validation. J.J. supported funding acquisition, software, supervision, validation, and writing – review and editing. A.P. contributed to funding acquisition, methodology, software, supervision, validation, and writing – review and editing. S. L. provided funding acquisition, resources, supervision, validation, and writing – review and editing. P.R. led the conceptualization, funding acquisition, investigation, methodology, resources, supervision, validation, and both original draft and review & editing of the manuscript. Finally, M.M. was responsible for conceptualization, formal analysis, funding acquisition, investigation, methodology, project administration, resources, supervision, validation, visualization, and writing – both original draft and review & editing.

## Supporting information



Supporting Information

## Data Availability

The data that support the findings of this study are available from the corresponding author upon reasonable request.
