# Supplementary material for: Dynamic Mosaicity Modulates Ion Transport in Stimuli‐Responsive Liquid Crystal Electrolytes
Source: Adv Sci (Weinh). 2025 Aug 11;12(41):e10610. doi: 10.1002/advs.202510610 (PMC12591113; doi:10.1002/advs.202510610)
Supplement: Supplementary file 1 — Supporting Information [file ADVS-12-e10610-s001.docx]

Supporting Information

**Dynamic Mosaicity Modulates Ion Transport in Stimuli-Responsive Liquid Crystal Electrolytes**

*Hélène Pung^†^, Celso Yassuo Okada-Junior^†^, Mirella Simões Santos, Marta Mirolo, Isabelle Morfin, Gilbert Chahine, Jannick Duchet-Rumeau, Johan Jacquemin, Agilio Padua, Sébastien Livi, Patrice Rannou*, Manuel Maréchal**

* Corresponding authors.

E-mail addresses:

Dr. Manuel Maréchal (manuel.marechal@univ-grenoble-alpes.fr).

Dr. Patrice Rannou ([patrice.rannou@grenoble-inp.fr](file:///D:\ANR%20CITADEL\Article%201\Soumission%20Materials%20Today\patrice.rannou@grenoble-inp.fr)).

^†^ These co-authors equally contributed.

**Table of Contents**

[**1. Materials and Methods** 2](#_Toc157156401)

[**2. Fundamental aspects and liquid crystal design** 2](#_Toc157156402)

[**2.1. Definition of liquid crystal and general aspects** 2](#_Toc157156403)

[**2.2. Chemical rationale of the imidazolium-based TILCs** 4](#_Toc157156404)

[**2.3. General design and architecture of liquid crystals** 5](#_Toc157156404)

[**3. Synthesis and optimisation of liquid crystals** 6](#_Toc157156405)

[**3.1. Retrosynthetic analysis** 6](#_Toc157156406)

[**3.2. Optimized synthetic route overview**](#_Toc157156407) 7

[**3.3 General procedures**](#_Toc157156408) 9

[**4. NMR characterisation** 1](#_Toc157156409)3

**4.1 NMR spectra comparison**………………………..………………………………………..19

[**4.2 Bidimensional NMR** 2](#_Toc157156410)1

[**5. Electrospray Ionization Mass Spectrometry (MS-ESI)** 2](#_Toc157156411)4

[**6. Infrared spectroscopic studies** 2](#_Toc157156412)8

[**7. Thermogravimetric Analysis (TGA) summary** 3](#_Toc157156413)3

[**8. Differential Scanning Calorimetry (DSC)** 3](#_Toc157156414)6

[**9. Polarised Optical Microscopy (POM)** 3](#_Toc157156415)7

[**10. Small- and Wide-Angle X-ray Scatterings (SWAXS)** 3](#_Toc157156416)9

[**11. Level of *n*-alkyl chain interdigitation** 4](#_Toc157156417)7

[**12. Nanoconfinement as a function of the nature of the anion** 4](#_Toc157156418)8

[**13. Atomistic simulations** 4](#_Toc157156419)9

# **14. Coarse-grained force fields**…………………………………………………………….….56

# **15. Ion transport properties as probed by Electrochemical Impedance Spectroscopy (EIS)**…………………………………………………………………………………………….57

**16. Statistical Analysis**…………………….…………………………………………………...63

**1. Materials and Methods**

All reagents were purchased from Merck/Sigma-Aldrich or TCI and were used without further purification. Solvents (including anhydrous ones) were purchased from Carlo Erba and were used as received. NMR spectra were recorded on Bruker Avance III 400 MHz or 500 MHz spectrometers. Samples were prepared by dissolution in an appropriate deuterated solvent (CDCl_3_, CD_3_CN or DMSO-d_6_). The chemical shifts (δ) are expressed in ppm with the residual solvent peak or TMS as the internal standard. Coupling constants are indicated in Hz. Abbreviations for signal coupling are as follows: s= singlet; d=doublet; dd=doublet of doublets; t= triplet; q= quartet; quin= quintet; m= multiplet; br= broad signal. Additional 2D NMR experiments (COSY, HSQC, HMBC) were also performed using the same spectrometers. ^13^C HR-MAS solid-state NMR spectroscopy analysis was performed using a Bruker Avance II spectrometer (400 MHz), equipped with a 4 mm rotor ^1^H-^13^C HR-MAS probe with z-gradient coil at a 5 kHz rotation speed and at 25 °C. High resolution mass spectra HRMS were obtained by Electrospray Ionisation (ESI) on a Micromass-Waters Q-TOF Ultima Global. Fourier transform infrared spectroscopy (FT-IR) spectra were recorded on a Nicolet Magna 550 spectrometer at room temperature (25 °C) with a Specac/Golden Gate ATR accessory. 32 scans were collected for each FT-IR spectrum, with a spectral resolution of 4 cm^-1^ from 4000 to 425 cm^-1^. Variable temperature FT-IR spectroscopic studies were run under an ATR configuration (Specac/Golden Gate) using a Perkin Elmer Paragon 500 spectrometer with a spectral resolution of 4 cm^-1^ over the 4000-600 cm^-1^ wavenumber domain. Thin Layer Chromatography (TLC) analyses were realized on pre-coated aluminum plates of silica gel 60 F-254 (Merck). Flash chromatography was performed on a silica gel column (Merck silica gel, 40-63 mm) using air pressure. Thermogravimetric analyses (TGA) were performed on a TA instrument Q500 thermogravimetric analyzer. The samples were heated from 30 to 700 °C at a rate of 10 K·min^-1^ under a nitrogen flow. Differential scanning calorimetry (DSC) analyses were performed on a Linseis Chip DSC-10 in the dynamic mode at a rate of 10 K·min^-1^ from 25 to 150 °C. The DSC was calibrated with indium (T_f_ = 156.6 °C). Approximately 5-10 mg of TILCs were used per analysis.

# **2. Fundamental aspects and liquid crystal design**

**2.1. Definition of liquid crystal and general aspects**

Liquid crystals are materials offering simultaneously features of a solid (organisation due to the orientational order) and a liquid (mobility due to the positional disorder of the molecules).^[[1]](#footnote-1)^ From a (macro)molecular point of view, a liquid crystalline mesophase is obtained when a mesogen is defined by a favourable (frequently but not mandatorily anisotropic) chemical structure, e g., with long, ellipsoidal, disk-like shape sub-parts chemically incompatible but linked together. There are several dynamic self-assemblies (*i.e.,* mesophases) under which liquid crystalline (macro)molecules can self-organize: Nematic (*N*), Smectic (*Sm)*, columnar (*Col*), cubic bicontinuous (*Cub_bi_*), and cholesteric (Chiral nematic: *Nem**) mesophases. It should be noted that their physical and optical properties change accordingly to their (macro)molecular architectures (**Figure S1**).

**Figure S1.** Overview of states of matter.^[[2]](#footnote-2)^

**2.2. Chemical rationale of the imidazolium-based TILCs**

**Common Imidazolium cation**: The imidazolium cation [C_18_C_18_Im]^+^ is a heterocyclic organic compound that is easily functionalised at the N_(1)_ and N_(3)_ positions (**Figure** **S2**). The hydrogen atom attached to the C_(2)_ carbon atom is strongly acidic because the C_(2)_ atom has a partial negative charge (*due to the electron deficit of the positively charged nitrogen atoms*). This specificity allows it to interact with the counter ions.

**Figure S2.** Structural formula of the imidazolium cation [C_18_C_18_Im]^+^ and identification of the positions of its atoms on the heterocycle.

**Anions**: Br^-^, I^-^, [N(CN)_2_]⁻ and [NTf₂]⁻ anions can be classified according to their size, shape, and properties (**Table S1**). The ionic radius of the anions increases in the following order: Br^-^ (1.96 Å) < I^-^ (2.2 Å) < [N(CN)_2_]⁻ (5.2 Å) < [NTf₂]⁻(7.5 Å). The values were obtained using TMoleX (Turbomole, V7.5, Dassault Systems, BIOVA, 2021) and COSMOConfX (BIOVA, 2021) programs, applying the DFT/BP86/def-TZVP level of theory.

**Table S1.** Table showing the sizes, shapes, and notable properties of Br^-^, I^-^, [N(CN)_2_]⁻, and [NTf₂]⁻ anions.

| Anion | Ion radius / Å | Shape | Notable properties |
| --- | --- | --- | --- |
| Br^-^ | 1.96 | Spherical | Reactive |
| I^-^ | 2.2 | Spherical | Reactive, greater polarizability |
| [N(CN)_2_]⁻ | 5.2 | V-shaped configuration | Known for its ability to form hydrogen bonds with cations, and can be used as a halogen-free coordination anion |
| [NTf₂]⁻ | 7.5 | Trigonal with a C_3_v symmetry | Thermostable, often used in battery electrolytes |

**[N(CN)_2_]⁻** **anion**. The [N(CN)_2_]⁻ anion has an ovoid shape with a V configuration and a larger volume than the spherical Br^-^ and I^-^ anions, imparting its special properties such as low viscosity and eliminating the need for halide anions. Mudring *et al.* were the first to study the mesomorphic properties of a TILC with dicyanamide as the anion (1,3-bis(*n*-dodecyl)-1,2,3-thiazolium dicyanamide)^[[3]](#footnote-3)^, forming a smectic C mesophase between 82 and 102 °C.

**[NTf₂]⁻ anion**. The bis(trifluoromethylsulfonyl)imide anion, also known as [NTf₂]⁻, is an anion commonly used in battery and supercapacitor electrolytes due to its solubility, electrochemical stability, and thermostability properties. There are two *cis* and *trans* conformers^[[4]](#footnote-4)^ of [NTf₂]⁻, which differ in the relative position of the two trifluoromethylsulfonyl (TFS) groups around the central nitrogen atom. The *cis* conformer has two TFS groups facing the same side of the molecule, whereas the *trans* conformer has two TFS groups facing opposite sides. As a result of this structural difference, the two conformers have different physicochemical properties. Theoretical and experimental studies have shown that the *trans* conformer is more stable than the *cis* conformer. It is generally the majority conformer in [NTf₂]⁻ solutions. The *trans* conformer is also more polarisable and soluble than its *cis* counterpart, giving it improved ion transport properties.

**2.3. General design and architecture of liquid crystals**

**Figure S3.** A few imidazolium-based and bis-imidazolium TILCs were selected from the literature.^[[5]](#footnote-5)^

**Figure S4.** Imidazolium-based TILCs were designed and synthesized in this study**.**

**3. Synthesis and optimisation of liquid crystals**

# **3.1. Retrosynthetic analysis**

**Figure S5.** Molecules designed.^[[6]](#footnote-6)^

# **3.2. Optimized synthetic route overview**

**Figure S6.** Overview of reactions employing imidazole for TILC synthesis.^[[7]](#footnote-7)^

**Table S2**. *S_N_*2 reaction of nitrogen-like imidazole.

| *entry* | *X (equiv.)* | *solvent (Y* mol·L^-1^*)* | *yield (%)^[a]^* |
| --- | --- | --- | --- |
| 1 | 1.2 | DMF (0.1) | 35 |
| 2 | 1.2 | THF (0.1) | 15 |
| 3 | 1.2 | Petroleum ether (0.1) | 20 |
| 4 | 1.2 | MeCN (0.1) | 80 |
| 5 | 1.2 | 1,4-dioxane (0.1) | 0 |
| 6 | 1.2 | Toluene (0.1) | 0 |
| 7 | 1.2 | DMF (0.1) | 35 |
| 8 | 2 | MeCN (0.1) | 89 |
| 9 | **3** | **MeCN (0.1)^[b]^** | **99** |
| 10 | 4 | MeCN (0.1) | 99 |

***[a]*** *Estimated based on the ^1^H NMR crude employing 1,3,5-trimethoxybenzene as internal reference****. [b]*** *The solvent does not have to be anhydrous.*

**Reaction details:**

**Figure S7.** General information of the optimized reaction.

**Table S3**. Quaternisation optimisation

| *entry* | *solvent (Y, mol·L^-1^)* | *yield (%)^[a]^* |
| --- | --- | --- |
| 1 | DCE (0.1) | 35 |
| 2 | Toluene (0.1) | 75 |
| 3 | 1,4-dioxane (0.1) | 76 |
| 4 | MeCN (0.1)^b^ | 87 |

***[a]*** *Estimated based on the ^1^H NMR spectroscopic analysis of the crude product employing 1,3,5-trimethoxybenzene as the internal reference****. [b]*** *The solvent does not have to be anhydrous.*

**Reaction details:**

**Figure S8.** General information about the optimized reaction.

# **3.3 General procedures**

***1-octadecyl-1H-imidazole***

Adapted from a procedure by Zhao and co-workers*^[[8]](#footnote-8)^*; as well as Soares *et al*.^[[9]](#footnote-9)^ In a solution of 1H-imidazole (680 mg; 10 mmol; 1 equiv.) in MeCN (100 mL, 0.1 mol·L^-1^), 60% NaH (720 mg, 30 mmol; 3 equiv.) was slowly added (See Table S1). After bubbling off, 1-iodooctadecane (4.18 g; 11 mmol; 1.1 equiv.) was added. The reaction was allowed to warm to room temperature (29 ^o^C) for 12 hours. The mixture was diluted with AcOEt and washed with a brine aqueous solution (saturated solution of NaCl). Column chromatography (SiO_2_) was employed to purify the product using a gradient (9:1, 7:3, 5:5, 2:8 AcOEt: MeCN). The desired product was obtained as a white solid (3.03 g; 9.5 mmol; 95% yield) (See Figure S3).

**TLC:** *R_f_* = 0.5 (2:8 AcOEt: MeCN, *molybdate stain*).

**^1^H NMR (400 MHz, CDCl_3_) δ_H_:** 8.27 (s, 1H),7.1 (s, 1H), 7.0 (s, 1H), 4.1 (t, *J* = 7.0Hz, 2H), 1.82 – 1.79 (m, 2H), 1.29 – 1.23 (m, 30H), 0.86 (t, *J* = 7.0Hz, 3H)ppm. [*See spectrum*](#nmrim)

**^13^C NMR (100 MHz, CDCl_3_) δ_C_:** 136.5, 125.6, 119.5, 48.0, 31.8, 30.8, 29.6, 29.58, 29.57, 29.5, 29.4, 29.3, 29.2, 28.9, 26.4, 22.6, 14.0ppm. [*See spectrum*](#nmrimC)

**IR (ATR, cm^-1^):** 3494, 3420, 3113, 2912, 2849, 1614, 1514, 1458, 1364, 1289, 1226, 1113, 1082, 1038, 913, 837,813, 725, 718, 649.

**1,3-dioctadecyl-1H-3|4-imidazol-1-ium iodine ([C_18_C_18_Im]^+^/I^-^)**

Adapted from a procedure by Livi *et al*.^[[10]](#footnote-10)^ In a solution of *1-octadecyl-1H-imidazole* (3.2 g; 10 mmol; 1 equiv.) in MeCN (100 mL, 0.1 mol·L^-1^), *1*-iodooctadecane (4.18 g; 11 mmol; 1.1 equiv.) was added (See Table S2).The reaction was allowed to warm to 100 ^o^C for 16 hours. The mixture was precipitated in diethyl ether. The desired product was obtained as a brown solid (5.04 g; 7.19 mmol; *72% yield*) (See Figure S3)—alternatively, a general procedure A was used to obtain this compound. A mixture of 1,3-dioctadecyl-1H-3**|**4-imidazol-1-ium bromine (214 mg; 0.3 mmol; 1 equiv.) in EtOH (10 mL, 0.03 mol·L^-1^) was added to sodium iodide (NaI) (99 mg; 0.66 mmol; 2.2 equiv.) and water (400 *μ*L). The reaction was heated to 50 ^o^C. After 48h, the as-formed precipitate was concentrated under reduced pressure employing a rotary evaporator before being washed with heptane (*2x*, 20 mL) and acetone (*1x*, 10 mL). The solid formed was dissolved in chloroform and washed only once with water to remove inorganic impurities. The desired product was obtained as a brown solid (189 mg; 0.27 mmol; *90% yield*).

**TLC:** *R_f_* = 0.1 (100% MeCN, SiO_2_, *p-Anisaldehyde*).

**^1^H NMR (400 MHz, CDCl_3_) δ_H_:** 10.3 (s, 1H),7.33 (s, 2H), 4.35 (t, *J* = 7.0Hz, 4H), 1.97 – 1.90 (m, 4H), 1.34 – 1.18 (m, 60H), 0.87 (t, *J* = 7.0Hz, 6H)ppm.^[[11]](#footnote-11)^ *[See spectrum](#nmriodine1)*

**^13^C NMR (100 MHz, CDCl_3_) δ_C_:** 136.1, 122.2, 50.0, 31.8, 30.8, 30.1, 29.5, 29.49, 29.47, 29.45, 29.4, 29.3,29.2, 29.17, 28.8, 26.1, 22.5, 13.9, 28.9, 26.4, 22.6, 14.0ppm. [*See spectrum*](#nmriodine2)

# **IR (ATR, cm^-1^):** 3194, 3054, 2985, 2859, 1550, 1495, 1325, 1265, 847,823, 735, 728, 659. See sub-section *6. Infrared spectroscopy studies.*

**DSC:** *T*_mp_ = 64.82 ^o^C.

**TGA:** *T_d_*= 291.23 ^o^C.

**HRMS (ESI^+^):** Calcd. for [C_39_H_77_N_2_]^+^: 573.6081, found: 573.6071.

**HRMS (ESI^-^):** Calcd. for [I]^-^: 126.9050, found: 126.9051.

**1,3-dioctadecyl-1H-3|4-imidazol-1-ium bromide ([C_18_C_18_Im]^+^/Br^-^)**

To a flask equipped with a stir bar was added *1-octadecyl-1H-imidazole* (727 mg; 2.27 mmol; 1 equiv.) and MeCN (23 mL, 0.1 mol·L^-1^). Sequentially, 1-bromo-octadecane (835.5 mg; 2.5 mmol; 1.1 equiv.) was added. The reaction was allowed to warm to 110 ^o^C for 12 hours. Finally, the flask was cooled to room temperature, and MeCN was removed under reduced pressure using a rotary evaporator. The brown solid was washed with (*1x* 30 mL) hexane and (*3x* 30 mL) diethyl ether. The desired product was obtained as a brown solid (1.26 g; 1.93 mmol; *85% yield*).

**^1^H NMR (400 MHz, CDCl_3_) δ_H_:** 10.52 (s, 1H),7.40 (s, 2H), 4.33 (t, *J* = 7.0 Hz, 4H), 1.91 – 1.88 (m, 4H), 1.30 – 1.22 (m, 60H), 0.85 (t, *J* = 7.0 Hz, 6H)ppm. ^[[12]](#footnote-12)^ [*See spectrum*](#nmrbr1)

**^13^C NMR (100 MHz, CDCl_3_) δ_C_:** 137.3, 121.7, 50.1, 31.8, (2x) 29.6, 29.5, 29.4. (2x) 29.3, 28.9, 26.2, 22.6, 14.0 ppm. [*See spectrum*](#nmrbr2)

**IR (ATR, cm^-1^):** 3200, 2950, 2859, 1550, 1495, 1345, 1266, 847, 823, 715, 768, 649. See sub-section *6. Infrared spectroscopy studies.*

**DSC:** T_mp_ = 68.27 ^o^C.

**TGA:** *T_d_*= 282.37 ^o^C.

**HRMS (ESI^+^):** Calcd. for [C_39_H_77_N_2_]^+^: 573.6081, found: 573.6071.

**HRMS (ESI-):** Calcd. for [Br]^-^: 78.9189, found: 78.9189.

**General procedure A - Ion exchange reaction**

The ion exchange reaction is more efficiently performed when using an ion exchange resin.^[[13]](#footnote-13)^ In this work, an ion exchange resin was not employed; consequently, the purification process was challenging. It should be noted that the ionic liquid containing bromide as an anion was used as the starting material for the ion exchange reactions since the solubility of NaBr in water is greater than that of NaI,^[[14]](#footnote-14)^ a fact that promotes the removal of impurities inorganic via extraction using water and chloroform as the organic phase. For the ion exchange reactions, the temperature used was 50 ^o^C, and a reaction time of 48 hours to guarantee complete solubilisation of the reaction components. At the end of the reaction, the solvent is removed under reduced pressure, and chloroform is added to solubilize only the ionic liquid, not the inorganic salts. Subsequently, only an extraction with water is performed to avoid considerable losses of the ionic liquid in the water.

**1,3-dioctadecyl-1H-3|4-imidazol-1-ium bis((trifluoromethyl)sulfonyl)amide ([C_18_C_18_Im]^+^/[NTf₂]⁻)**

The ion exchange reaction was employed for the synthesis of this compound (General procedure A). A mixture of *1,3-dioctadecyl-1H-3|4-imidazol-1-ium iodine* (2.14 g; 3 mmol; 1 equiv.) in EtOH (100 mL, 0.03 mol·L^-1^) was added to *lithium bis(trifluoromethylsulfonyl)imide salt* (2.80 g; 9.75 mmol; 3.2 equiv.) and water (4.3 mL; 0.7 mol·L^-1^). The reaction was heated to 50 ^o^C. After 48h, the as-formed precipitate was concentrated employing a rotavap prior to being washed with heptane (*2x*, 20 mL) and acetone (*1x*, 10 mL). The solid formed was dissolved in chloroform and washed only once with water to remove inorganic impurities. The desired product was obtained as a white solid (2.54 g; 2.97 mmol; *65% yield*).

**^1^H NMR (400 MHz, CDCl_3_) δ_H_:** 8.88 (s, 1H),7.27 (s, 2H), 4.19 (t, *J* = 7.0 Hz, 4H), 1.88 – 1.85 (m, 4H), 1.30 – 1.25 (m, 60H), 0.88 (t, *J* = 7.0 Hz, 6H)ppm.^[[15]](#footnote-15)^ *[See spectrum](#nmrntf21)*

**^13^C NMR (100 MHz, CDCl_3_) δ_C_:** 135.9, 124.6, 122.0, 121.4, 118.2, 118.2, 115.03, 50.3, 31.9, 30.1, 29.7, (2x) 29.6, 29.5, (2x) 29.3, 28.9, 26.1, 22.7, 14.1 ppm. *[See spectrum](#nmrntf22)*

**^19^F NMR (376 MHz, CDCl_3_) δ_F_:** -78.95 ppm. [*See spectrum*](#nmrntf23)

**IR (ATR, cm^-1^):** 3222, 3100, 2955, 2845, 3194, 3054, 2985, 2859, 1550, 1495, 1401, 1361, 1355, 1331, 1265, 1002, 47,823, 735, 728, 659. See sub-section *6. Infrared spectroscopy studies*

**DSC:** T_mp_ = 71.52 ^o^C.

**TGA:** *T_d_*= 420.97 ^o^C.

**HRMS (ESI^+^):** Calcd. for [C_39_H_77_N_2_]^+^: 573.6071, found: 573.6081.

**HRMS (ESI^-^):** Calcd. for [NTf_2_]^-^: 279.9178, found: 279.9176.

**1,3-dioctadecyl-1H-3l4-imidazol-1-ium dicyanamide ([C_18_C_18_Im]^+^/[N(CN)_2_]⁻)**

The ion exchange reaction was employed for the synthesis of this compound. To a stirred solution of *1,3-dioctadecyl-1H-3l4-imidazol-1-ium iodine* (3.22 g; 4.59 mmol; 1 equiv.) in DCM (100 mL, 0.04 mol·L^-1^) was added sodium dicyanamide salt (612.97 mg; 6.88 mmol; 1.5 equiv.) in 50 mL of water. The reaction was heated to 50 ^o^C. After 48 h, the as-formed white precipitate was concentrated under reduced pressure using a rotary evaporator and then washed with DCM (*3x*, 20 mL) and diethyl ether (*1x*, 20 mL). The solid formed was dissolved in chloroform and washed only once with water to remove inorganic impurities. The desired product was obtained as a white solid (1.61 g; 2.51 mmol; *55% yield*).

**^1^H NMR (400 MHz, CDCl_3_) δ_H_:** 9.83 (s, 1H),7.33 (d, *J* = 1.61 Hz, 2H), 4.31 (t, *J* = 7.0 Hz, 4H), 1.94 – 1.90 (m, 4H), 1.33 – 1.24 (m, 60H), 0.87 (t, *J* = 7.0 Hz, 6H)ppm.^[[16]](#footnote-16)^ *[See spectrum](#nmrncn1)*

**^13^C NMR (100 MHz, CDCl_3_) δ_C_:** 136.4, 121.9, 119.9, 50.4, 31.9, 30.2, 29.7, (2x) 29.6, 29.5, 29.4, 29.3, 28.9, 26.2, 22.6, 14.1 ppm. [*See spectrum*](#nmrncn2)

**IR (ATR, cm^-1^):** 3124, 3064, 2975, 2839, 2260, 2235, 2210, 1550, 1495, 1355, 1245, 847,823, 735, 788, 639.

**DSC:** T_mp_ = 64.72 ^o^C.

**TGA:** *T_d_*= 295.57 ^o^C.

**HRMS (ESI^+^):** Calcd. for [C_39_H_77_N_2_]^+^: 573.6081, found: 573.6071.

**HRMS (ESI^-^):** Calcd. for [NCN_2_]^-^: 66.0098, found: 66.0096.

**4. NMR characterisation**

**^1^H NMR (400 MHz, CDCl_3_)**

**H_4_**

**H_3_**

**H_5_**

**H_6_**

**H_7_**

**H_2_**

**H_1_**

**^1^H NMR (400 MHz, CDCl_3_) δ_H_:** 8.27 (s, 1H),7.1 (s, 1H), 7.0 (s, 1H), 4.1 (t, *J* = 7.0Hz, 2H), 1.82 – 1.79 (m, 2H), 1.29 – 1.23 (m, 30H), 0.86 (t, *J* = 7.0Hz, 3H)ppm.

**^13^C NMR (100 MHz, CDCl_3_)**

**C_17_**

**C_16_**

**C_5-_C_15_**

**C_4_**

**C_3_**

**C_2_**

**C_1_**

**^13^C NMR (100 MHz, CDCl_3_) δ_C_:** 136.5, 125.6, 119.5, 48.0, 31.8, 30.8, 29.6, 29.58, 29.57, 29.5, 29.4, 29.3, 29.2, 28.9, 26.4, 22.6, 14.0ppm.

**^1^H NMR (400 MHz, CDCl_3_)**

**H_5_**

**H_6_**

**H_4_**

**H_3_**

**H_2_**

**H_1_**

**^1^H NMR (400 MHz, CDCl_3_:** 7.26 ppm**) δ_H_:** 10.3 (s, 1H, H_1_),7.33 (s, 2H), 4.35 (t, *J* = 7.0Hz, 4H, H_3_), 1.97 – 1.90 (m, 4H, H_4_), 1.34 – 1.18 (m, 60H, H_5_), 0.87 (t, *J* = 7.0Hz, 6H, H_6_)ppm.

**^13^C NMR (100 MHz, CDCl_3_)**

**^13^C NMR (100 MHz, CDCl_3_: 77 ppm) δ_C_:** 136.1(C_1_), 122.2(C_2_), 50.0(C_3_), 31.8(C_4_), 30.8, 30.1, 29.5, 29.49, 29.47, 29.45, 29.4, 29.3,29.2, 29.17, 28.8(C_17_-C_5_), 26.1(C_18_), 22.5(C_19_), 13.9, 28.9, 26.4, 22.6, 14.0(C_20_)ppm.

**C_18_**

**C_19_**

**C_20_**

**C_5_ -C_17_**

**C_4_**

**C_3_**

**C_2_**

**C_1_**

**^1^H NMR (400 MHz, CDCl_3_)**

**H_5_**

**H_6_**

**H_4_**

**H_3_**

**H_2_**

**H_1_**

**^1^H NMR (400 MHz, CDCl_3_: 7.26 ppm) δ_H_:** 10.52 (s, 1H, H_1_),7.40 (s, 2H, H_2_), 4.33 (t, *J* = 7.0 Hz, 4H, H_3_), 1.91 – 1.88 (m, 4H, H_4_), 1.30 – 1.22 (m, 60H, H_5_), 0.85 (t, *J* = 7.0 Hz, 6H, H_6_)ppm.

**^13^C NMR (100 MHz, CDCl_3_)**

**C_5_ -C_17_**

**C_20_**

**C_19_**

**C_18_**

**C_4_**

**C_2_**

**C_1_**

**C_3_**

**^13^C NMR (100 MHz, CDCl_3_: 77 ppm) δ_C_:** 137.3 (C_1_), 121.7 ppm (C_2_), 50.1 (C_3_), 31.8(C_4_), (2x) 29.6, 29.5, 29.4. (2x) 29.3, 28.9(C_17_-C_5_), 26.2(C_18_), 22.6(C_19_), 14.0(C_20_) ppm.

**^1^H NMR (400 MHz, CDCl_3_)**

**^^**

**H_6_**

**H_5_**

**H_4_**

**H_2_**

**H_1_**

**H_3_**

**^1^H NMR (400 MHz, CDCl_3_: 7.26 ppm) δ_H_:** 8.88 (s, 1H, H_1_),7.27 (s, 2H, H_2_), 4.19 (t, *J* = 7.0 Hz, 4H, H_3_), 1.88 – 1.85 (m, 4H, H_4_), 1.30 – 1.25 (m, 60H, H_5_), 0.88 (t, *J* = 7.0 Hz, 6H, H_6_)ppm.

**^13^C NMR (100 MHz, CDCl_3_****)**

**C_18_**

**C_2(F)_**

**C_2_**

**C_3_**

**C_4_**

**C_20_**

**C_19_**

**C_5_ -C_17_**

**C_1_**

**^13^C NMR (100 MHz, CDCl_3_: 77 ppm) δ_C_:** 135.9(C_1_), 122.0(C_2_), 121.4 (q, *J_C-F_ =* 320 Hz, C_2(F)_), 50.3(C_3_), 31.9(C_4_), 30.1, 29.7, (2x) 29.6, 29.5, (2x) 29.3, 28.9(C_17_-C_5_), 26.1, 22.7(C_19_), 14.1 (C_20_) ppm.

**^19^F NMR (376 MHz, CDCl_3_)**

**^^****^^**

**^1^H NMR (400 MHz, CDCl_3_)**

**^^****^^**

**H_1_**

**H_2_**

**H_3_**

**H_4_**

**H_5_**

**H_6_**

**^1^H NMR (400 MHz, CDCl_3_: 7.26 ppm) δ_H_:** 9.83 (s, 1H, H_1_),7.33 (d, *J* = 1.61 Hz, 2H, H_2_), 4.31 (t, *J* = 7.0 Hz, 4H, H_3_), 1.94 – 1.90 (m, 4H, H_4_), 1.33 – 1.24 (m, 60H, H_5_), 0.87 (t, *J* = 7.0 Hz, 6H, H_6_)ppm

**^13^C NMR (100 MHz, CDCl_3_)**

**^^****^^**

**C_2(anion)_**

**C_19_**

**C_20_**

**C_18_**

**^13^C NMR (100 MHz, CDCl_3_) δ_C_:** 136.4 (C_1_)., 121.9 (C_2_), 119.9 (_C2anion_), 50.4 (C_3_), 31.9 (C_4_), 30.2, 29.7, (2x) 29.6, 29.5, 29.4, 29.3, 28.9 (C_17_-C_5_), 26.2 (C_18_), 22.6 (C_19_), 14.1 (C_20_) ppm.

**4.1 NMR spectra comparison**

**Figure S9.** Change in chemical shift (^1^H) as a function of the anion structure.

By focusing on a range of high ^1^H-NMR chemical shift values for the four A-TILCs, the interaction between the H_2_ proton located on the polar head of the imidazolium and the anion is probed, as the values range from 10.65 ppm for the bromide TILC, 10.40 ppm for the iodide TILC, 9.84 ppm for the dicyanamide TILC and 8.91 ppm for the largest anion [NTf₂]⁻. The smaller the size of the anion, the higher the resulting chemical shift. These results indicate that the proton H_2_-anion interactions are stronger for TILCs composed of smaller anions. These observations show that the anions are highly localized around this proton as the H_2_-anion chemical shift undergoes very significant changes by varying the nature of the anion.

**Figure S10.** Change in chemical shift (^13^C) as a function of the anion structure.

# **4.2 Bidimensional NMR**

***g-COSY***

**^^**

**Table S4**. COSY bidimensional NMR for C_18_-C_18_-Im^+^/Br^-^

| Signal ^1^H (ppm) | | COSY | |
| --- | --- | --- | --- |
| H_1_ (10.30, s, 1H) | - | |  |
| H_2_ (7.33, s, 2H) | - | |  |
| H_3_ (4.35, t, *J* = 7.0Hz, 4H) | H_3_-H_4_ | |  |
| H_4_ (1.97 – 1.90, m, 4H) | H_4_-H_3_ and H_4_-H_5_ | |  |
| H_5_ (1.34-1.18, m, 60H) | H_5_-H_4_, H_5_-H_6_ | |  |
| H_6_ (0.87, t, *J =* 7.0Hz, 6H) | H_6_-H_5_ | |  |

***g-HSQC***

**^^**

**Table S5**. HSQC bidimensional NMR for C_18_-C_18_-Im^+^/Br^-^

| Signal ^1^H (ppm) | | Signal ^13^C (ppm) | HSQC |
| --- | --- | --- | --- |
| H_1_ (10.30, s, 1H) | C_1_ (136.1) | - |  |
| H_2_ (7.33, s, 2H) | C_2_ (122.2) | C_2_-H_2_ |  |
| H_3_ (4.35, t, *J* = 7.0Hz, 4H) | C_3_ (50) | C_3_-H_3_ |  |
| H_4_ (1.97 – 1.90, m, 4H) | C_4_ (31.8) | C_4_-H_4_ |  |
| H_5_ (1.34-1.18, m, 60H) | C_5_, C_6_, C_7_, C_8_, C_9_, C_10_, C_11_, C_12_, C_13_, C_14_, C_15_, C_16_, C_17_ (aliphatic carbons, 30.8-28.8 ppm) | C_5_, C_6_, C_7_, C_8_, C_9_, C_10_, C_11_, C_12_, C_13_, C_14_, C_15_, C_16_, C_17_–H5 |  |
| H_6_ (0.87, t, *J =* 7.0Hz, 6H,) | C_18_ (26.1) | C_18_-H_5_ |  |
| - | C_19_ (22.5) | C_19_-H_5_ |  |
| - | C_20_ (13.9 – 14 ppm) | C_20_-H_6_ |  |

***g-HMBC***

**^^**

**Table S6**. HMBC bidimensional NMR for C_18_-C_18_-Im^+^/Br^-^

| Signal ^1^H (ppm) | | Signal ^13^C (ppm) | HMBC |
| --- | --- | --- | --- |
| H_1_ (10.30, s, 1H) | C_1_ (136.1) | C_1_-H_2_ and H_3_ |  |
| H_2_ (7.33, s, 2H) | C_2_ (122.2) | C_2_-H_1_ and H_3_ |  |
| H_3_ (4.35, t, *J* = 7.0Hz, 4H) | C_3_ (50) | C_3_-H_2_ |  |
| H_4_ (1.97 – 1.90, m, 4H) | C_4_ (31.8) | C_4_-H_5_ |  |
| H_5_ (1.34-1.18, m, 60H) | C_5_, C_6_, C_7_, C_8_, C_9_, C_10_, C_11_, C_12_, C_13_, C_14_, C_15_, C_16_, C_17_ (aliphatic carbons, 30.8-28.8 ppm) | C_5_, C_6_, C_7_, C_8_, C_9_, C_10_, C_11_, C_12_, C_13_, C_14_, C_15_, C_16_, C_17_–H_5_, H_3_, H_4_ |  |
| H_6_ (0.87, t, *J =* 7.0Hz, 6H,) | C18 (26.1) | C_18_-H_6_ |  |
| - | C_19_ (22.5) | C_19_-H_5_ |  |
| - | C_20_ (13.9 – 14 ppm) | C_20_-H_5_ |  |

# **5. Electrospray ionization Mass Spectrometry (MS-ESI)**

**
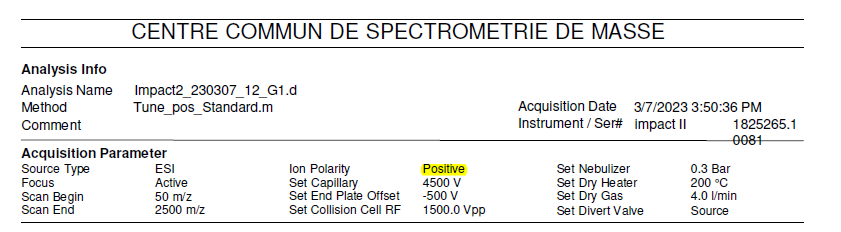
**


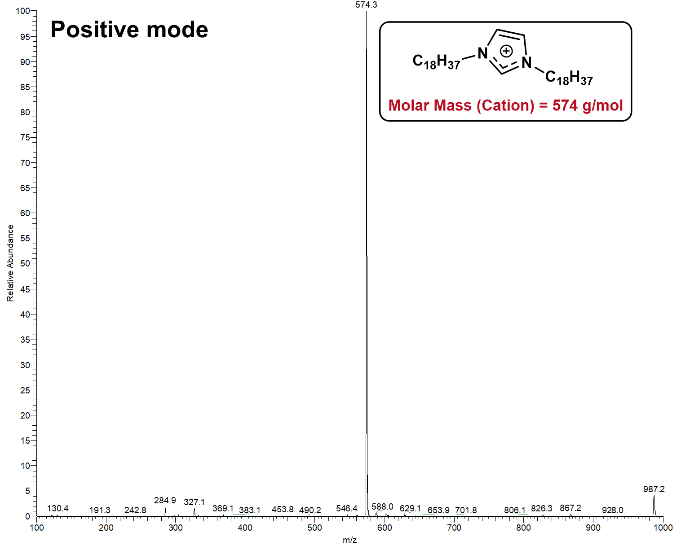


**Figure S11.** Mass spectrometry-EIS spectrum (+ mode).

In the negative mode (-mode), only the signal associated with the [NTf₂]⁻ anion could be detected onto the MS-ESI spectrum because the other anions were not large enough to be detected. Mass spectroscopy also shows the presence of a single intensity peak at m/z = 279.9 with a relative abundance of 100%. The other peaks are background noise (< 10% relative abundance). This peak corresponds well to the theoretical molar mass of the [NTf₂]⁻ anion (280.15 g·mol^-1^). Mass spectrometry indicates that the compound (in the - mode measurement) is pure.


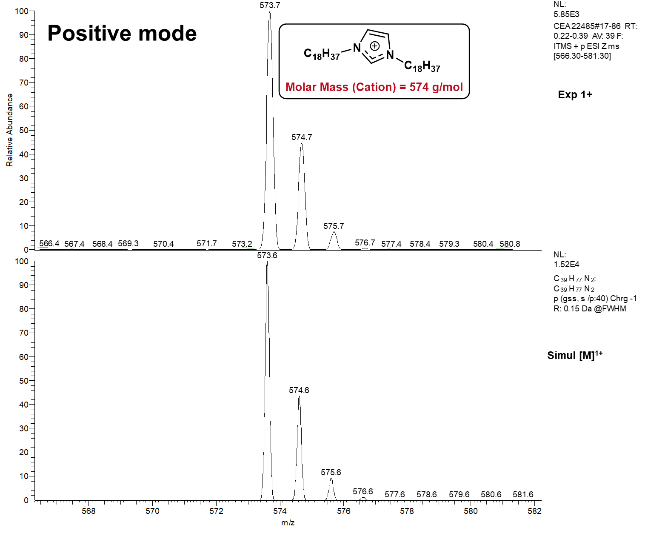


**Figure S12.** Mass spectrometry-EIS spectrum (+ mode): Magnification of the region between 568-582 m/z.


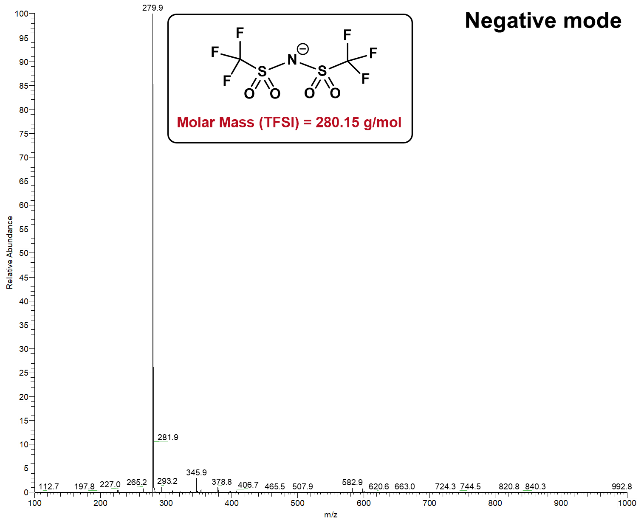


**Figure S13.** Mass spectrometry-EIS spectrum (- mode).

In the negative mode (-mode), only the signal associated with the NTf_2_^-^ anion could be detected onto the MS-ESI spectrum because the other anions were not large enough to be detected. Mass spectroscopy also shows the presence of a single intensity peak at m/z = 279.9 with a relative abundance of 100%. The other peaks are background noise (< 10% relative abundance). This peak corresponds well to the theoretical molar mass of the NTf_2_^-^ anion (280.15 g·mol^-1^). Mass spectrometry indicates that the compound (in the - mode measurement) is pure.


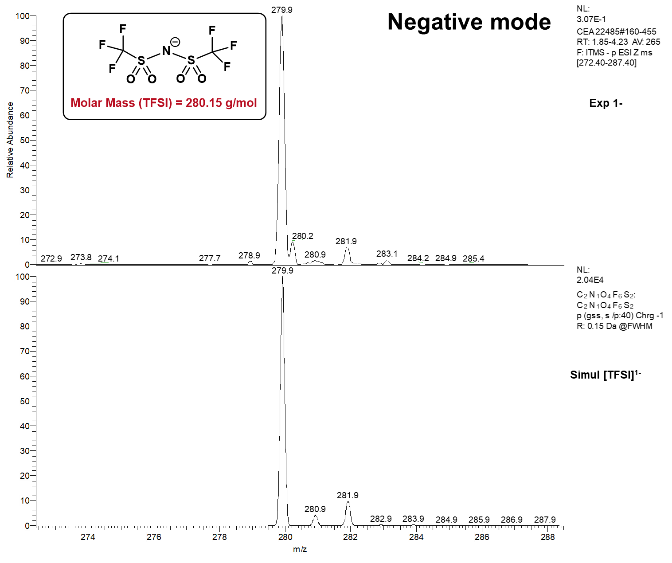


**Figure S14.** Mass spectrometry-EIS spectrum (- mode): Magnification of the 274-286 m/z region.


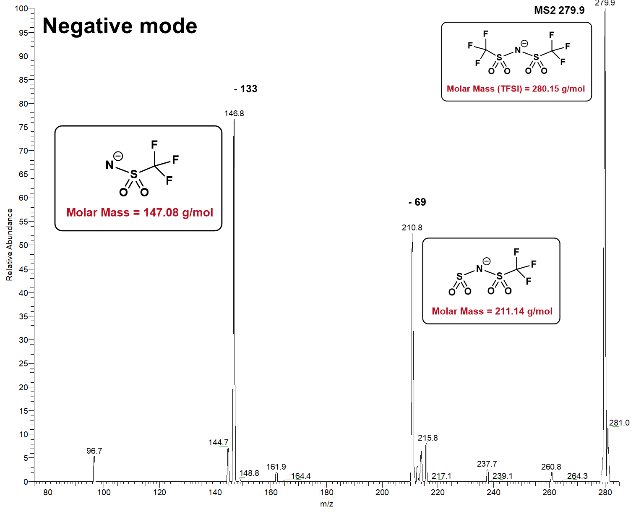


**Figure S15.** Mass spectrometry-EIS spectrum (- mode): Possible Fragments.


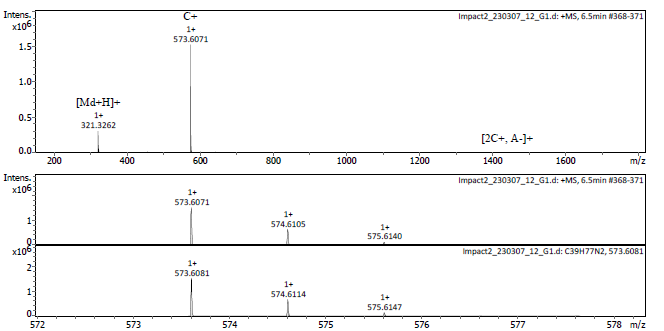

**Figure S16a.** Mass spectrometry-EIS spectra (+ mode) obtained for TILCs containing [N(CN)_2_]⁻, Br^-^, and I^-^.

**Figure S16b.** Mass spectrometry-EIS spectra (- mode) obtained for TILCs containing [N(CN)_2_]⁻, Br^-^, and I^-^.

# **6. Infrared spectroscopy studies**


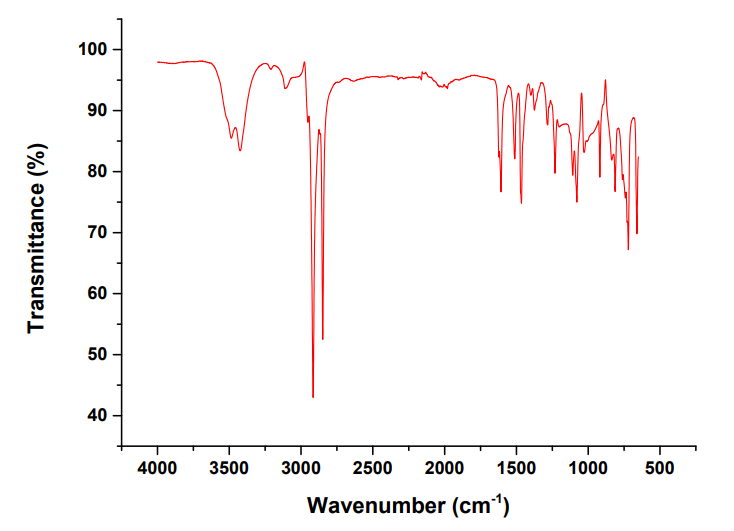


***Angular deformation 1455 cm^-1^***

***Typical primary and secondary carbons 2945 cm^-1^***

***Aliphatic C-H 3026 cm^-1^***

**Figure S17.** Infrared spectrum @ 25°C (4000-600 cm^-1^) for an intermediate compound which chemical structure is depicted as an inset within the FT-IR spectrum.


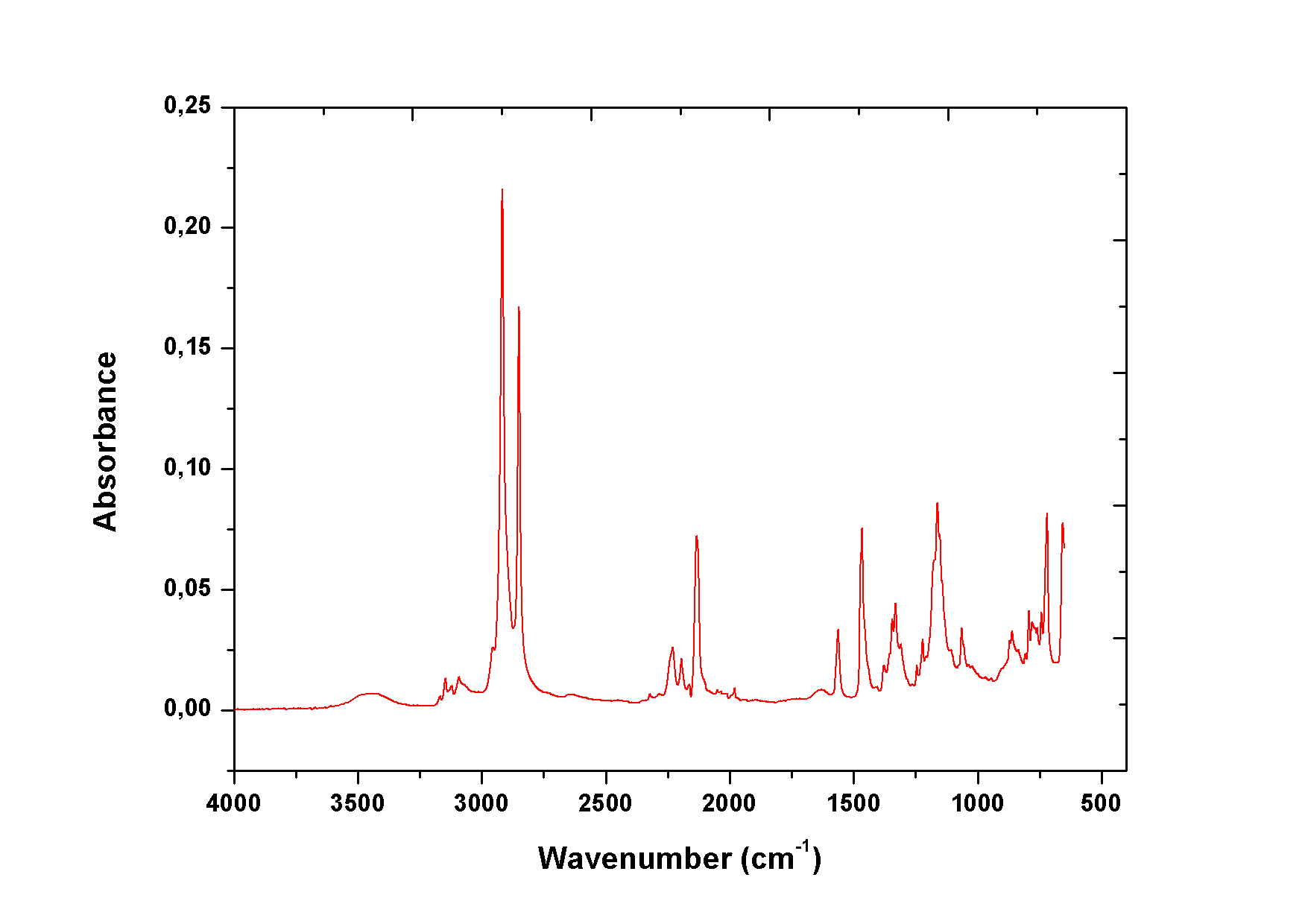


**C-N 1195 cm^-1^**

**N=C 2275-2250 cm^-1^**

**C-N nitrile 2245 cm^-1^**

**Figure S18.** Infrared spectra for[C_18_C_18_Im]^+^/[N(CN)_2_]⁻ : **(i)** at 25 °C (4000-600 cm^-1^) and **(ii)** from 30 °C to 140 °C (700-900 cm^-1^).


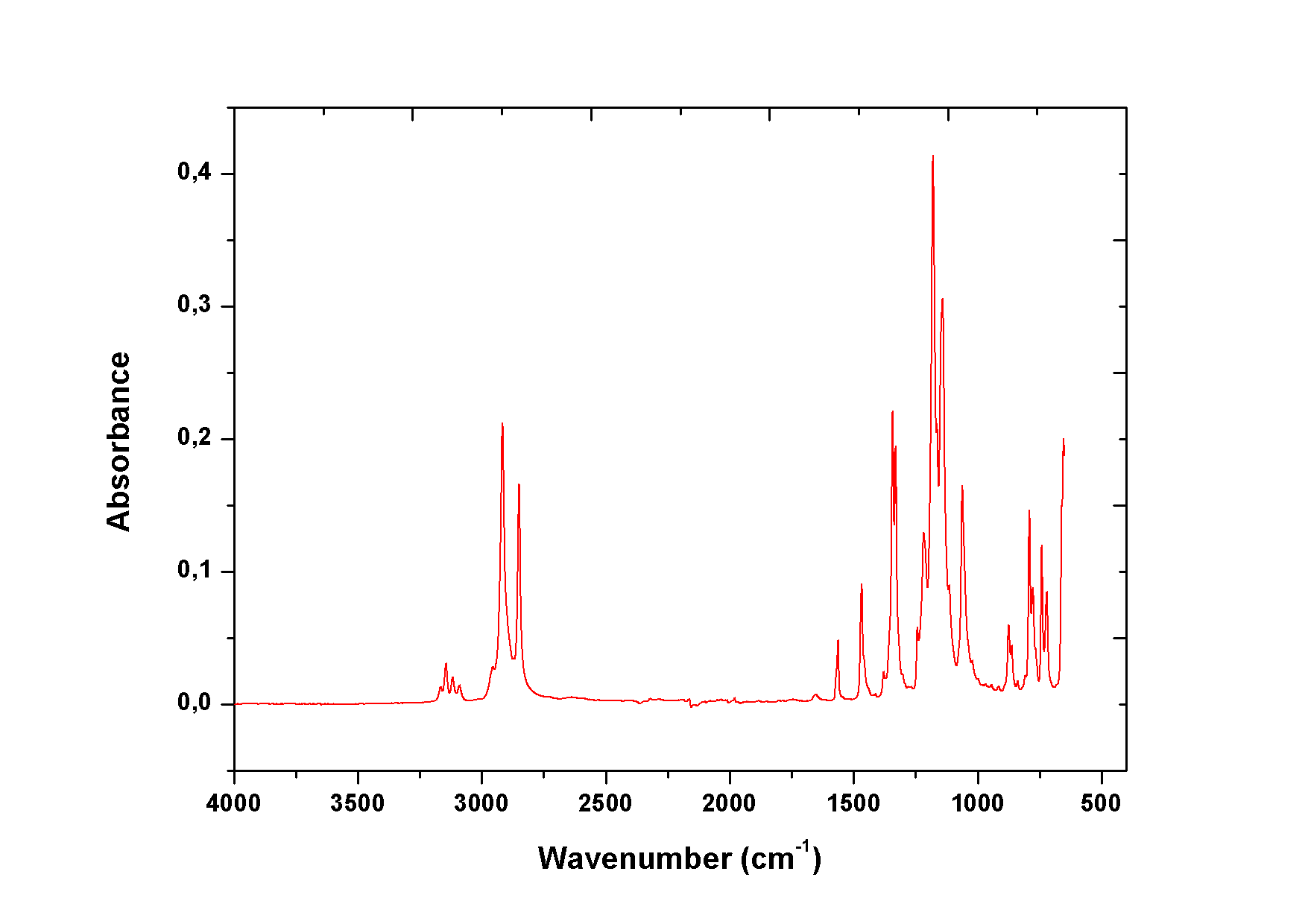


**C-F 1400-100 cm^-1^**

**S=O 1326 cm^-1^**

**S=O 1059 cm^-1^**

**S=O 1159 cm^-1^**

**Figure S19.** Infrared spectra for[C_18_C_18_Im]^+^/[NTf₂]⁻: **(i)** at 25 °C (4000-600 cm^-1^) and **(ii)** from 30 °C to 140 °C (700-900 cm^-1^).


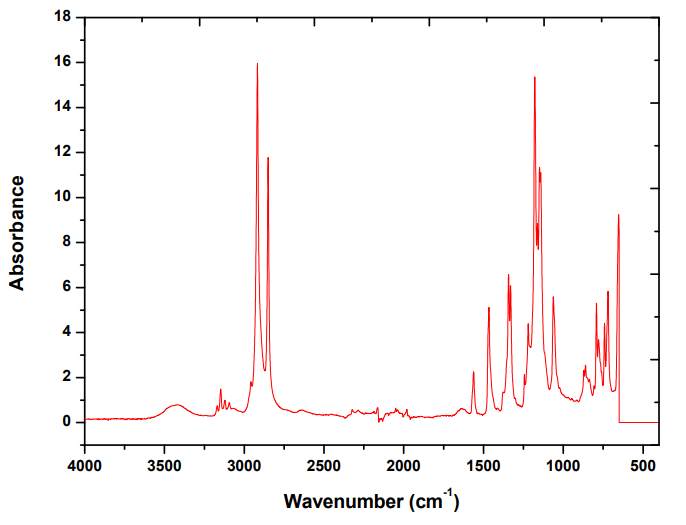

***Typical primary and secondary carbons 2945 cm^-1^***

***Angular deformation 1455 cm^-1^***

**“Rocking” 726 cm^-1^**

***Aliphatic C-H 3026 cm^-1^***

**Figure S20.** Infrared spectra for [C_18_C_18_Im]^+^/Br^-^: **(i)** at RT=25 °C (4000-600 cm^-1^) and **(ii)** from 30 °C to 140 °C (700-900 cm^-1^).


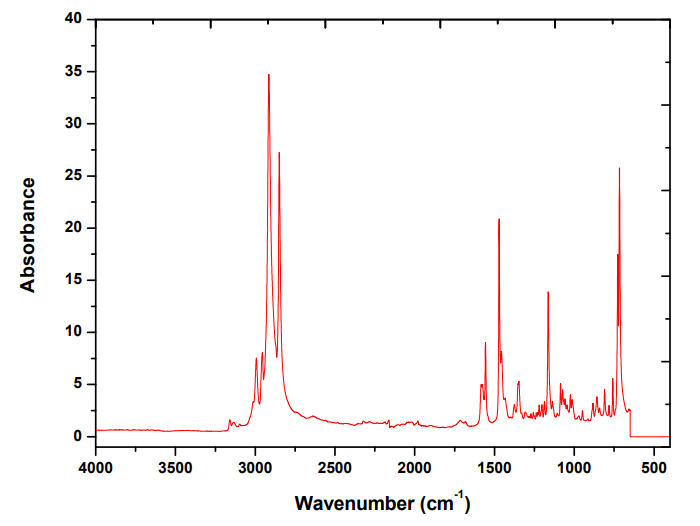

**Figure S21.** Infrared spectra for [C_18_C_18_Im]^+^/I^-^: **(i)** at RT=25 °C (4000-600 cm^-1^) and **(ii)** from 30 °C to 140 °C (700-900 cm^-1^).

# **7. Thermogravimetric Analysis (TGA) summary**

We performed a TGA study to investigate the thermal properties of the synthesized TILCs. Since the cation was identical across all the synthesized ionic liquids, the anion was found to play a crucial role in determining their thermal stability, as expected. On the one hand, salts containing halide counterions exhibited reduced thermal stability, likely due to the nucleophilic and basic nature of the halides (see **Table S6,** line 1 and 2); on the other hand, [N(CN)_2_]⁻ and [NTf₂]⁻ as counter-anions showed higher thermal stability than halides (see **Table S6**, line 3 and 4). It should be mentioned that the TILCs with the highest degradation temperature was [NTf₂]⁻, perhaps due to the mesomeric phenomenon that reduces the basicity and nucleophilicity of the anion (See **Figure S21**).

**Figure S22.** TGA thermograms of the synthesized TILCs. Insets: Chemical structures and First derivative signal as a function of temperature.

**Table S7**. Summary table of degradation temperatures at 5% (T_5%_) and degradation temperatures (*T_d_*) extracted at the minimum of the 1^st^ derivative signal with respect to temperature. Errors are due to the accuracy of the balance and the heating rate.

| *A-TILC* | *T_5%_ (°C)* | *T_d_ (°C)* | *Error of T_d_ (%)* |
| --- | --- | --- | --- |
| [C_18_C_18_Im]^+^C_18_C_18_Im^+^/Br^-^ | 233 | 283 | ±8.5 |
| [C_18_C_18_Im]^+^C_18_C_18_Im^+^/I^-^ | 252 | 292 | ±8.8 |
| [C_18_C_18_Im]^+^/[N(CN)_2_]⁻ C_18_C_18_Im^+^/N(CN)_2_^-^ | 256 | 296 | ±8.9 |
| [C_18_C_18_Im]^+^/[NTf₂]⁻C_18_C_18_Im^+^/NTf_2_^-^ | 381 | 422 | ±12.7 |

The degradation temperature at 5% mass loss increases with the increase in the size of the anion (Br^-^ (233 °C) < I^-^ (252 °C) < [N(CN)_2_]⁻ (256 °C) < [NTf₂]⁻(381 °C). These degradation temperatures are well-above 150 °C (which is the maximum temperature reached during electrochemical impedance spectroscopy (EIS) measurements), which ensures that the chemical integrity TILCs during EIS characterizations. Static thermal stability was also studied.

[NTf₂]⁻ is known for its excellent thermal stability due to resonance delocalization and poor nucleophilicity, which contributes to the enhanced thermal stability of the TILC.^[[17]](#footnote-17)^ Larger anions like[NTf₂]⁻, [PF_6_]⁻, or [BF_4_]⁻ tend to have more delocalized charges, leading to weaker ion pairing. Similarly, the [N(CN)_2_]⁻ while larger than I^-^ and Br^-^, has a unique chemical structure that could confer it with a higher thermal stability than expected for halides.^[[18]](#footnote-18)^ Typically, thermal degradation of TILCs involves multiple steps, including decomposition of the anion and/or cation, and possible formation of by-products. While weak ion pairing can reduce melting point and increase fluidity, thermal stability depends first on the intrinsic stability of the anion. Larger anions typically have a lower charge density than smaller anions. As a result, larger anions are less able to interact strongly with the imidazolium cation, which can decrease the thermal stability of the TILCs. However, in this case, the observed trend is opposite to what is expected, with larger anions having higher thermal stability. This is often observed in imidazolium-based ILs where the anion starts to decompose first—especially if it is less thermally stable.^[[19]](#footnote-19)^

# **8. Differential Scanning Calorimetry (DSC)**

DSC gives information on **(i)** phase transitions and on the number of mesophases in a TILC, **(ii)** the typology of the observed mesophases, as well as **(iii)** on the temperatures at which these transitions occur. The transitions in this type of materials are either of first order (melting or crystallisation or mesophase) or of second order (as for certain *mesophase-to-mesophase* transitions).

**Figure S23.** DSC Thermograms of [C_18_C_18_Im]^+^/X^-^ TILCs.

# **9. Polarised Optical Microscopy (POM)**

Melting and clearing points, as well as liquid crystalline (LC) phases (i.e.,mesophases), were characterized with a customized POM platform consisting of a DM/LM (Leica) microscope working both in transmission and reflection modes and equipped with a series of x2.5, x5, x10, x20, and x50 objectives, a KY-F75 (JVC) 3 CCD camera, a LTS350 (Linkam) hot-plate, a CI94 (Linkam) temperature controller, and a LNP (Linkam) cooling system. POM highlights the anisotropies present in a sample and shows the presence or absence of birefringence (arrangement of domains in different directions in space) using cross polarisers. The observed textures give information and indications on the nature of the formed mesophases. Each TILC was infiltrated (by capillary action) into commercially available (Instec Inc., Boulder, COP (USA) liquid crystal cells with ITO electrodes to allow In-Plane (IP) and Through-Plane (TP) electrochemical impedance spectroscopy (EIS). TILCs were first heated (scan rate 10 °C·min^-1^) under N_2_ till reaching their isotropic states and immediately cooled down 5 °C below their clearing temperatures (as determined by DSC) to avoid their chemical degradation. The POM imaging was then conducted during cooling scans, using customized (adjusted to each TILC) cooling scan rate (of *ca.* 2.5 to 0.5 °C·min^-1^) and appropriate annealing procedures to allow for the development of birefringent textures related to their mesomorphic behaviours and related properties (See **Figure S23**).

| 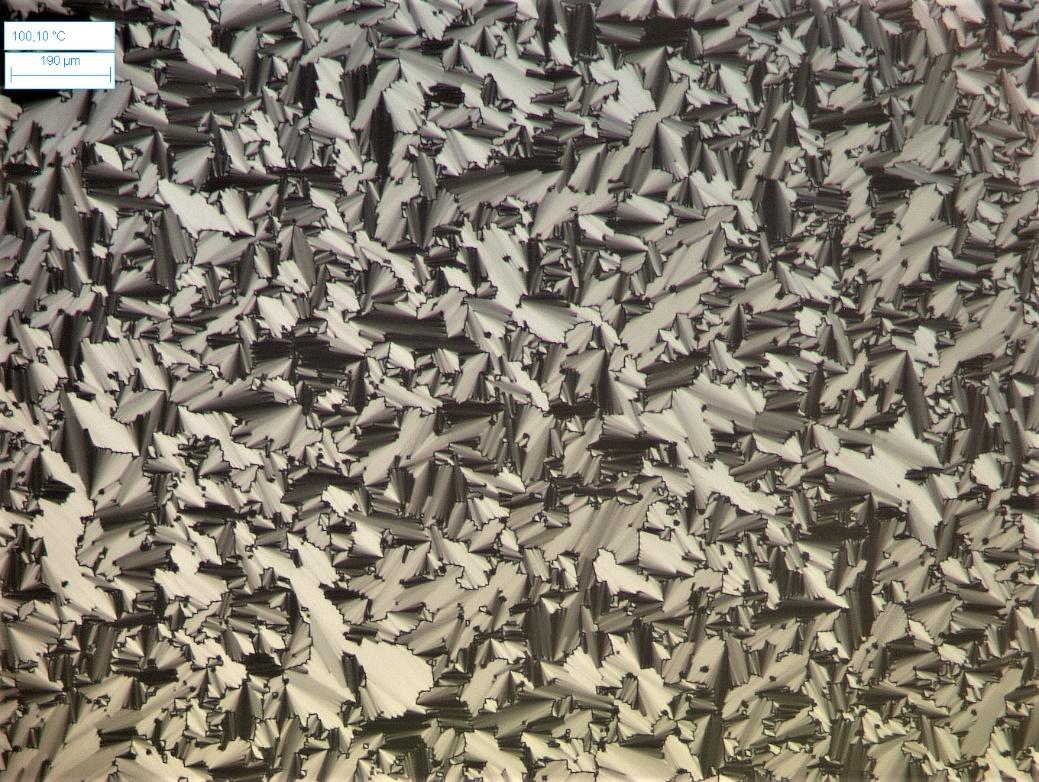 | 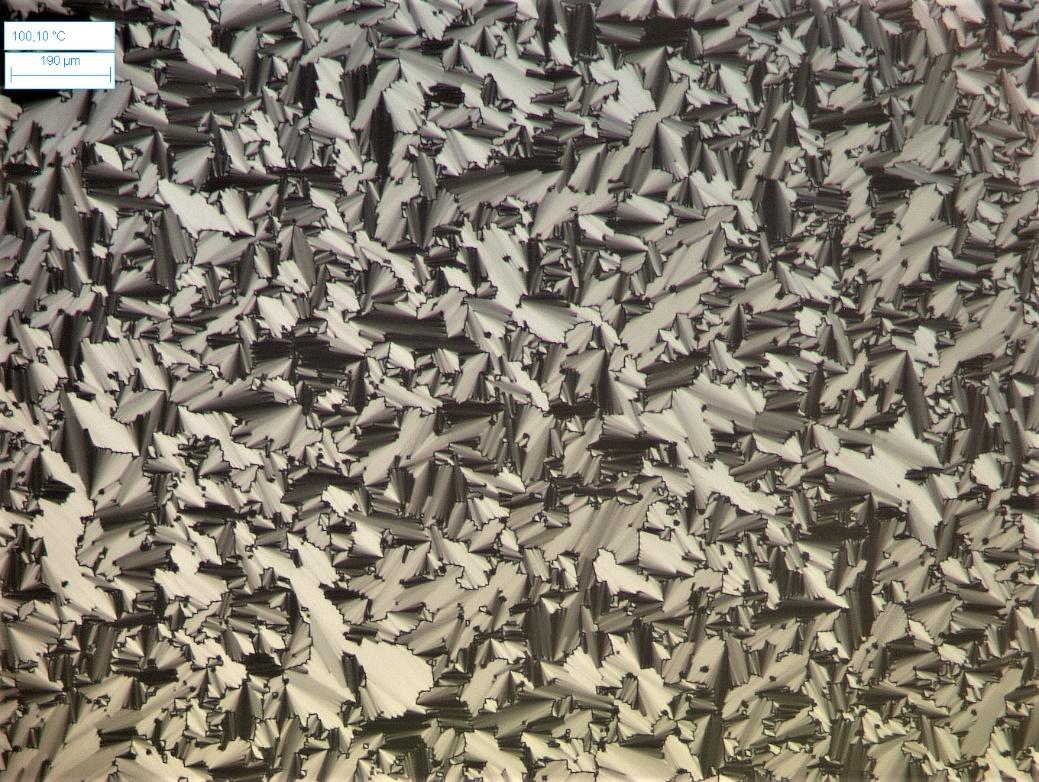 |
| --- | --- |
| 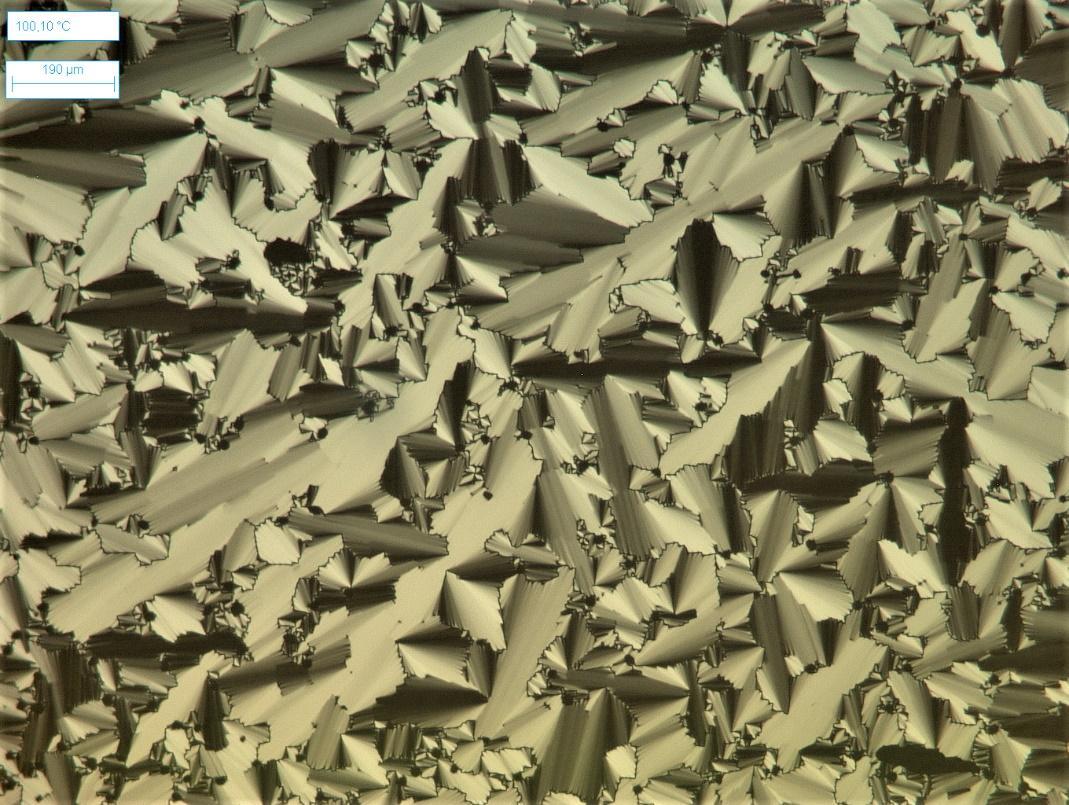 | 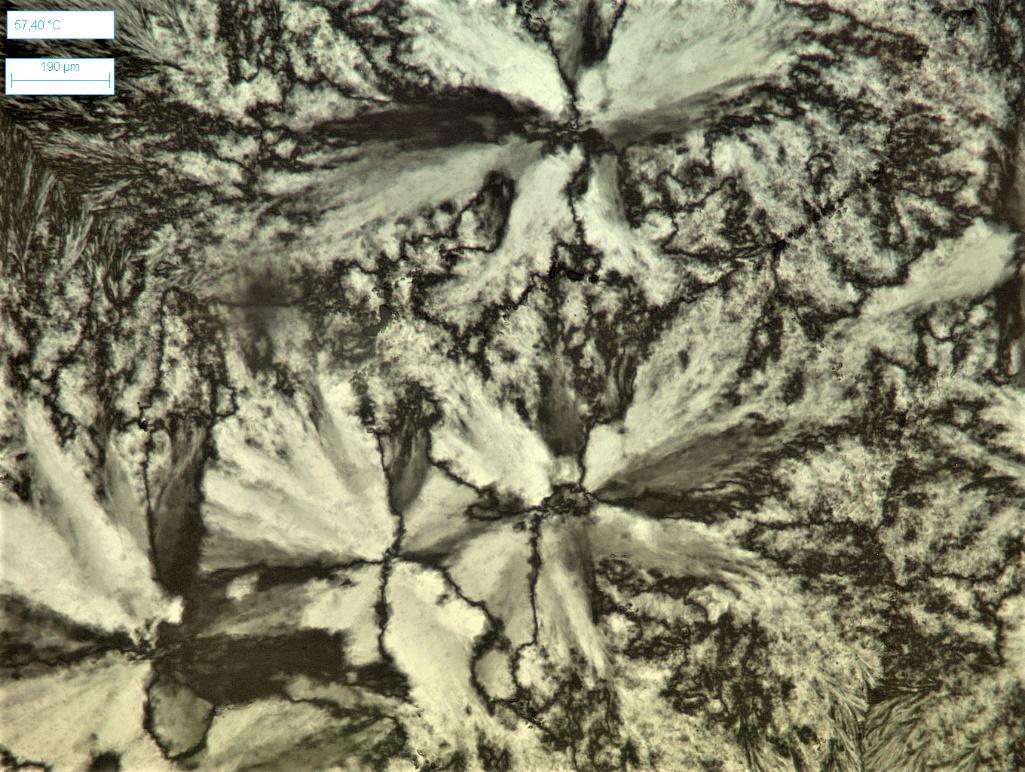 |

**Figure S24**: Polarised optical microphotographs imaged during the first cooling cycle at 100 °C from the isotropic state of [C_18_C_18_Im]^+^/Br^-^ (top left), [C_18_C_18_Im]^+^/I^-^ (top right), [C_18_C_18_Im]^+^/[N(CN)_2_]⁻ (bottom left) displayed focal conic fan textures (homogeneous alignment) while [C_18_C_18_Im]^+^/[NTf₂]⁻showed a mosaic texture at 57.4 °C. The scale bars are worth 190 microns.

The optical properties of the A-CLIT with the [NTf₂]⁻ anion were evaluated. During the first temperature cooling cycle, at 57.4 °C, it exhibited a birefringent mosaic texture (See **Figure S24**), characterised by a granular appearance under POM, with a multitude of birefringent domains of different size and shape, separated by sharp boundaries. Although a mosaic texture is more likely to be a lamellar mesophase with long-range intra-lamellar correlation (e.g., SmB_Cryst_ or SmE), it is difficult to draw a definitive conclusion about the nature of this mesophase from this texture alone.

# **10. Small- and Wide-Angle X-ray Scatterings (SWAXS)**

Variable temperature SAXS/WAXS (SWAXS(T)) measurements were performed at ESRF (European Synchrotron Radiation Facility, Grenoble, France) on the CRG BM02-D2AM and ID31 beamlines. Preliminary SAXS/WAXS characterisations were performed using an in-house X-ray diffusion/diffraction setup (IRIG/DePhy/MEM/SGX, Grenoble, France). The experimental conditions applied to obtain the data shown in the main text and in this Supplementary Information are described hereinafter.

*SWAXS(T) experiments performed using a home-made SAXS/WAXS setup at CEA-Grenoble/IRIG*/*DePhy/MEM/SGX*

Variable temperature SWAXS measurements in the transmission configuration were performed using a home-made (CEA-Grenoble/IRIG/DePhy/MEM/SGX) SAXS line consisting in a FR591-3kW rotating anode (Nonius) with Cu K_α_ radiation, a set of two Ni-filtered focusing mirrors (Xenocs), and a VÅNTEC-2000 2D detector (Brucker). The incident photon energy was adjusted to 8.04 keV, *i.e.,* a wavelength of *λ* = 0.15418 nm. The *sample-to-detector* distance (SDD) was set to 37 cm for covering a *ca.* 0.755 to 14.0 nm^-1^ scattering vector q-range. The 2D detector was off-centred to increase the *q*-range for anisotropic samples. Silver behenate (AgBe, CAS number: [2489-05-6]) was used for the *q*-range calibration of the 2D detector. The TILCs were sealed in home-made (copper brass-based) circular holders equipped with 20 microns-thick Kapton^®^ windows. Standard correction procedures were applied to the data for the background subtraction and normalisation. The contribution of the empty cell was subtracted from the scattering intensity of the studied samples. 2D images were converted into radial averages over the image center to yield the scattered intensity *I*(*q*) *vs.* scattering-vector modulus *q* using the [Datasqueeze](http://www.physics.upenn.edu/~heiney/datasqueeze/index.html) software. The intensity curves were scaled to the absolute intensities with the aid of a laboratory-calibrated Lupolen^®^ sample. For temperature-resolved measurements, samples were subjected to a heating/cooling rate of 1 °C·min^-1^ and equilibrated for 5 minutes at each temperature before data acquisition.

*SWAXS(T) experiments at the* [*CRG BM02-D2AM*](https://www.esrf.fr/UsersAndScience/Experiments/CRG/BM02) *SAXS/WAXS beamline at* [*ESRF*](https://www.esrf.fr)

The incident photon energy was set to 8.2 keV, *i.e.,* corresponding to a wavelength of *λ* = 1.516 nm. A 2D photon counting pixel detector (imXPAD D5) was used for the SWAXS (T) acquisitions. The scattering-vector modulus is defined as *q* = (4π/*λ*)·sin*θ*, where *θ* is half of the scattering angle and *λ* is the wavelength. The *sample-to-detector* distance (SDD = 31 cm) was used to cover a scattering-vector modulus q-range from 0.031 to 1.49 Å^-1^ *i.e.,*characteristic distances ranging from 0.42 to 20.5 nm. TILCs were sealed in home-made (copper brass-based) circular holders equipped with 50 microns-thick Kapton^®^ windows. An eight-position furnace was used to control the temperature of the sample between RT and 200 °C. The corrections and analysis of (primary) data were carried out using a Python-based (Jupyter notebooks) SAXS/WAXS platform available on the beamline. Silver behenate (AgBe, CAS number: [2489-05-6]) and Lanthanum hexaboride (LaB_6,_ CAS number [12008-21-8] were used as standards for the SAXS/WAXS *q*-range calibration. The intensity curves were scaled to the absolute intensities with the aid of a glassy carbon standard. The contribution of the empty cells was subtracted from the scattering intensity of the TILCs. 2D images were converted into 1D radial averages over the image centre to yield the scattered intensity I(q) *vs.* the scattering-vector modulus q.


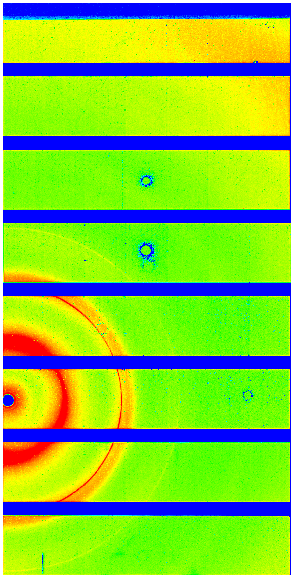


**Figure S25**: A SWAXS Synchrotron 2D pattern at 110 °C (left) and 1D patterns (right) at different temperatures for the [C_18_C_18_Im]^+^/Br^-^ TILC during a first cooling cycle.

**Table S8**: Data extracted from the 1D SWAXS diffusion profiles during the first cooling cycle of the A-CLIT [C_18_C_18_Im]^+^/Br^-^: interlamellar distance (d_001_), domain size, and number of lamellae per mesomorphic domain (ratio between domain size and d_001_).

| **T (°C)** | **d_001_ (Å)** | **ξ (Å)** | **ξ / d_001_** |
| --- | --- | --- | --- |
| 130 | 32.61 | 821.1 | ~ 25 |
| 120 | 33.49 | 771.6 | ~ 23 |
| 110 | 34.20 | 866.1 | ~ 25 |
| 100 | 35.21 | 1067.2 | ~ 30 |
| 80 | 38.22 | 855.4 | ~ 22 |


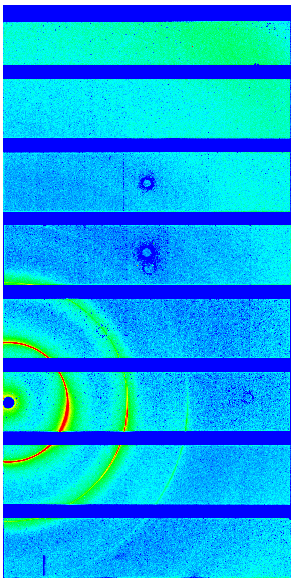


**Figure S26**: A SWAXS Synchrotron 2D pattern at 125 °C (left) and 1D patterns (right) at different temperatures for the [C_18_C_18_Im]^+^/I^-^ TILC during a first cooling cycle. The stars correspond to the residual Kapton^®^ scattering signal after subtraction.

**Table S9**: Data extracted from 1D SWAXS diffusion profiles during the first cooling cycle of the A-CLIT [C_18_C_18_Im]^+^/I^-^: interlamellar distance (d_001_), domain size, and number of lamellae per mesomorphic domain (ratio between domain size and d_001_).

| **T (°C)** | **d_001_ (Å)** | **ξ (Å)** | **ξ/ d_001_** |
| --- | --- | --- | --- |
| 140 | 31.03 | 1416.0 | ~ 46 |
| 135 | 31.29 | 1078.9 | ~ 34 |
| 130 | 31.56 | 1684.3 | ~ 53 |
| 125 | 31.92 | 1354.3 | ~ 42 |
| 120 | 32.11 | 1447.8 | ~ 45 |
| 115 | 32.50 | 1530.9 | ~ 47 |
| 110 | 33.18 | 994.3 | ~ 30 |
| 105 | 33.18 | 989.8 | ~ 30 |
| 100 | 33.58 | 1344.7 | ~ 40 |
| 90 | 34.52 | 982.8 | ~ 28 |
| 80 | 35.26 | 1349.6 | ~ 38 |


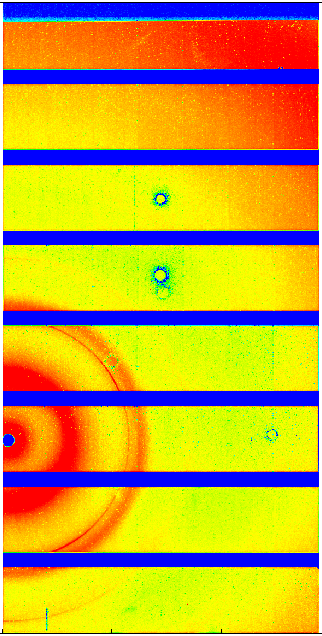


**Figure S27**: A SWAXS Synchrotron 2D pattern at 70 °C (left) and 1D patterns (right) at different temperatures for the [C_18_C_18_Im]^+^/[N(CN)_2_]⁻ TILC during the first cooling cycle.

**Table S10**: Data extracted from 1D SWAXS diffusion profiles during the first cooling cycle of the A-CLIT [C_18_C_18_Im]^+^/[N(CN)_2_]⁻ : interlamellar distance (d_001_), domain size, and number of lamellae per mesomorphic domain (ratio between domain size and d_001_).

| **T (°C)** | **d_001_ (Å)** | **ξ (Å)** | **ξ/ d_001_** |
| --- | --- | --- | --- |
| 80.0 | 33.55 | 1305.1 | ~ 39 |
| 70.0 | 34.58 | 1302.3 | ~ 38 |
| 65.0 | 35.15 | 1134.9 | ~ 32 |
| 62.5 | 35.25 | 1096.2 | ~ 31 |
| 60.0 | 35.70 | 955.8 | ~ 27 |
| 57.5 | 35.95 | 873.0 | ~ 24 |


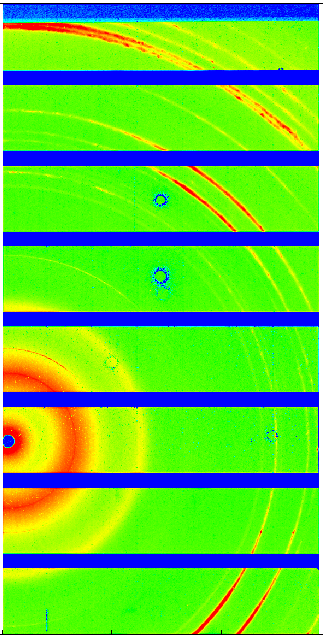


**Figure S28**: A SWAXS Synchrotron 2D pattern at 50 °C (left) and 1D patterns (right) at different temperatures for the [C_18_C_18_Im]^+^/[NTf₂]⁻ TILC during the first cooling cycle.

**Table S11**: Data extracted from 1D SWAXS diffusion profiles during the first cooling cycle of the A-CLIT [C_18_C_18_Im]^+^/[NTf₂]⁻: interlamellar distance (d_001_), domain size and number of lamellae per mesomorphic domain (ratio between domain size and d_001_).

| **T (°C)** | **d_001_ (Å)** | **ξ (Å)** | **ξ/ d_001_** |
| --- | --- | --- | --- |
| 150 | 28.16 | 56.0 | ~ 2 |
| 145 | 28.14 | 56.7 | ~ 2 |
| 140 | 28.17 | 57.3 | ~ 2 |
| 135 | 28.19 | 57.9 | ~ 2 |
| 130 | 28.22 | 58.7 | ~ 2 |
| 125 | 28.26 | 59.4 | ~ 2 |
| 120 | 28.32 | 60.1 | ~ 2 |
| 115 | 28.38 | 61.2 | ~ 2 |
| 110 | 28.44 | 62.1 | ~ 2 |
| 105 | 28.50 | 62.9 | ~ 2 |
| 100 | 28.58 | 64.0 | ~ 2 |
| 90 | 28.75 | 66.2 | ~ 2 |
| 80 | 28.94 | 68.5 | ~ 2 |
| 70 | 29.17 | 71.1 | ~ 2 |
| 65 | 29.27 | 72.5 | ~ 2 |
| 62,5 | 29.34 | 73.5 | ~ 3 |
| 60 | 29.42 | 74.7 | ~ 3 |
| 57,5 | 29.47 | 75.5 | ~ 3 |

The Bragg reflections are associated with a diffuse halo present at wide angles (from 4.60 to 4.66 Å between 75 and 130 °C). The values are taken as the maximum of the diffuse halos by fitting with a Gaussian function. As aliphatic (ionophobic) chains tend to organise themselves due to an ionophobic/ionophilic nanostructuring process, this distance corresponds to the average intermolecular characteristic distance between adjacent chains and typically varies around ca. 4.8 Å.^[[20]](#footnote-20)^ This organisation is largely controlled by the presence of van der Waals interactions between the aliphatic chains. The presence of a diffuse halo is related to the distribution of the characteristic distances between the *n*-alkyl chains, *i.e.,*to a certain degree of disorder, although they show a certain degree of organisation at short distances. The SmA_d_ mesophases of the TILCs are consisting in dynamically self-assembling lamellae composed of *ca.* 1nm-thick ionic sub-layers alternating with melted *n*-alkyl chains ones.

*SWAXS(T) experiments at the* *[ID31](https://www.esrf.fr/UsersAndScience/Experiments/StructMaterials/ID31) SAXS/WAXS beamline at* [*ESRF*](https://www.esrf.fr)

*In situ* and *operando* measurements were performed to obtain direct structure/ionic transport correlations. This specific configuration presents some differences with respect to the configuration used for the previous SWAXS(T) measurements: **i)** the reduction of the distance between the electrodes (from 1 mm to 4 µm), **ii)** different anchoring surfaces for the TILCs (electrodes made of indium tin oxide (ITO) instead of Kapton^®^ thin films for SAXS/WAXS (SWAXS) measurements and gold or platinum electrodes for EIS measurements), and **iii)** a larger sample area penetrated by the incident X-ray beam (2 cm instead of 1 mm, *i.e.,*the electrodes are parallel to the incident beam). The orientation of mesophases displayed by TILCs depends on the anchoring energies^[[21]](#footnote-21)-^^[[22]](#footnote-22)^ between these functional liquid crystals and the used substrates (e.g., ITO or gold). The anchoring energy corresponds to the free energy cost to change the orientation of the TILCs to a different direction than their initial orientation.^[[23]](#footnote-23)^

The monochromatic X-ray beam energy was 77 keV (*i.e.,*a wavelength 𝜆 = 0.161018 Å). This experimental configuration covers a range of scattering vector modulus q from 0.08 to 2.82 Å^-1^ (*i.e.*, to probe characteristic distances d from 7.57 to 0.22 nm). The SWAXS data were acquired with a 2D Dectris Pilatus CdTe 2M detector. The SDD was *ca.* 4.33 m. The LC cell was connected to a potentiostat (SP-200 from BioLogic) to perform EIS measurements (from 7 MHz to 200 mHz with a voltage amplitude of 10 mV while having 11 points per decade). An open-circuit voltage (OCV) measurement is taken before each experimental run. **Figure S27** shows a synoptic scheme of the experimental setup. In the experimental setup, the incident X-ray beam converges at the centre of the measurement liquid-crystal (LC) cell in contact with a home-made furnace. This furnace is inserted into a double solenoid that applies an external magnetic field adjustable between 0 and 1 Tesla. Through-plane (configuration for EIS measurements) commercially available (Instec Inc., Boulder, CO, USA) LC cells were used.


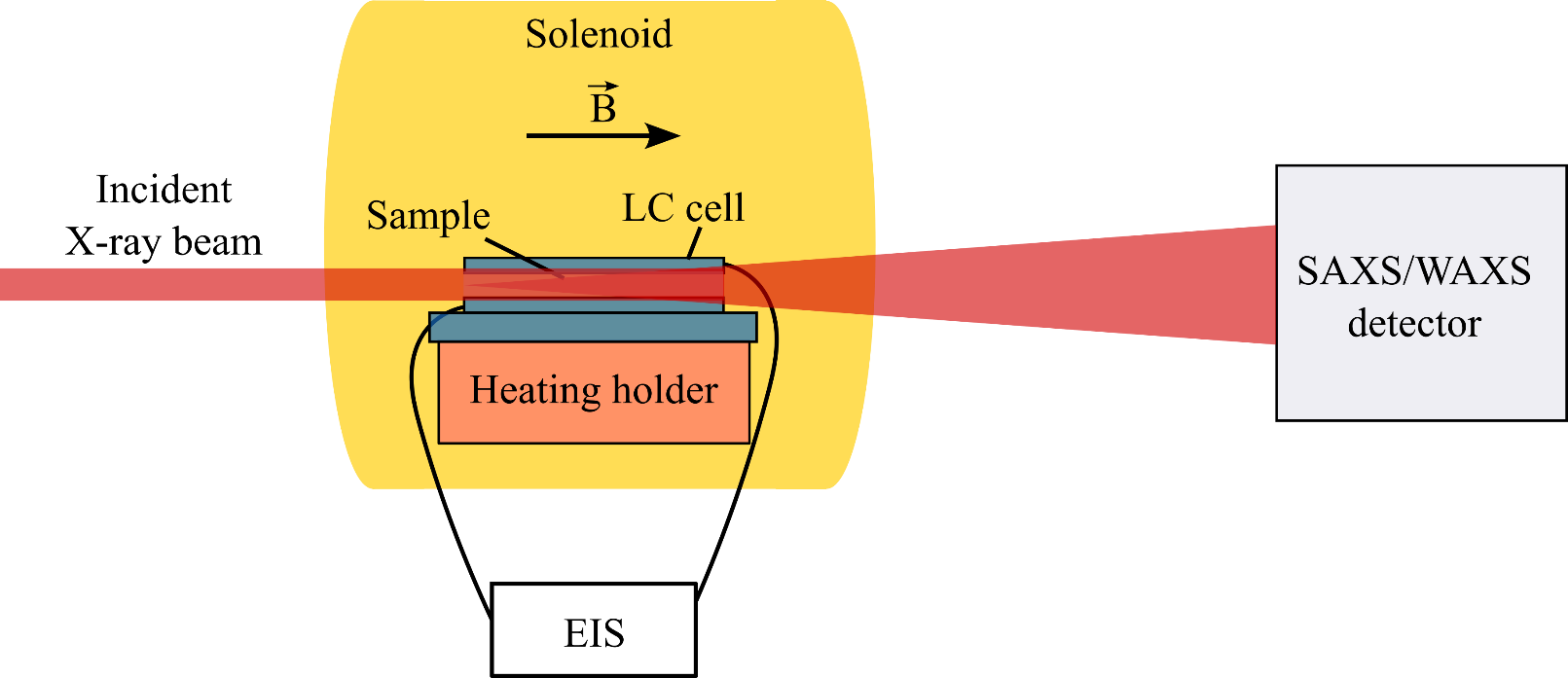


**Figure S29**: Synoptic scheme of the experimental setup on the [ID31](https://www.esrf.fr/UsersAndScience/Experiments/StructMaterials/ID31)@[ESRF](https://www.esrf.fr) beamline.

The experimental protocol consists of a heating scan of the sample from 25 to 140 °C using 10 °C steps. A ten-minute thermalisation period between each step ensures reproducible experimental conditions, followed by (2D) SWAXS image acquisition (typical duration: 3 s) and EIS measurements (typical duration: 4 to 6 min). Heating and cooling rates were set at 3 °C·min^-1^. When the temperature of 140 °C was reached at the end of a 1^st^ heating scan, an external magnetic field of 1 T was progressively applied (incrementally with steps of 0.2 T). The sample was then cooled to 50 °C before undergoing a 2^nd^ heating scan to 140 °C; the last two cycles were performed while maintaining a magnetic field of 1 T. At 140 °C, the procedure was reversed: the magnetic field was gradually reduced from 1 to 0 T. Finally, the sample was subjected to a 2^nd^ cooling scan in the absence of a magnetic field in order to compare the cycles with (1^st^ cooling scan) and without (2^nd^ cooling scan) an external magnetic field. **Figure S30** gives a synoptic summary of the different stages of the experimental protocol and of the temperature cycles imposed on the sample.


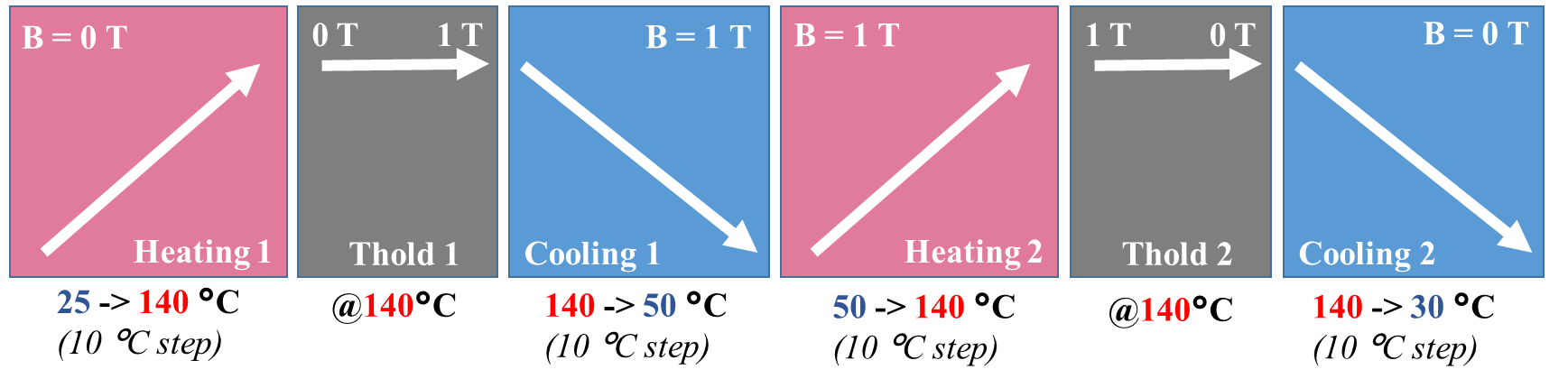


**Figure** **S30**: Synopsis of the experimental protocol during *in situ*/*operando* experiments performed on the [ID31](https://www.esrf.fr/UsersAndScience/Experiments/StructMaterials/ID31)@[ESRF](https://www.esrf.fr) beamline.

Data treatment: The 2D images were processed using two types of radial integrations (using pyFAI packages^[[24]](#footnote-24)^) to obtain 1D profiles: out-of-plane (*to obtain information on the supramolecular organisation in the thickness*) and in-plane (*to obtain information on the supramolecular organisation in the plane*). These integrations were obtained by applying two different masks and are shown in **Figure S29**. The comparison between these two integrations provides information about the structural anisotropy of the sample. In the same figure, the incident X-ray beam is shown with a rectangular cross section of 5 µm height and 15 µm width.


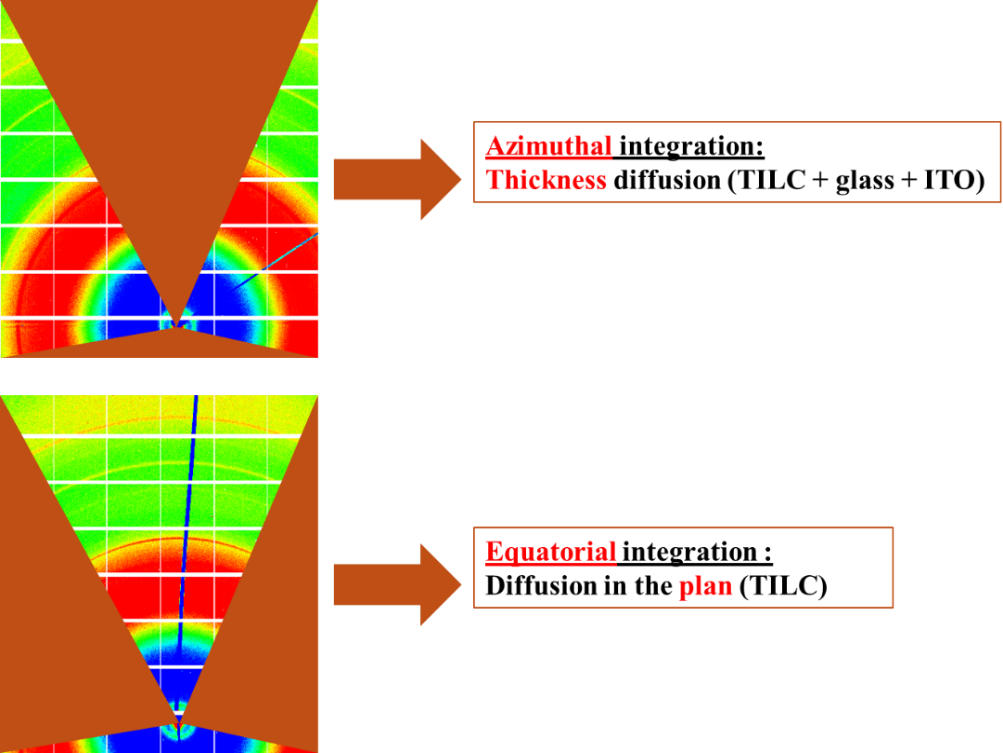


**
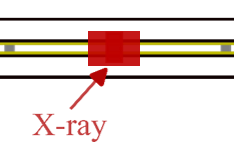
**

**Figure S31:** Masks applied to 2D images for obtaining two radial integrations: in the thickness and in the plane of the thin LC cell, and cross-section of the incident X-ray beam on the LC cell.

With an inter-electrode distance of 4 µm and an incident beam height of 5 µm, the incident beam also scatters onto the ITO electrodes and glass substrates. To account for these additional scattering, these contributions were eliminated by the appropriate subtraction of an empty cell. **Figure S32** shows an example of subtracting the empty cell from the raw 1D scattering profile of the [C_18_C_18_Im]^+^/[N(CN)_2_]⁻ TILC at 60°C during the first cooling cycle. A low intensity contribution related to the ITO electrodes is observed at q= 0.19 Å^-1^ and another higher intensity and more diffuse one at q= 1.73 Å^-1^ related to the diffusion of the glass substrates.

**Figure S32**: Superimposition of raw 1D scattering profiles of the [C_18_C_18_Im]^+^/[N(CN)_2_]⁻ TILC at 60 °C during the cooling cycle (black), the empty cell (red), and the subtracted profile (brown).

*Calculation of the correlation lengths (i.e.,average size of the dynamic domains)*

Scherrer equation for crystallite size determination^[[25]](#footnote-25)^ was adapted to extract the correlation lengths of the TILCs. This reflects the long-range order present in these liquid crystalline ionic conductors. Correlation lengths were extracted as a function of the temperature. The following equation, adapted from the general Scherrer equation, was used to extract these values:

$$Correlation Length (nm)=\frac{K\cdot2\pi}{\Delta q}$$

in which *K*= 0.9 is a dimensionless shape factor, and $\Delta q$represents the full-width at half-maximum (FWHM) of the peak in nm^-1^ (*q* = (4)·sin* * , where 2*θ* is the scattering angle).

**11. Level of *n*-alkyl chain interdigitation**

To determine whether a SmA mesophase has a monolayer, interdigitated or bilayer lamellar structure, the values of the interlamellar distances d_001_ must be compared with those of the molecular lengths of the A-CLITs in their fully-extended (*trans*) conformation (L_calc_)^[[26]](#footnote-26)^. L_calc_ was estimated using Chem3D (version 15.1) modelling software from CambridgeSoft. MM2 structure minimisation was used to obtain the fully extended (*trans*) conformations of the A-CLITs. **Figures S33-35** show the molecular models obtained for the A-CLITs [C_18_C_18_Im]^+^/Br^-^, [C_18_C_18_Im]^+^/I^-^ and [C_18_C_18_Im]^+^/[N(CN)_2_]⁻ with L_calc_ of 48.35, 48.35, and 48.45 Å respectively.


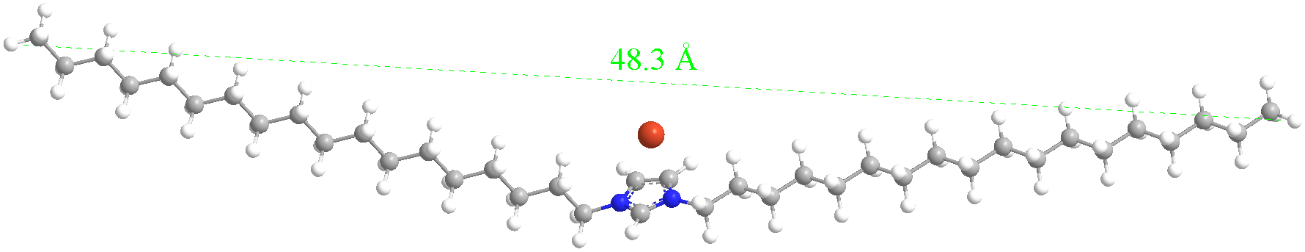


**Figure S33**: Minimised structure (MM2) of the A-CLIT [C_18_C_18_Im]^+^/Br^-^, in fully-extended *(trans*) configuration. The L_calc_ is 48.35 Å.


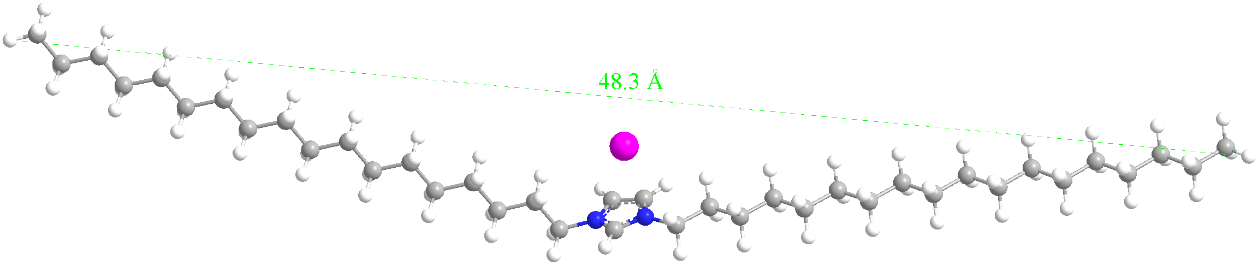


**Figure S34**: Minimised structure (MM2) of the A-CLIT [C_18_C_18_Im]^+^/I^-^, in fully-extended *(trans*) configuration. The L_calc_ is 48.35 Å.

*
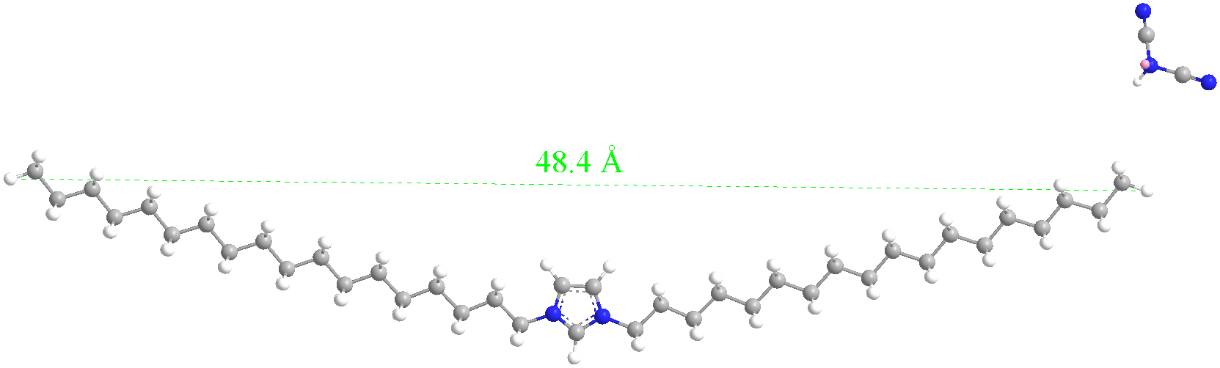
*

**Figure S35**: Minimised structure (MM2) of the A-CLIT [C_18_C_18_Im]^+^/[N(CN)_2_]⁻, in fully-extended *(trans*) configuration. The L_calc_ is 48.45 Å.

**Table 12** shows all the values for d_001_ (over the temperature range of the SmA mesophases), L_calc_. These results reveal a partially interdigitated lamellar organisation (SmA_d_) within the mesophases of these A-CLITs, an intermediate situation in between a monolayer (SmA_1_, d_001_≅L_calc_/2)and a bilayer (SmA_2_, d_001_≅L_calc_) lamellar organization.

**Table 12**: Table comparing the characteristic interlamellar distances with the molecular length of the A-CLITs.

| **Anion of the A-CLIT** | **d_001_ (Å)** | **L_calc_ (Å)** |
| --- | --- | --- |
| Br^-^ | 32.61 < d_001_ < 38.22 | 48.35 |
| I^-^ | 31.03 < d_001_ < 35.26 | 48.35 |
| [N(CN)_2_]⁻ | 33.55 < d_001_ < 35.95 | 48.45 |

# **12. Nanoconfinement as a function of the nature of the anions**

The ratio between the thickness of the ionic sub-layer and the average ionic diameter is used to approximate the maximum number of anions that can theoretically accommodate the thickness of the ionic sublayer. This ratio is called C_2D_. It provides an estimate of the ionic confinement within TILCs.

**Figure S36** shows the evolution of the C_2D_ parameter as a function of temperature. It should first be noted that the ionic nanoconfinement is justified as the anions have a close environment of electrostatic interactions within the polar sublayers with an average thickness of *ca.* 1 nm. A first observation is that the I^-^ and Br^-^ anions show the least pronounced confinement within the anionic sublayers while the [C_18_C_18_Im]^+^/[N(CN)_2_]⁻ shows the most pronounced confinement of its anions within the ionic sublayer. In conclusion, the theoretical number of anions that can be confined in the direction transverse to the ionic (polar) sublayer is a maximum of three in the case of TILCs based on spherical halide anions and a maximum of two in the case of those based on ovoid anions. These results underline that the specific nature of the anion plays an important role in the structuring of the ionic and aliphatic sublayers.

**
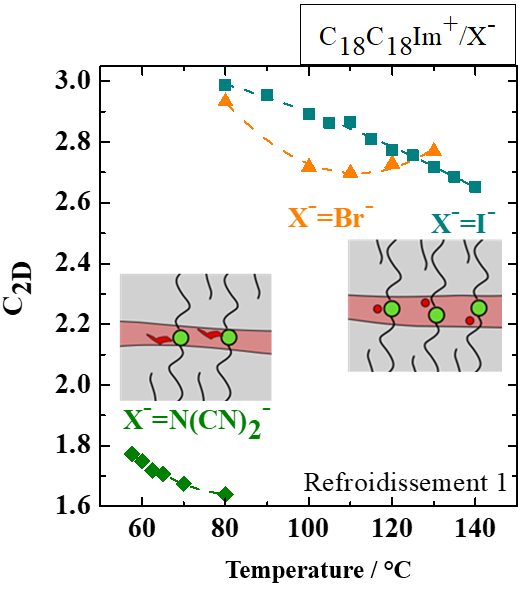
**

**First Cooling**


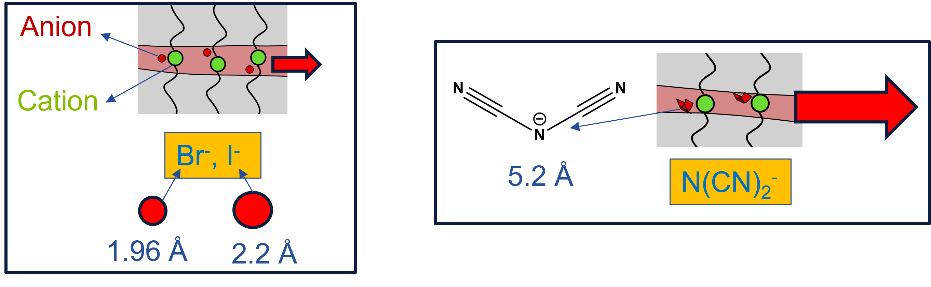


**Figure S36.** Evolution of the C_2D_ ratio (between d_ion_ and anion diameter), which approximates the number of anions that can theoretically be accommodated in the direction transverse to the ionic sublayer, as a function of temperature. The dotted lines are guidelines for eyes (i.e. *to indicate a trend*) and are obtained by fitting the data with second-degree polynomial functions. The inset plots synoptically illustrate the nanoconfinement within the ionic (polar) sublayer of SmA of the anions [N(CN)_2_]⁻ (left) and halides (Br^-^, I^-^) (right). The circular shapes in green and red represent the polar group of the cation Im^+^ and the halide anions Br^-^ and I^-^, respectively. The ovoid shapes (in red) represent the [N(CN)_2_]⁻ anions.

# **13. Atomistic simulations**


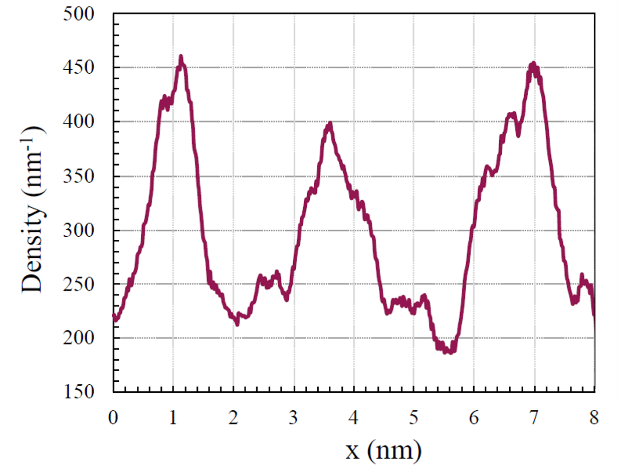
All-atom theoretical simulations^[[27]](#footnote-27)^ gives the ability to calculate quantities that are not, or are very difficult to access by experiment like the volume variation of the electronic density (see example in **Figure S37**). Atomic simulations were used to calculate structural parameters. At the atomistic level, every atom in the system is represented explicitly in the force field.

**Figure S37**: Atomistic simulation of the *through-plane* electronic density for the [C_18_C_18_Im]^+^/[N(CN)_2_]⁻ TILC.

A CL&Pol^[[28]](#footnote-28)^ polarizable force field was used for all-atom molecular simulations with molecular compounds, and the molecular dynamics trajectories were produced with the OpenMM molecular dynamics software^[[29]](#footnote-29)^ on GPU processors. Having explicit polarisation provides a better agreement of structural and dynamic/transport properties in ionic fluids, whereas previous-generation fixed-charge models systematically predicted too slow dynamic in comparison to experiment. The CL&Pol force field uses induced Drude dipoles to represent polarisation. The TILCs were modelled here in periodic boxes containing 500 ion pairs, with different sizes according to the particular ions (see example in **Figure S39**).

**Figure S38** gives a view of the spatial distribution functions associated with the atoms of the [C_18_C_18_Im]^+^ cation at 80 °C. The presence of a high anionic density region localized around the imidazolium head-group indicates the strong association between ionic moieties and a nanophase segregation of *n*-alkyl chains.


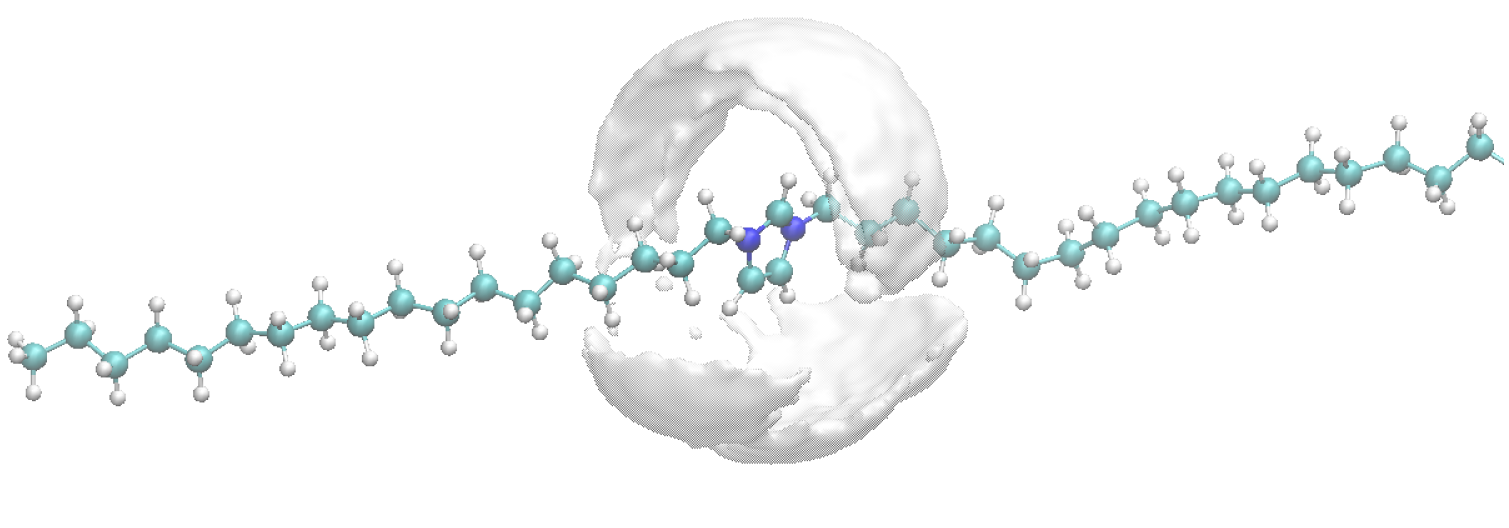


**Figure S38**. Representation of the spatial distribution functions of the atoms (N in dark blue, C in turquoise, and H in white) surrounding the cation [C_18_C_18_Im]^+^ at 80 °C.

| [C_18_C_18_Im]^+^**/**[N(CN)_2_]⁻**, 80 °C**  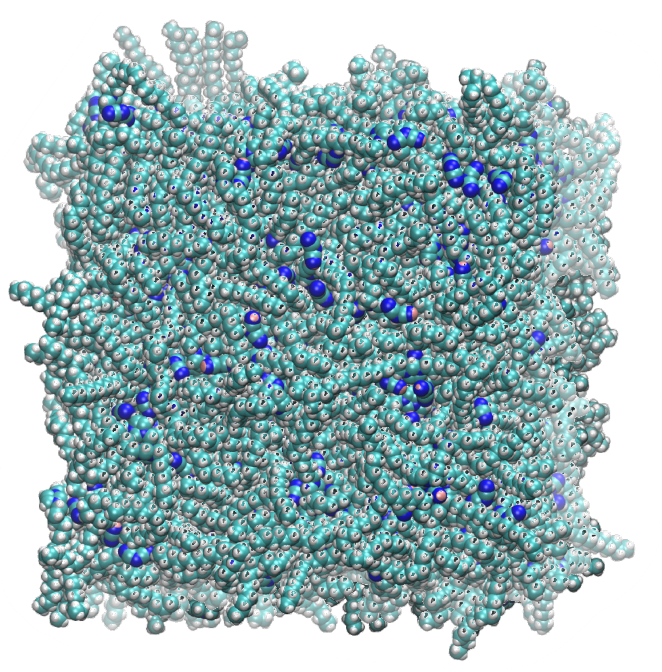 | [C_18_C_18_Im]^+^/[NTf₂]⁻**, 80 °C**  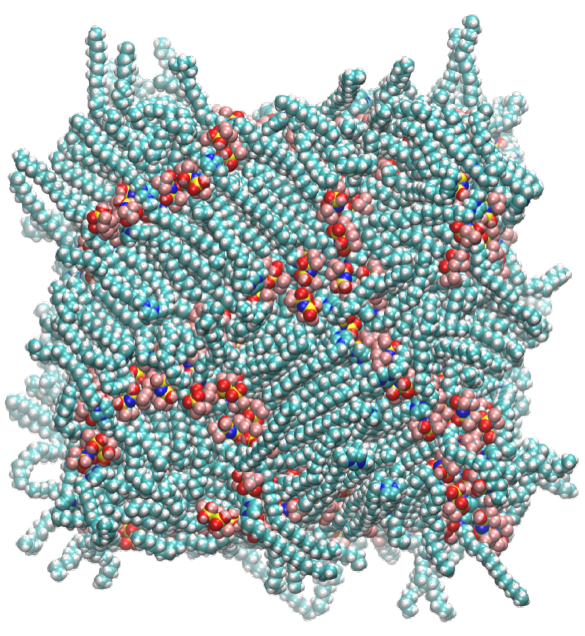 |
| --- | --- |
| **OpenMM with GPU processing**  **Speed: 21.9 ns/day**  Ion pairs: 500 (84501 particles)  Box side: 8.51 nm  Average density: 862.8 kg·m^-3^ | **OpenMM with GPU processing**  **Speed: 21.3 ns/day**  Ion pairs: 500 (94501 particles)  Box side: 8.91 nm  Average density: 990 kg·m^-3^ |
|  | [C_18_C_18_Im]^+^**/**[NTf₂]⁻**, 65 °C**  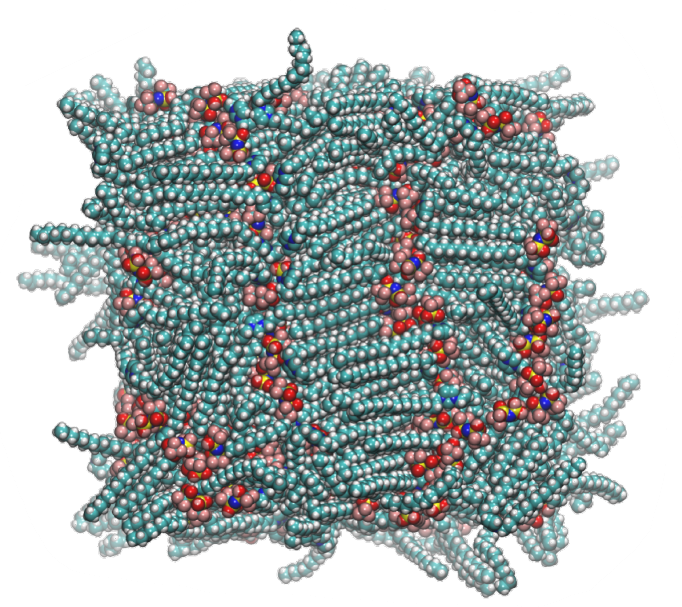 |
|  | **OpenMM with GPU processing**  **Speed: 20.8 ns/day**  Ion pairs: 500 (94501 particles)  Box size: 8.765 nm  Average density: 1050 kg·m^-3^ |

**Figure S39**. Example of computational boxes containing 500 ion pairs.

It is computationally expensive for large systems, and it can take a substantial time for a system to relax from a random configuration to a liquid crystalline phase. For a box volume of ca. 10^3^ nm^3^, we have ca. 10^5^ particles and a productivity of 20 ns/day, hence the difficulty to analyse long-range phenomena. For these reasons, coarse-grained simulations were also used.

**Theoretical molecular volumes**

# One of the key quantities obtained from these simulations is the molecular volume (V_mol_) in Å^3^. The molecular volume is defined as the average volume occupied by a pair of ions and is related to the compactness and spatial arrangement of the molecules in a TILC at a given temperature. Molecular volume values (expressed in Å^3^) were therefore calculated theoretically using the following relation:

#

$$\begin{aligned} V_{mol}\left( T \right)=\frac{Box_{side}^{3}\left( T \right)}{Paire d^{'}ions} \# \end{aligned}$$

# **Table S13** shows all results obtained from these simulations: the values calculated for the structural factors (lateral dimension of a box, molecular volume, density) at different temperatures.

**Table S13**. Results of the atomic modelling (box side dimension, molecular volume and simulation box density) for the [C_18_C_18_Im]^+^/Br^-^, [C_18_C_18_Im]^+^/I^-^ and [C_18_C_18_Im]^+^/[N(CN)_2_]⁻ TILCs at different temperatures.

| **Anion** | **T(°C)** | **Box_side_ (Å)** | **Number of ion pairs** | **Molecular Volume (Å^3^)** | **Density (g/mL)** |
| --- | --- | --- | --- | --- | --- |
| Br^-^ | 130 | 74.77 | 500 | 1278.2 | 0.849 |
|  | 120 | 75.96 | 500 | 1256.8 | 0.864 |
|  | 110 | 85.23 | 500 | 1238.4 | 0.877 |
|  | 100 | 84.88 | 500 | 1223.0 | 0.888 |
|  | 80 | 84.37 | 500 | 1201.2 | 0.904 |
| I^-^ | 150 | 86.93 | 500 | 1313.8 | 0.886 |
|  | 140 | 86.73 | 500 | 1304.6 | 0.892 |
|  | 135 | 86.62 | 500 | 1300.0 | 0.895 |
|  | 130 | 86.51 | 500 | 1295.0 | 0.898 |
|  | 125 | 86.40 | 500 | 1290.1 | 0.902 |
|  | 120 | 86.28 | 500 | 1284.7 | 0.906 |
|  | 115 | 86.16 | 500 | 1279.2 | 0.910 |
|  | 110 | 86.03 | 500 | 1273.6 | 0.914 |
|  | 105 | 85.90 | 500 | 1267.8 | 0.918 |
|  | 100 | 85.77 | 500 | 1261.8 | 0.922 |
|  | 90 | 85.48 | 500 | 1249.3 | 0.931 |
|  | 80 | 85.18 | 500 | 1236.2 | 0.942 |
| [N(CN)_2_]⁻ | 80 | 84.74 | 500 | 1163.8 | 0.913 |
|  | 70 | 83.22 | 500 | 1152.7 | 0.922 |
|  | 65 | 83.12 | 500 | 1148.5 | 0.925 |
|  | 62.5 | 83.07 | 500 | 1146.7 | 0.927 |
|  | 60 | 83.03 | 500 | 1145.1 | 0.929 |
|  | 57.5 | 83.00 | 500 | 1143.7 | 0.930 |

Molecular volume values (V_mol_) are generally measured experimentally, in particular using dilatometry techniques. However, these measurements often require relatively large amounts of sample. Some authors have therefore used an empirical relationship (see the following relation) to approximate the V_mol_ values of TILCs^[[30]](#footnote-30)^:

$$\begin{aligned} V_{mol} = \frac{M}{0.6022}f \# \end{aligned}$$

in which $f = 0.9813+7.474\cdot{10}^{-4} T$ is an experimentally obtained thermal correction factor, *T* is the temperature in °C, and M is the molar mass in g·mol^-1^.

This equation is based on the experimental finding that the expansion of the volume of TLCs with increasing temperature in their mesophase is mainly due to the expansion of their *n*-alkyl chains, while the volume of rigid parts, such as aromatic rings, is less dependent on temperature^[[31]](#footnote-31)^. In a study by Park *et al.^[[32]](#footnote-32)^,* the validity of this equation was verified by comparing the experimental molecular volumes of 62 salts, including 38 ionic liquids and 24 TILCs^[[33]](#footnote-33)^, with the values calculated using this empirical equation.

The results of this study showed that the proposed relation tended to overestimate molecular volume values. However, this overestimation was relatively small for TILCs with a higher proportion of *n*-alkyl chains and, to a lesser extent, aromatic groups. On the other hand, more significant deviations were observed for TILCs containing a significant proportion of halogen atoms or transition metals, particularly for TILCs containing I^-^ or

[NTf₂]⁻ anions.

**Simulated vs. empirical molecular volume**

It is interesting to compare the values obtained by simulation with those obtained using this empirical formula. **Figure S40** superimposes the values obtained using the empirical equation and the results of the atomic simulations. For the [C_18_C_18_Im]^+^/Br^-^, [C_18_C_18_Im]^+^/I^-^ and [C_18_C_18_Im]^+^/[N(CN)_2_]⁻ TILCs, the use of the empirical formula systematically leads to an underestimation of the simulated molecular volume values, with mean deviations of 6.94%, 2.81% and 4.76%, respectively. As these deviations are relatively small, it is possible to use this empirical formula to obtain an approximation of the order of magnitude of the molecular volumes. In the following results, only the simulated molecular volume values are used to calculate other structural parameters.

**
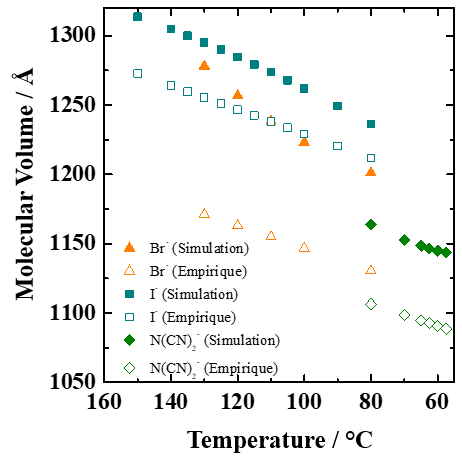
**

**Figure S40**. Calculated Molecular volumes compared with those obtained by atomic simulation for the [C_18_C_18_Im]^+^/Br^-^, [C_18_C_18_Im]^+^/I^-^ and [C_18_C_18_Im]^+^/[N(CN)_2_]⁻ TILCs.

All results and the values of the deviations at each temperature are shown in **Tables S14-16**.

**Table S14**. Comparison of molecular volume values obtained by simulation vs. empirically and errors between these two values for the [C_18_C_18_Im]^+^/Br^-^ TILC as a function of temperature.

| **TILC of Br^-^** | **V_mol_ by simulation (Å^3^)** | **Empirical V_mol_ (Å^3^)** | **Deviation (%)** |
| --- | --- | --- | --- |
| 130 | 1278.2 | 1171.1 | 8.38 |
| 120 | 1256.8 | 1163.0 | 7.46 |
| 110 | 1238.4 | 1154.9 | 6.74 |
| 100 | 1223.0 | 1146.8 | 6.23 |
| 80 | 1201.2 | 1130.5 | 5.88 |

**Table S15**. Comparison of molecular volume values obtained by simulation vs. empirically and errors between these two values for the [C_18_C_18_Im]^+^/[N(CN)_2_]⁻ TILC as a function of temperature.

| **TILC of N(CN)_2_^-^** | **V_mol_ by simulation (Å^3^)** | **Empirical V_mol_ (Å^3^)** | **Deviation (%)** |
| --- | --- | --- | --- |
| 80 | 1163.8 | 1106.6 | 4.92 |
| 70 | 1152.7 | 1098.6 | 4.69 |
| 65 | 1148.5 | 1094.7 | 4.68 |
| 62.5 | 1146.7 | 1092.7 | 4.71 |
| 60 | 1145.1 | 1090.7 | 4.75 |
| 57 | 1143.7 | 1088.7 | 4.81 |

**Table S16**. Comparison of molecular volume values obtained by simulation vs. empirically and errors between these two values for the [C_18_C_18_Im]^+^/I^-^ TILC as a function of temperature.

| **TILC of I^-^** | **V_mol_ by simulation (Å^3^)** | **Empirical V_mol_ (Å^3^)** | **Deviation (%)** |
| --- | --- | --- | --- |
| 140 | 1304.6 | 1264.0 | 3.12 |
| 135 | 1300.0 | 1259.6 | 2.73 |
| 130 | 1295.0 | 1255.3 | 2.70 |
| 125 | 1290.1 | 1250.9 | 2.62 |
| 120 | 1284.7 | 1246.6 | 2.12 |
| 115 | 1279.2 | 1242.2 | 1.55 |
| 110 | 1273.6 | 1237.9 | 0.91 |
| 105 | 1267.8 | 1233.5 | 0.22 |
| 100 | 1261.8 | 1229.2 | 2.58 |
| 90 | 1249.3 | 1220.5 | 2.31 |
| 80 | 1236.2 | 1211.8 | 1.97 |

**Transverse surfaces (A_mol_)**

Using the simulated molecular volume values, the cross-sectional area values occupied by an elementary molecular assembly within a smectic layer (A_mol_ in Å^2^) can be calculated. The mathematical expression is given by following relation:

$$\begin{aligned} A_{\mathrm{mol}}= N\frac{V_{\mathrm{mol}}}{d_{001}} \# \end{aligned}$$

in which N = 1 for a monolayer lamella and N = 2 for a bilayer lamella.

**The thickness of an ionic/hydrophilic sub-layer (d_ion_)** can be estimated using an empirical formula based on the partial additivity of the molar volume The d_ion_ thickness of an ionic sublayer is given by the following relation^[[34]](#footnote-34)^:

$$\begin{aligned} d_{ion}\left( T \right)=\frac{2\left( V_{mol}\left( T \right)-V_{ch}\left( T \right) \right)}{A_{mol}\left( T \right)}= \frac{2\left( V_{mol}\left( T \right)-\left[ \boldsymbol{34}V_{CH_{2}}\left( T \right)+\boldsymbol{2}\Delta V_{CH_{3}}\left( T \right) \right] \right)}{A_{mol}\left( T \right)} \# \end{aligned}$$

in which V_ch_(T) = 34V_CH2_(T)+2ΔV_CH3_(T) as each ion pair of TILCs has two C_18_ aliphatic chains with 34 CH_2_ groups and a total of two CH_3_ groups, ΔV_CH3_(T) represents the volume difference between a CH_3_ group and a CH_2_ group with $\Delta V_{CH_{3}}\left( T \right)=27.14+0.01713\cdot T+0.0004181\cdot T^{2} (in Å^{3})$, and T is in °C. Similarly, the thickness of the aliphatic sub-layers, d_ch_, can be calculated by subtracting the thickness of the ionic sub-layers (d_ion_) from the interlamellar distance d_001_ (d_ch_ = d_001_-d_ion_).

# **14. Coarse-grained force fields**

With coarse-grained force fields, molecules are represented by pseudo-atoms with force fields in building blocks with chemical specificity. The simulations are faster (10^2^-10^3^ increase in speed). By reducing the number of degrees of freedom we can achieve longer simulation times. The Martini 3^[[35]](#footnote-35)^ coarse-grained force field was used for coarse-grained molecular dynamics simulations using the GROMACS software^[95]^.

**Figure S41** shows the atomic arrangement in space of 500 ion pairs of the [C_18_C_18_Im]^+^/[NTf₂]⁻ TILC (88,501 particles in total) in a simulation box environment. The modelling of the scattered intensity as a function of the modulus of the wave vector was carried out using the CL&Pol force field, at a temperature of 80 °C. Thanks to this simulation, it was possible to determine the dimensions of the simulated cube (8.51 nm on one side and density 0.86 kg·m^-3^). These parameters are essential for the accurate and realistic reproduction of the experimental conditions for the atomic simulation.


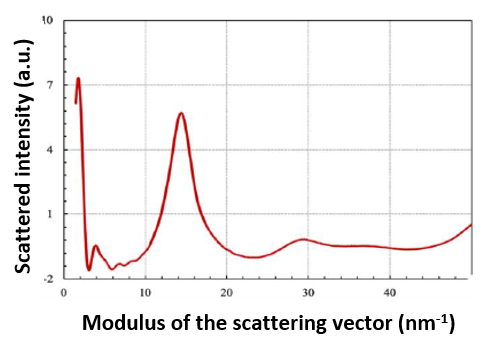


**Figure S41**. Representation of the atomic simulation of 500 ion pairs of the [C_18_C_18_Im]^+^/[NTf₂]⁻ TILC within a cubic simulation box (left) and representation of the evolution of the scattered intensity as a function of the modulus of the scattering vector (right) using atomic simulation with CL&Pol force fields at a given temperature of 80 °C.

**Self-diffusion coefficient.**

The self-diffusion coefficients were calculated using coarse-grained simulations. **Figure S42** shows the evolution of the self-diffusion coefficients of the [C_18_C_18_Im]^+^ cation and the N(CN)_2_^-^ anion between 25 and 145 °C as a function of temperature. These results show that the self-diffusion coefficients for the cation increase from 0.15·10^-5^ to 1.00·10^-5^ cm^2^·s^-1^ from 25 to 145 °C, while those for the anion increase from 0.73·10^-5^ to 4.39·10^-5^ cm^2^·s^-1^ over the same temperature range. At 25 °C, the self-diffusion values for the anion are 5 times higher than those for the cation while they are 4 times higher at 85 °C. These simulations therefore show that the diffusion process of the anion is between 4 and 5 times greater than that of the cation. *These trends confirm that the diffusion species in these TILCs are predominantly anionic*.

**Figure S42.** Evolution of the self-diffusion coefficients obtained by coarse-graining simulation for the [C_18_C_18_Im]^+^/[N(CN)_2_]⁻ TILC as a function of temperature.

# **15. Ion transport properties as probed by Electrochemical Impedance Spectroscopy (EIS)**

The ion transport properties of TILCs are intrinsically linked to a complex combination of factors involving chemical composition and dynamic hierarchical self-assembly. The nature of the cation and anion plays an important role, as does the viscosity of the electrolyte and the specific interactions that form between ions within the liquid crystalline self-assemblies (coordination *vs.* dissociation of ions)^[[36]](#footnote-36)^. The unique architecture of TILCSs allows for ion nanoconfinement, a critical feature that directly affects ion transport. Dynamic mosaicity (associated with the coexistence of mesomorphic domains with different preferred orientations within a mesophase) also affects ion transport by influencing the way ions move through these domains. Given this multitude of interactions and properties, it is imperative to understand the links between each parameter in order to enhance our understanding of ion transport in TILCs.

Depending on the dimensionality of the TILC mesophase, it may exhibit more or less pronounced anisotropy (*e.g.*, for nematic, columnar and smectic mesophases) or lack of anisotropy (*e.g.,* for cubic bicontinuous mesophases) in its transport properties. Within a smectic mesophase, ion transport is anisotropic, 2D by nature, which means that its ion transport properties considerably vary from one measurement direction to another (perpendicular to it). For this reason, ionic conductivity measurements should be carried out in two spatial directions to more accurately reflect the ionic transport properties in smectic mesophases. The anchoring of the molecules to the electrodes has a strong influence on the orientation of the TILC molecules and the size of the mesomorphic domains. Experimentally, the anisotropy of the ionic conductivity can be studied by measuring the transport properties by EIS using two measurement configurations: Through-Plane (TP) vs. In-Plane (IP).

Impedance (Z(ω) = U(ω)/I(ω)) is a generalisation of the measurement of electrical resistance by Ohm's law (R = U/I), where U is the voltage and I is the current. The operating principle of EIS is based on the application of a low-amplitude sinusoidal voltage (typically a few tens of millivolts to maintain a linear intensity response) to a symmetrical electrochemical cell^[[37]](#footnote-37)^. The phase shift and amplitude between the input and output signals are used to determine the imaginary Im(Z) and real Re(Z) parts of the impedance. These impedance values can be plotted on a Bode diagram (by plotting the modulus and phase shift of the impedance as a function of frequency) or an electrochemical Nyquist diagram (by plotting -Im(Z) as a function of Re(Z)).

Phenomenological models consisting of equivalent circuits are representations of the complex electrochemical behaviour of the system, allowing experimental data to be fitted to extract electrical parameters such as resistances, inductances or capacitances. **Figure S43** shows the Nyquist diagram obtained for an organic electrolyte between two blocking electrodes and the equivalent circuit used to model its electrochemical behaviour.


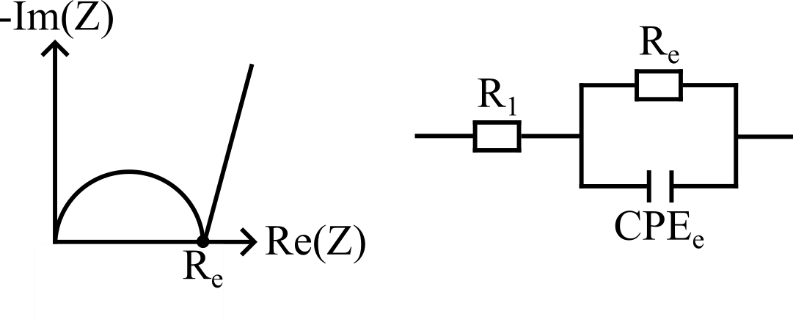


**Figure S43**. Complex plane representation (electrochemical Nyquist diagram) and phenomenological equivalent circuit of an electrochemical cell with ion-blocking electrodes.

The equivalent circuit consists of a resistor R_1_ in series with another resistor R_e_ in parallel with a constant phase element (CPE). A CPE is used to model the frequency behaviour (angular frequency ω) of an imperfect capacitor: its impedance is written as Z(ω)=1/C(jω)^n^ where n is a parameter between 0 and 1. Physically, the parameter n gives an indication of the homogeneity within the system, where 0 corresponds to an inhomogeneous system and 1 to a perfectly homogeneous system.

Knowing that the equivalent impedance of impedances in series is the sum of the impedances and that the equivalent impedance of impedances in parallel is the sum of the admittances, the impedance of the electrolyte Z_e_(ω) is written according to the following relation. This impedance is represented on the Nyquist diagram by the formation of a semicircle. This resistance R_e_ is obtained experimentally by fitting the semicircle or by taking the maximum point of the imaginary part or by extrapolating the vertical part, which represents the capacitive response at the interfaces.

$$Z_{e}\left( \omega\right)=\frac{R_{e}}{1+R_{e}{C_{e}\left( j\omega\right)}^{n}}$$

The impedance of the whole system is Z_e_(ω) + R_1_. The resistance R_1_ corresponds to the resistive part due to the measurement systems (analyzer, cables, electrodes), and the resistance R_e_ is the resistance of the electrolyte. From the value of R_e_, the ionic conductivity (σ) of the electrolyte between two blocking electrodes is obtained using the following relation. Once a phenomenological equivalent circuit has been chosen to model the experimental data, the ionic conductivity values (expressed in S·cm^-1^) can be calculated using the following relation:

$$\begin{aligned} \sigma=\frac{e}{R_{e}\cdot S} \# \end{aligned}$$

in which R_e_ is the resistance of the electrolyte obtained from the Nyquist diagram (measured in ohms), e represents the gap separating the electrodes (in this this study a 50 µm-thick PTFE^®^ spacer is used for TP-EIS) and S accounts for the electrode surface area encompassing the electric field lines between the two electrodes (in cm^2^).

**Measurement of ion transport properties in the Through-Plane (TP) configuration.**

The first (transverse) configuration, called "Through Plane" (TP), involves measuring the electric current through the measurement cell in a direction perpendicular to the surface of the electrodes. This approach allows the ion transport characteristics to be studied through the thickness of the cell. In the TP configuration, impedance measurements have been carried out in symmetrical cells where the electrolyte is placed between two ion-blocking electrodes made of gold or ITO for liquid crystal cells.

The EIS measurements were carried out using a frequency analyzer (Impedance Analyzer 7260 from Material Mates) coupled to a Biologic ITS Peltier oven. This frequency analyzer allows measurements to be made at frequencies between 10^7^ and 1 Hz.

The measurements in the TP configuration were carried out using a CESH-e (Enhanced Controlled Environment Sample Holder) sample holder manufactured by Biologic, which is specially designed to measure the conductivities of solid materials and can withstand temperatures from -40 to 150 °C (**Figure S44**). The thin gold electrodes mounted on the sample holder are interchangeable. The distance between the electrodes is controlled by a PTFE spacer with a thickness of 50 µm and an internal diameter of 1.5 to 2.0 mm. Before each measurement, the thickness and diameter dimensions of each spacer are measured to ensure a more accurate determination of the cell constant.


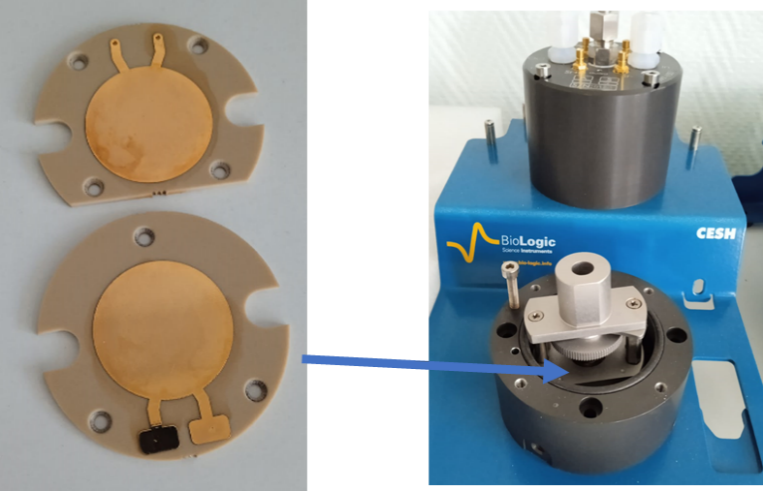

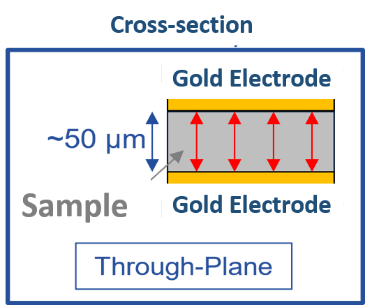


**Figure S44**: Photographs of two gold electrodes (left), the CESH cell (Biologic) specially designed for measuring the conductivity of solid materials (centre) and an illustration of the direction of measurement between the electrodes (right).

The **Figure S45** shows the Nyquist diagram of the [C_18_C_18_Im]^+^/[N(CN)_2_]⁻ TILC at 80 °C during the 1^st^ cooling cycle and its experimental fitting using equivalent circuit modelling. The measurements show a cable resistance value R_1_ of 10^-3^ Ohm. This value is negligible compared to the resistance value R_2_ corresponding to the resistance of the electrolyte (capacitance value of the order of 10^-10^ F) and a parameter value n (indicator of homogeneity within the system) close to 0.95 (homogeneous). The ionic conductivity value thus obtained is 4.4·10^-2^ mS·cm^-1^. The presence of a third resistivity value, R_3_ = 3762 Ohm, linked to the presence of interfaces (interdomains), is highlighted on the graph at lower frequency values (between 10^4^ and 10^2^ Hz). The conductivity value σ_3_ is equal to 1.3·10^-2^ mS·cm^-1^. These interfaces are more heterogeneous, with an index value n of 0.72. **Table S17** summarises the parameters extracted from the Nyquist plots at 80 °C during the 1^st^ cooling cycle.


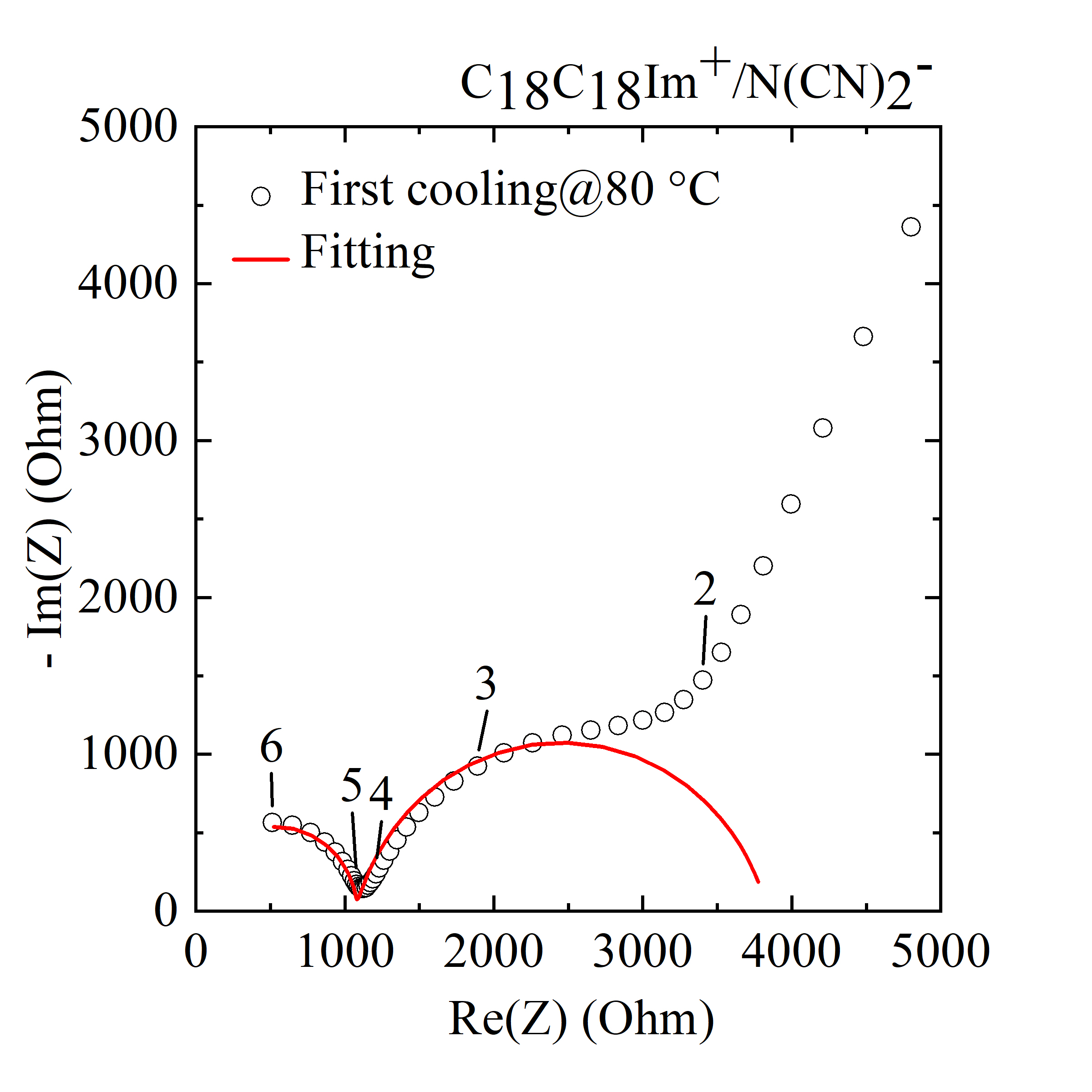


**Figure S45**. Adjusted Nyquist plot of the [C_18_C_18_Im]^+^/[N(CN)_2_]⁻ TILC at 80 °C during the 1^st^ cooling cycle. Note: *Numbers represent frequency decades from the nearest experimental point.*

**Table S17**. Parameters extracted from the Nyquist diagram of [C_18_C_18_Im]^+^/[N(CN)_2_]⁻ at 80 °C on first cooling.

| **R_1_ (Ohm)** | **R_2_ (Ohm)** | **Capacity 2 (F)** | **n_2_** | **R_3_ (Ohm)** | **Capacity 3 (F)** | **n_3_** |
| --- | --- | --- | --- | --- | --- | --- |
| 1.2·10^-3^ | 1073 | 1.53·10^-10^ | 1 | 2762 | 8.05·10^-6^ | 0.84 |

**Measurement of ion transport properties in the In-Plane (IP) configuration.**

In the In-Plane (IP) configuration, the cell studied has a structure consisting of two arrays of interdigitated electrode strips, also known as "IDE" for "interdigitated electrodes". These electrodes are marketed by Micrux Technologies and consist of a thin layer of platinum (Pt), as shown in **Figure S46**. The commercial designation of this particular cell is "ED-IDE1-Pt". The interdigitated electrode strips are designed with a constant width of 10 µm and, the distance between the electrodes is also equal to 10 µm. The electrodes are deposited as thin layers onto a glass substrate.

The dimensions of the cell are 10 mm long, 6 mm wide and, 0.75 mm thick, with an electrolyte deposition area of 3.50 mm in diameter. A key aspect of the cell design is the incorporation of a protective insulating polymer layer to prevent sample spillage outside the electrodes. The cell can hold between 2 and 10 µL of sample.


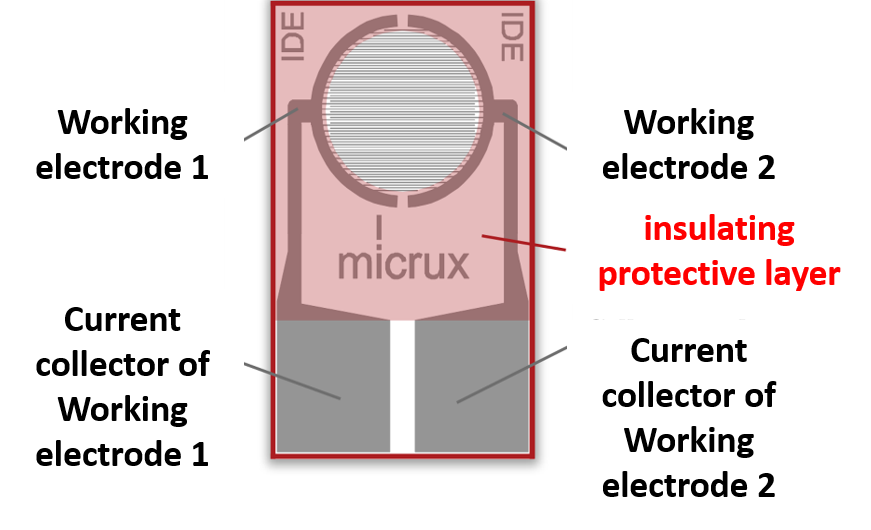


**Figure S46**: Diagram of an electrochemical cell with interdigitated electrodes in the IP configuration for EIS measurements.

In this IP configuration, orthogonal to the TP configuration, EIS measurements are performed in the horizontal plane of a cell using interdigitated electrodes. These electrodes are positioned on the surface of the cells to take transport measurements parallel to their surface. Prior to each measurement, the TILCs are heated above their clearing temperature under a dynamic primary vacuum (in a Büchi tube furnace) to ensure appropriate wetting at the interfaces with the IDEs.

The trends in ionic conductivity values are shown in **Figure S47**. The measurements show relatively low conductivity values at high temperatures, as follows 1·10^-4^ mS·cm^-1^ ([C_18_C_18_Im]^+^/Br^-^) > 4.6·10^-5^ mS·cm^-1^ ([C_18_C_18_Im]^+^/[N(CN)_2_]⁻) > 3.7·10^-5^ mS·cm^-1^ ([C_18_C_18_Im]^+^/I^-^) at 120 °C and these values decrease significantly with decreasing temperature by following the order: 4.8·10^-6^ mS·cm^-1^ ([C_18_C_18_Im]^+^/[N(CN)_2_]⁻) > 3.6·10^-6^ mS·cm^-1^ ([C_18_C_18_Im]^+^/Br^-^) > 3.6·10^-7^ mS·cm^-1^ ([C_18_C_18_Im]^+^/I^-^) at 80 °C.


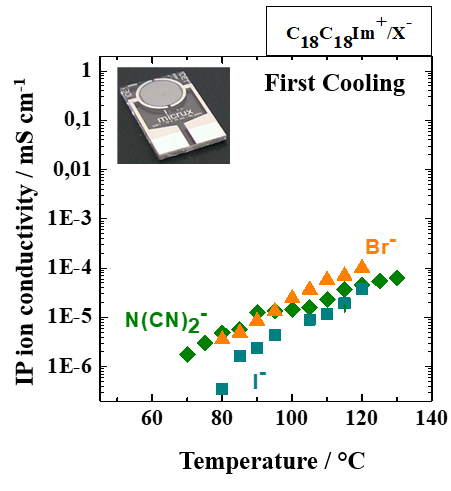


**Figure S47**. Changes in ionic conductivity in the IP configuration as a function of temperature for the [C_18_C_18_Im]^+^/Br^-^, [C_18_C_18_Im]^+^/I^-^, and [C_18_C_18_Im]^+^/[N(CN)_2_]⁻ TILCs

**Anisotropy of ion transport properties.**

From the results obtained using the two measurement configurations (TP *vs.* IP), it is possible to access the anisotropy of the ionic transport properties. Anisotropy in this context refers to the difference in conductivity levels observed in transport properties evaluated in directions perpendicular to each other. Anisotropy is expressed as the ratio between the conductivity values in TP vs. IP configurations (σ_TP_/σ_IP_).

However, before commenting on the anisotropy, it is mandatory to ensure that the TILC orientation is homogeneous or homeotropic in both cell configurations. If the alignment of the TILCs in the two configurations is not identical, the anisotropy of the ionic conductivity would be affected. It is therefore crucial to ensure and confirm the alignment of the TILCs in the two cells to confirm the existence of anisotropy in the ionic transport properties.

At a temperature of 115 °C, the anisotropy value for the [C_18_C_18_Im]^+^/[N(CN)_2_]⁻ TILC is significantly higher (~3749) than that for the bromide (~537) and iodide (~163). The dicyanamide anion was shown to be more confined than halide anions in their SmA_d_ mesophases. As the specific interactions between ions are different for each anion, the bulkier and confined dicyanamide anion favours a higher degree of orientation and therefore greater anisotropy of ion transport compared to smaller anions such as Br^-^ and I^-^ anions. Furthermore, these measured anisotropy values (~10^2^ to ~10^4^) are significant in terms of organisation.

The gradual decrease in anisotropy with increasing temperature can be interpreted as a consequence of the increasing fluidisation of the mesophases associated with greater thermal agitation, which disrupts the ordered structure of the mesophase, allowing the ions to move more freely. The anisotropy then tends to decrease until it disappears at higher temperatures, signalling the completion of the transition to the isotropic phase. These results confirm the microscopic observations in POM and show the homogeneous orientation of the ionic layers on the surface of the electrodes (see **Figure S48**).


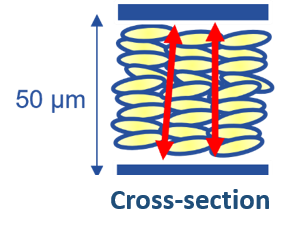

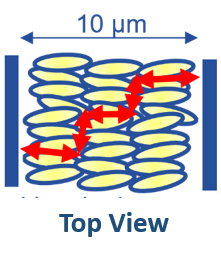


**Figure S48**. Schematic representation of the homogeneous alignment of SmA_d_ layers on the surface of gold electrodes in TP configuration (cross-sectional view) (left) and in IP configuration for Pt electrodes (top view) (right). The red arrows represent the diffusion of ions through the ionic sublayer (left) and the interlamellar diffusion of excess ions (right).

In the SmA_d_ mesophases of these TILCs, the ions diffuse preferentially in the ionic sub-layers, in an orientation perpendicular to the electrodes (a "hopping" diffusion mechanism, which is more important in the TP configuration). In contrast, the diffusion parallel to the layers is reduced (the interlamellar vehicular transport mechanism predominates in the IP configuration). Explained by Cherian *et al*.^[[38]](#footnote-38)^, this phenomenon is due to the diffusion of ions through defects, also known as "leakage channels", which form within the ionic sub-layers. An increasing number of these leakage channels increases the ease with which ions can escape their 2D diffusion slabs. This observation suggests that ion transport in smectic mesophases is, in fact, of a quasi-2D nature (*and not 2D in a defect free, idealised lamellar organisation*) to account for these leakage channels.

**16. Statistical Analysis**

Electrochemical impedance spectroscopy (EIS) measurements were conducted in multiple independent series under controlled thermal and alignment conditions. The relative error across repeated measurements was estimated to be within 20%. No formal statistical hypothesis testing was applied. Data are presented as measured or as average values when multiple runs were available.

Polarized optical microscopy (POM) was used for qualitative observation of textures and phase transitions. No quantitative image analysis or statistical treatment was applied.

Thermal analysis (DSC and TGA) was performed on different instruments using independent sample preparations and heating protocols to assess reproducibility. No standard deviation or statistical comparison was computed for these measurements.

SWAXS measurements were performed on multiple beamlines, including on EBS-ESRF beamlines and an in-house set-up, using consistent sample preparation protocols. Although no formal statistical tests were applied, reproducibility was ensured across instruments and measurement runs. Data processing and visualization were carried out with Python-based SAXS/WAXS platforms.

All data processing and visualization were performed using OriginPro 2024, ZView, Python (Jupyter Notebooks), and instrument-specific software as applicable.

1. Mitov, M. *Chem. Phys. Chem.* **2014**, *15*, 1245-1250 [[10.1002/cphc.201301064](https://doi.org/10.1002/cphc.201301064)]. [↑](#footnote-ref-1)
2. For further (in-depth information) reading related to the states of matter and liquid crystal and their mesophases, see: Bloch, J. *et al*. *Nat. Rev. Phys.* **2022***, 4*, 470–488. [[10.1038/s42254-022-00464-0](https://doi.org/10.1038/s42254-022-00464-0)]; Kato, T.; Uchida, J.;Ichikawa, T.; Sakamoto, T*. Angew. Chem. Int. Ed.* **2018**, *57*, 2-19. [[10.1002/anie.201711163](https://doi.org/10.1002/anie.201711163)]. [↑](#footnote-ref-2)
3. Stappert, K.; Ünal, D.; Mallick, B.; Mudring, A.-V. *J. Mater. Chem. C.* **2014,** *2(37)*, 7976-7986. [[10.1039/C3TC31366B](https://dx.doi.org/10.1039/C3TC31366B)]. [↑](#footnote-ref-3)
4. McDaniel, J. G.; Son, C. Y.; Yethiraj, A. *J. Phys. Chem. B.* **2018**, *122*(14), 4101-4114. [[10.1021/acs.jpcb.8b01221](https://dx.doi.org/10.1021/acs.jpcb.8b01221)].

   Ishisone, K.; Ori, G.; Boero, M. *Phys. Chem. Chem. Phys.* **2022***,* *24*(16), 9597‑9607. [[10.1039/D2CP00741J](https://dx.doi.org/10.1039/D2CP00741J)]. [↑](#footnote-ref-4)
5. For the structural design of ionic liquids in the literature, see: Hu, J.; Zhang, D.; Jin, S.; Cheng, S. Z. D.; Harris, F. W. *Chem. Mater.* **2004**, *16*, 4912-4915 [[10.1021/cm0492179](https://doi.org/10.1021/cm0492179)]. For a review about liquid crystals and chemical structures, see: Roy, B.; De, N.; Majumdar, K. C*. Chem. Eur. J.* **2012**, *18*, 14560-14588 [[10.1002/chem.201200483](https://doi.org/10.1002/chem.201200483)]. [↑](#footnote-ref-5)
6. Public prices consulted on the website of Merck/Sigma-Aldrich. [↑](#footnote-ref-6)
7. Ion exchange reactions were challenging since the removal of inorganic impurities requires extraction with water. This triggers losses in the purified ionic liquid during the extraction process [↑](#footnote-ref-7)
8. Zhao, H.; Foss, F. W.; Breslow, R. *J. Am. Chem. Soc*. **2008**, *38*, 12590-12591 [[10.1021/ja804577q](https://doi.org/10.1021/ja804577q)]. [↑](#footnote-ref-8)
9. Soares, B. G.; Livi, S.; Duchet-Rumeau, J.; Gerard, J. F. *Polymer* **2012**, *53*, 60-66 [[10.1016/j.polymer.2011.11.043](https://doi.org/10.1016/j.polymer.2011.11.043)] [↑](#footnote-ref-9)
10. Livi, S.; Gerard, J.; Duchet-Rumeau, J. *Chem. Commun*. **2011**, *47*, 3589-3591 [[10.1039/C0CC04273K](https://doi.org/10.1039/C0CC04273K)]. [↑](#footnote-ref-10)
11. Spectroscopic data is in agreement with reported literature, see: Livi, S.; Gerard, J; Duchet-Rumeau, J*. Chem. Commun*. **2011**, *47*, 3589-3591 [[10.1039/C0CC04273K](https://doi.org/10.1039/C0CC04273K)]. [↑](#footnote-ref-11)
12. Molecule reported in the literature, see: Tan, C. *J. Mol. Struct.* **2017**, *1148*, 34-39 [[10.1016/j.molstruc.2017.07.018](https://doi.org/10.1016/j.molstruc.2017.07.018)]. [↑](#footnote-ref-12)
13. Dinarès, I.; de Miguel, C. G.; Ibáñez, A.; Mesquida, N.; Alcalde, E.; *Green Chem*. **2009**, *11*, 1507-1510 [[10.1039/B915743N]](https://doi.org/10.1039/B915743N). [↑](#footnote-ref-13)
14. Naert, P.; Rabaey, K.; Stevens, C. V*. Green Chem.* **2018**, *20*, 4277-4286 [[10.1039/C8GC01869C]](https://doi.org/10.1039/C8GC01869C). [↑](#footnote-ref-14)
15. Molecule not reported in the literature. [↑](#footnote-ref-15)
16. This molecule has not been reported in the literature. However, a patent reports the improvement of the material properties that had the ionic liquid incorporated. The mentionned improved properties were: increased surface electrical conductivity and abrasion resistance. See: US20200041920A1 [[link](https://worldwide.espacenet.com/patent/search/family/069228586/publication/US2020041920A1?q=US20200041920A1)] [↑](#footnote-ref-16)
17. Maton, C.; De Vos, N.; Stevens, C. V. *Chem. Soc. Rev.* **2013**, *42*, 5963-5977 [[10.1039/C3CS60071H](https://doi.org/10.1039/C3CS60071H)]. [↑](#footnote-ref-17)
18. Huddleston, J. G.; Visser, A. E.; Reichert, W. M.; Willauer, H. D.; Broker, G. A.; Rogers, R. D. *Green Chem*. **2001**, *3*, 156-164 [[10.1039/B103275P](https://doi.org/10.1039/B103275P)]. [↑](#footnote-ref-18)
19. Rogers, R. D.; Seddon, K. R. *Science* **2003**, 302(5646), 792-793 [[10.1126/science.1090313](https://doi.org/10.1126/science.1090313)] [↑](#footnote-ref-19)
20. Goossens, K., Wellens, S., Van Hecke, K., Van Meervelt, L., Cardinaels, T., Binnemans. K. T-Shaped Ionic Liquid Crystals Based on the Imidazolium Motif: Exploring Substitution of the C-2 Imidazolium Carbon Atom. *Chem. Eur. J.* **2011**, 17(15), 4291‑4306 [[10.1002/chem.201001921](https://doi.org/10.1002/chem.201001921)]. [↑](#footnote-ref-20)
21. Dadivanyan, A. K., Noah, O. V., Pashinina, Yu. M., Belyaev, V. V., Chigrinov, V. G., Chausov, D. N. Anchoring Energy of Liquid Crystals. *Mol. Cryst. and Liq.* **2012**, 560(1), 108‑114 [[10.1080/15421406.2012.663185](https://dx.doi.org/10.1080/15421406.2012.663185)]. [↑](#footnote-ref-21)
22. Gear, C., Diest, K., Liberman, V., Rothschild, M., Engineered liquid crystal anchoring energies with nanopatterned surfaces. *Opt. Express* **2015**, 23(2), 807-814 [[10.1364/OE.23.000807](https://doi.org/10.1364/OE.23.000807)]. [↑](#footnote-ref-22)
23. Choi, Y., Yokoyama, H., Gwag, J. S., Determination of surface nematic liquid crystal anchoring strength using nano-scale surface grooves. *Opt. Express,* **2013**, 21(10), 12135-12144 [[10.1364/OE.21.012135](https://doi.org/10.1364/OE.21.012135)]. [↑](#footnote-ref-23)
24. Kieffer, J., Karkoulis, D., PyFAI, a versatile library for azimuthal regrouping. *J. Phys.: Conf. Ser.* **2013**, 425, 202012 [[10.1088/1742-6596/425/20/202012](https://doi.org/10.1088/1742-6596/425/20/202012)]. [↑](#footnote-ref-24)
25. Langford, J. I.; Wilson, A. J. C. *J. Appl. Crystallogr.* **1978**, *11*, 102-113 [[10.1107/S0021889878012844](https://doi.org/10.1107/S0021889878012844)] [↑](#footnote-ref-25)
26. Kouwer, P. H. J.; Swager, T. M. *J. Am. Chem. Soc.* **2007**, *129*(45), 14042–14052 [[10.1021/ja075651a](https://dx.doi.org/10.1021/ja075651a)] [↑](#footnote-ref-26)
27. Canongia Lopes, J. N. A., Pádua, A. A. H., Nanostructural Organisaisation in Ionic Liquids. *J. Phys. Chem. B* **2006***, 110*(7), 3330–3335 [[10.1021/jp056006y](https://doi.org/10.1021/jp056006y%20)].

    Pádua, A. A. H., Resolving dispersion and induction components for polarisable molecular simulations of ionic liquids. *J. Chem. Phys.* **2017***, 146*(20), 204501 [[10.1063/1.4983687](https://doi.org/10.1063/1.4983687%20)].

    Merlet, C., Salanne, M., Rotenberg, B., New Coarse-Grained Models of Imidazolium Ionic Liquids for Bulk and Interfacial Molecular Simulations. *J. Phys. Chem. C* **2012***, 116*(14), 7687–7693 [[10.1021/jp3008877](https://doi.org/10.1021/jp3008877%20)].

    Heid, E., Szabadi, A., Schröder, C., Quantum mechanical determination of atomic polarisaisabilities of ionic liquids. *Phys. Chem. Chem. Phys.* **2018***, 20*(16), 10992–10996 [[10.1039/C8CP01677A](https://dx.doi.org/10.1039/C8CP01677A)]. [↑](#footnote-ref-27)
28. Goloviznina, K.; Canongia Lopes, J.N.; Costa Gomes, M.; Pádua, A.A.H. Transferable, Polarisaisable Force Field for Ionic Liquids. *J. Chem. Theory Comput.* **2019**, 15, 5858-5871. [[10.1021/acs.jctc.9b00689](https://doi.org/10.1021/acs.jctc.9b00689)].

    Goloviznina, K., Gong, Z., Costa Gomes, M. F., Pádua, A. A. H., Extension of the CL&Pol Polarisaisable Force Field to Electrolytes, Protic Ionic Liquids, and Deep Eutectic Solvents. *J. Chem. Theory Comput.* **2021***,* 17(3), 1606–1617. [[10.1021/acs.jctc.0c01002](https://doi.org/10.1021/acs.jctc.0c01002)].

    Goloviznina, K., Gong, Z., Padua, A. A. H., The CL&Pol polarizable force field for the simulation of ionic liquids and eutectic solvents. *Wiley Interdiscip. Rev. Comput. Mol. Sci.* **2022**, 12, e1572. [[10.1002/wcms.1572](https://doi.org/10.1002/wcms.1572)]. [↑](#footnote-ref-28)
29. <https://openmm.org>

    Eastman, P., Friedrichs, M. S., Chodera, J. D., et al., OpenMM 4: A Reusable, Extensible, Hardware Independent Library for High Performance Molecular Simulation. *J. Chem. Theory Comput.* **2013**, 9, 461. [[10.1021/ct300857j](https://doi.org/10.1021/ct300857j)]. [↑](#footnote-ref-29)
30. Goossens, K., Wellens, S., Van Hecke, K., Van Meervelt, L., Cardinaels, T., Binnemans, K., T-Shaped Ionic Liquid Crystals Based on the Imidazolium Motif: Exploring Substitution of the C-2 Imidazolium Carbon Atom. *Chem. Eur. J.* ***2011****, 17(15),* 4291‑4306 [[10.1002/chem.201001921](https://doi.org/10.1002/chem.201001921)]. [↑](#footnote-ref-30)
31. Ichikawa, T., Yoshio, M., Hamasaki, A., Kagimoto, J., Ohno, H., Kato, T., 3D Interconnected Ionic Nano-Channels Formed in Polymer Films: Self-Organisaisation and Polymerisaisation of Thermotropic Bicontinuous Cubic Liquid Crystals. *J. Am. Chem. Soc.* ***2011****, 133*(7), 2163‑2169 [[10.1021/ja106707z](https://doi.org/10.1021/ja106707z)]. [↑](#footnote-ref-31)
32. Park, G., Goossens, K., Shin, T. J., Bielawski, C. W. Dicyanamide Salts that Adopt Smectic, Columnar, or Bicontinuous Cubic Liquid-Crystalline Mesophases. *Chem. Eur. J.* **2018***, 24*(24), 6399‑6411 [[10.1002/chem.201705794](https://doi.org/10.1002/chem.201705794)]. [↑](#footnote-ref-32)
33. Durham, P. J., Galemmo, R. A., The introduction of a new sulfamoylation reagent: N-carbo-(trimethylsilyloxy)sulfamoylchloride. Versatile syntheses of 3-amino-4-n-alkyl and 3-amino-2-n-alkyl-5-aryloxy-1,2,4,6-thiatriazine-1,1-dioxides. *Tetrahedron Lett.* **1986***, 27*(2), 123‑126 [[10.1016/S0040-4039(00)83957-X](https://doi.org/10.1016/S0040-4039(00)83957-X)]. [↑](#footnote-ref-33)
34. Park, G., Goossens, K., Shin, T. J., Bielawski, C. W. Dicyanamide Salts that Adopt Smectic, Columnar, or Bicontinuous Cubic Liquid-Crystalline Mesophases. *Chem. Eur. J.* **2018***, 24*(24), 6399‑6411 [[10.1002/chem.201705794](https://doi.org/10.1002/chem.201705794)]. [↑](#footnote-ref-34)
35. Vazquez-Salazar L.I., Selle M., de Vries A.H., Marrink S.J., Souza P.C.T., Martini coarse-grained models of imidazolium-based ionic liquids: from nanostructural organisaisation to liquid–liquid extraction, *Green Chem*. **2020**, *22*, 7376-7386. [[10.1039/D0GC01823F](http://doi.org/10.1039/D0GC01823F)].

    Souza P.C.T., Alessandri R., Barnoud J., Thallmair S., Faustino I., Grünewald F., Patmanidis I., Abdisaisadeh H., Bruininks B.M.H., Wassenaar T.A., Kroon P.C., Melcr J., Nieto V., Corradi V., Khan H.M., Domański J., Javanainen M., Martinez-Seara H., Reuter N., Best R.B., Vattulainen I., Monticelli L., Periole X., Tieleman D.P., de Vries A.H., Marrink S.J., Martini 3: a general purpose force field for coarse-grained molecular dynamics, *Nat. Methods* **2021**, *18*, 382-388. [[10.1038/s41592-021-01098-3](https://doi.org/10.1038/s41592-021-01098-3)]. [↑](#footnote-ref-35)
36. MacFarlane, D. R., Forsyth, M., Izgorodina, E. I., Abbott, A. P., Annat, G., Fraser, K. On the concept of ionicity in ionic liquids. *Phys. Chem. Chem. Phys.* **2009***, 11*(25), 4962-4967 [[10.1039/b900201d](https://dx.doi.org/10.1039/b900201d%20)].

    Ueno, K., Tokuda, H., Watanabe, M. Ionicity in ionic liquids: Correlation with ionic structure and physicochemical properties. *Phys. Chem. Chem. Phys.* **2010** *12*(8), 1649-1658. [[10.1039/b921462n](https://dx.doi.org/10.1039/b921462n)].

    Vila, J., Ginés, P., Pico, J. M., Franjo, C., Jiménez, E., Varela, L. M., Cabeza, O. Temperature dependence of the electrical conductivity in EMIM-based ionic liquids. *Fluid Ph. Equilib.* **2006**,242(2), 141‑146 [[10.1016/j.fluid.2006.01.022](https://dx.doi.org/10.1016/j.fluid.2006.01.022)]. [↑](#footnote-ref-36)
37. Gaberšček, M. Understanding Li-based battery materials *via* electrochemical impedance spectroscopy. *Nat. Commun.* **2021**, *12*(1), 6513 [[10.1038/s41467-021-26894-5](https://doi.org/10.1038/s41467-021-26894-5)].

    Mei, B.-A., Munteshari, O., Lau, J., Dunn, B., Pilon, L., Physical Interpretations of Nyquist Plots for EDLC Electrodes and Devices. *J. Phys. Chem. C* **2018***, 122*(1), 194–206 [[10.1021/acs.jpcc.7b10582](https://doi.org/10.1021/acs.jpcc.7b10582)].

    Wang, S., Zahng J., Gargi O., Vivier V., Gao M., Orazem M. E. Electrochemical impedance spectroscopy. *Nat Rev Methods Primers* **2021**, *1*, 42 [[10.1038/s43586-021-00045-y](https://doi.org/10.1038/s43586-021-00045-y)]. [↑](#footnote-ref-37)
38. Cherian, T., Nunes, D. R., Dane, T. G., Jacquemin, J., Vainio, U., Myllymäki, T. T. T., Timonen, J. V. I., Houbenov, N., Maréchal, M., Rannou, P., Ikkala, O. Supramolecular Self‐Assembly of Nanoconfined Ionic Liquids for Fast Anisotropic Ion Transport. *Adv. Funct. Mater.* **2019***,* 29(49), 1905054 [[10.1002/adfm.201905054](https://doi.org/10.1002/adfm.201905054)]. [↑](#footnote-ref-38)
